# Common Contaminants in Next-Generation Sequencing That Hinder Discovery of Low-Abundance Microbes

**DOI:** 10.1371/journal.pone.0097876

**Published:** 2014-05-16

**Authors:** Martin Laurence, Christos Hatzis, Douglas E. Brash

**Affiliations:** 1 Shipshaw Labs, Montreal, Quebec, Canada; 2 Yale Comprehensive Cancer Center, Yale School of Medicine, New Haven, Connecticut, United States of America; 3 Department of Therapeutic Radiology, Yale School of Medicine, New Haven, Connecticut, United States of America; Natural History Museum of Denmark, Denmark

## Abstract

Unbiased high-throughput sequencing of whole metagenome shotgun DNA libraries is a promising new approach to identifying microbes in clinical specimens, which, unlike other techniques, is not limited to known sequences. Unlike most sequencing applications, it is highly sensitive to laboratory contaminants as these will appear to originate from the clinical specimens. To assess the extent and diversity of sequence contaminants, we aligned 57 “1000 Genomes Project” sequencing runs from six centers against the four largest NCBI BLAST databases, detecting reads of diverse contaminant species in all runs and identifying the most common of these contaminant genera (*Bradyrhizobium*) in assembled genomes from the NCBI Genome database. Many of these microorganisms have been reported as contaminants of ultrapure water systems. Studies aiming to identify novel microbes in clinical specimens will greatly benefit from not only preventive measures such as extensive UV irradiation of water and cross-validation using independent techniques, but also a concerted effort to sequence the complete genomes of common contaminants so that they may be subtracted computationally.

## Introduction

Systematic pathogen discovery based on unbiased high-throughput sequencing [1] was first used in 2008 to detect two novel viruses by pyrosequencing clinical specimens. The first study attributed 2 fragments out of 395,734 (0.0005%) to a novel polyomavirus [2]; the second study attributed 14 fragments out of 103,632 (0.0135%) to a novel arenavirus [3]. Recent improvements in sequencing technology have rendered this method much more sensitive for detecting low-abundance pathogens and other medically important microbes in clinical specimens. For example, PathSeq [4], a bioinformatics toolkit designed to detect novel sequences, was recently used to identify a novel *Bradyrhizobium* species in clinical specimens using whole metagenome shotgun DNA libraries and the Illumina sequencing platform, which can currently produce up to 186 million read pairs per lane [5]. A novel infectious species representing only 0.000001% of the total DNA in a clinical specimen can theoretically be detected using a single Illumina HiSeq 2000 lane, since at least one read pair out of 186 million would originate from the novel microbe in 84% of runs.

Unbiased high-throughput sequencing has been suggested as a way of detecting etiological microbes in cancer tissue [6], an approach we consider promising for prostate cancer [7], [8]. To facilitate such studies we constructed the Leif Microbiome Analyzer, a bioinformatics tool similar to PathSeq which was designed to eliminate the need for cluster computing typically required by NCBI blastn based tools such as PathSeq–even when aligning against the largest NCBI BLAST databases. A basic assumption in calculating the sensitivity of such approaches is that one microbial read is informative [6]. However, while testing the Leif Microbiome Analyzer by examining two clinical samples for reads not aligning to the human genome, we encountered many reads from diverse species not known to be part of the human microbiome, suggesting the presence of contamination; members of the *Bradyrhizobium* genus were particularly prominent. If this situation arises commonly, it would be cost-prohibitive to screen candidate non-aligning reads using polymerase chain reaction (PCR) on the original specimens. For example, 1000 novel microbe reads would entail 1000 confirmatory PCR reactions on the original tissue sample; at $100 each, including custom primers and optimization, the $100,000 validation cost would greatly exceed the $2500 cost of one Illumina HiSeq 2500 lane and would be prone to error. As the cost of sequencing continues to decline faster than the cost of PCR, this overhead is expected to worsen. In the case of novel contaminant microbes whose genome has not been completely sequenced, the 1000 confirmatory PCR reaction figure cannot be reduced by sampling only some reads of each species, as it is not possible to know which reads arose from the same species.

Various types of contamination in sequencing runs have been reported before [9], [10], as well as a few mitigation techniques [11]–[13] which are not suitable for the discovery of novel microbes. We therefore inquired whether contamination was restricted to our libraries or sequencing center, or is a general property of next-generation sequencing workflows.

## Materials and Methods

### Sample Selection

Initial sequence analysis was performed on two human prostate samples which are not reported here but which motivated the larger studies of publicly available genomic sequences from cultures expected to contain cells of a single species. To obtain sequences of healthy human cells generated by next-generation sequencing workflows which closely resembled our own samples and runs, we searched the NCBI Sequence Read Archive database for “1000 genomes project AND hiseq AND CEU”. The search was restricted to Illumina HiSeq because the Leif Microbiome Analyzer is optimized for this platform; CEU are genomes of Northern European ancestry. Paired-end whole genome shotgun sequencing runs done for the 1000 Genomes Project [14] used DNA extracted from cultured Epstein-Barr virus immortalized B-lymphocytes drawn from blood of healthy individuals. A full description of specimens can be found at www.ncbi.nlm.nih.gov/sra for each of the runs used. This search gave 44 runs, some of which were too small (<18 M read pairs), had reads that were too short (<2×100), or crashed when converting to FASTQ format using the NCBI tool fastq-dump. Of the 44 runs, 23 passed these criteria. This initial search resulted in only Baylor College of Medicine and Broad Institute runs, so we then widened the search to runs from other sequencing centers. An SRA search for “hapmap AND hiseq AND CEU” resulted in 1298 runs. From these, runs from other sequencing centers were chosen randomly for a total of 57 runs. These include sequencing centers from the US, UK, and Germany. Non-human next-generation sequencing runs were also analyzed by searching the NCBI Sequence Read Archive database for “Candida albicans AND HiSeq AND WGS”; this search resulted in 198 runs, 142 of which were suitable for analysis (read pairs formatted as 2×100, >2M read pairs and *Candida albicans* species). All 142 runs were done at the Broad Institute.

### Sequence Alignment

For all nucleotide sequence alignments in this study, we used the Leif Microbiome Analyzer version 0.7.3 (www.shipshaw.com/leif), a short-read alignment tool that uses an algorithm similar to NCBI blastn and is optimized for paired-end reads produced by the Illumina platform. Reference sequences were obtained from the four largest NCBI BLAST databases (“nt”, “human_genomic”, “other_genomic” and “wgs”) downloaded in FASTA format on April 1^st^ 2014 from ftp.ncbi.nlm.nih.gov/blast/db/FASTA. These databases contain taxonomically labeled Genbank nucleotide sequences of organisms deemed BLAST-worthy by NCBI.

We screened NCBI BLAST eukaryotic sequences for possible *Bradyrhizobium* contamination by first randomly sampling *Bradyrhizobium* sequences found in the NCBI BLAST databases (simulating an Illumina sequencing run producing 200,000 2×100 read pairs), then aligning these read pairs against all eukaryotic sequences in the NCBI BLAST databases. Positive genomes involved sequencing centers from the US, Canada, and China. This screening technique is not exhaustive since only a random sampling of *Bradyrhizobium* sequences was used; aligning against all *Bradyrhizobium* sequences (or all bacterial sequences) is beyond the means of an academic research project. The list of commands required to replicate this analysis are provided in Text S1.

Publicly available Illumina HiSeq 2000 whole genome runs from the 1000 Genomes Project and *Candida albicans* runs were downloaded from the NCBI Sequence Read Archive database. Read pairs from these runs were aligned against the four largest NCBI BLAST databases, and contamination candidates were identified as described in the next section. The list of commands required to replicate this analysis are provided in Text S2 and S3.

### Identification of Contamination Candidates

The Leif Microbiome Analyzer performed the following ten steps to identify contamination candidates in 1000 Genome Project runs. 1) Bases whose quality letter was ≤ ‘%’ were deemed incorrectly called (‘N’). 2) Read pairs which contained many bases deemed to be incorrectly called were tagged as “low-quality” and discarded. Remaining read pairs satisfied the following criteria: ≥90% of bases in each read were deemed correctly called and ≥50% of the 32 base substrings in each read contained only bases deemed correctly called. 3) Read pairs which contained a 32 base substring matching exactly with human, EBV or phage sequences were discarded. 4) Read pairs which were nearly identical (bases 5 to 64 were compared and deemed nearly identical if ≥57 bases matched) were deemed to be clones produced as an artifact of library preparation and discarded. 5) The DUST algorithm [15] was used to mask bases which were deemed part of a low entropy sequence. 6) Read pairs which contained too many bases masked by DUST were tagged as “low-entropy” and discarded. Remaining read pairs satisfied the following criterion: ≥50% of the 32 base substrings in each read contained only unmasked bases. 7) Read pairs which contained an identical or reverse complement unmasked 32 base substring were merged into a “contig-like group”, and were deemed to have originated from the same template DNA strand pair. 8) A few read pairs from each “contig-like group” were randomly sampled for alignment; the number of sampled read pairs in each “contig-like group” was determined by the equation 1+ceiling(log2(number_of_read_pairs_in_group)). 9) Sampled read pairs were aligned to all sequences in the NCBI “nt”, “human_genomic”, “other_genomic” and “wgs” databases by Leif’s qblast command. Reads (mates) in each read pair were aligned both independently (single alignment) and together (dual alignment, which supports fragments of up to 1024 nucleotides in length). Single alignment was used to produce all results reported here except Text S4, S5, S6 and S7; though single alignment considers mates independently, mates are always assigned to the same “contig-like group”, so the link between mates is not completely lost. 10) “Contig-like groups” whose sampled read pairs aligned with primate, EBV or phage sequences were discarded. A “contig-like group” was deemed to have aligned to primate, EBV or phage sequences if ≥50% of sampled reads had ≥70% homology to primate, EBV or phage sequences present in the NCBI BLAST databases. For example, if a “contig-like group” contained three read pairs, then three out of the six reads must have ≥70% homology to primate, EBV or phage sequences to be discarded during this step.

**Figure 1:**
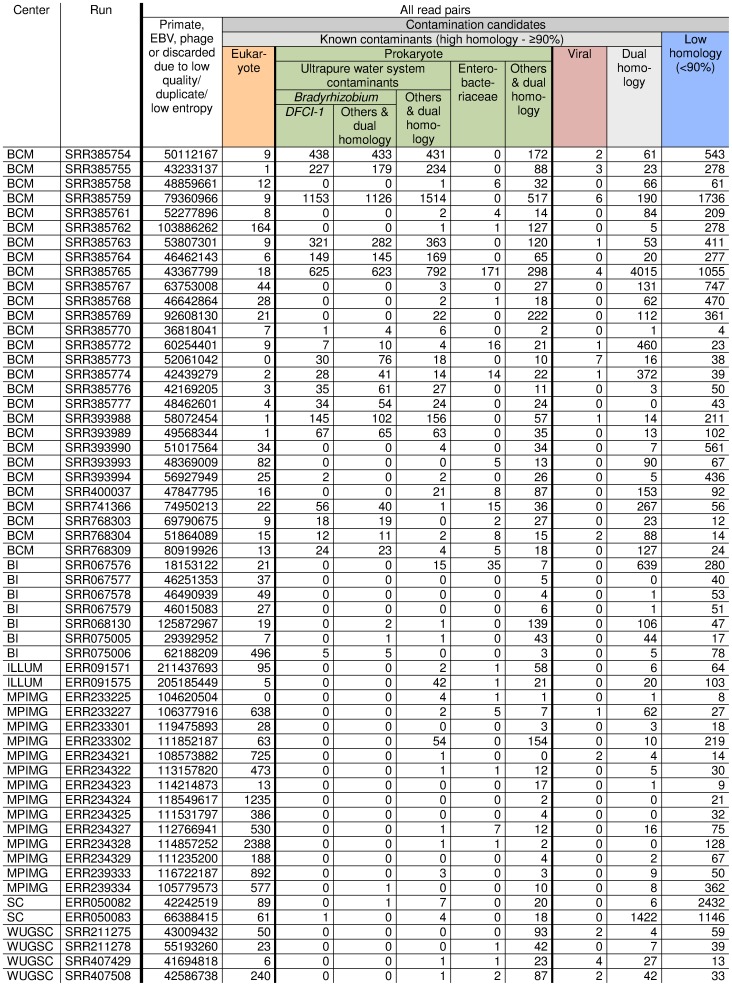
The contents of non-aligning reads from 57 human whole genome sequencing runs. Categories are defined in Methods. Sequencing center acronyms in this table are: Baylor College of Medicine (BCM), the Broad Institute (BI), Illumina (ILLUM), the Max Planck Institute for Molecular Genetics (MPIMG), the Sanger Center (SC), and Washington University Genome Sequencing Center (WUGSC). Runs are sorted alphabetically by center, then by SRA number which are assigned successively over time. Units are read pairs.

The consensus set produced by Leif reports the taxonomic node which encompasses all sequences aligning at a given homology percentage for each sampled read individually, and also merged for all sampled reads in a “contig-like group”. For example, in a “contig-like group” containing a single read pair, if read 1 has 93% homology to *Streptococcus mitis* and read 2 has 89% homology to *Enterococcus faecalis*, then the consensus taxonomic node for this “contig-like group” would be: 100%−94% = none, 93%−90% *Streptococcus mitis* and 89%−0% Lactobacillales–since Lactobacillales is the narrowest taxonomic node which contains both *Streptococcus mitis* and *Enterococcus faecalis*. Homology tests described below use the merged consensus set of each “contig-like group” to assign taxonomic classification to all read pairs within this group. “Contig-like groups” with a <90% homology to all database sequences were not given a taxonomic classification and were instead reported as “Low homology”: sampled read pairs in these groups either originated from a novel species/strain or contained sequence errors. “Contig-like groups” with ≥90% homology to any NCBI BLAST database sequence were deemed to be known contaminants. These were split into three broad categories: “Eukaryote”, “Prokaryote” and “Viral”. The “Dual homology” category contains “contig-like groups” with a high homology to two or more broad categories; since these are non-specific matches, they should not be reported as either “Eukaryote”, “Prokaryote” or “Viral”. Finally, the “Prokaryote” category was split into five parts. Many “contig-like groups” aligned specifically to ultrapure water system contaminants reported by Kulakov et al. [16] (genera *Bradyrhizobium*, *Rhizobium/Agrobacterium*, *Sphingomonas*, *Burkholderia*, *Ralstonia*, *Pseudomonas*, *Stenotrophomonas*, *Flavobacterium*) and to Enterobacteriaceae (probably to *Escherichia coli*, although the alignments were not sufficiently specific to exclude other Enterobacteriaceae species). Since *Bradyrhizobium* and *Bradyrhizobium sp. DFCI-1* were highly prevalent, they were reported in separate columns. For a “contig-like group” to be reported under a taxonomic name, all alignments to other taxonomic names were required to have a significantly lower homology (a 5% homology margin was chosen to generate Figure 1). For example, if a “contig-like group” had 93% homology to a eukaryote and 89% to a prokaryote (4% homology margin), its read pairs are reported under the taxonomic node “cellular organisms”, which is placed in the “Dual homology” category in Figure 1.

*Candida albicans* runs were analyzed similarly to human 1000 Genomes Project runs with the following differences: 1) the low quality bases were identified using ≤‘#’ (rather than ≤‘%’); 2) reads aligning to any sequence from the genus *Candida* were discarded (rather than reads aligning with any sequence from primates or EBV).

## Results

The presence of significant levels of *Bradyrhizobium* genus sequence in our two clinical samples led us to examine, as a negative control, reads from two human 1000 Genomes Project runs which did not align to the human genome. Manual review of Leif’s qblast results for these two runs (SRR768303 and SRR385759) aligned against the four largest NCBI BLAST databases revealed that about one third of non-human reads matched specifically with *Bradyrhizobium* sequences, especially *Bradyrhizobium sp. DFCI-1* (Text S4). In addition, some reads matched specifically to exotic species such as the Tibetan antelope, *Pantholops hodgsonii*. Further investigation of *Pantholops hodgsoni* nucleotide sequences in Genbank revealed that regions of this genome align very well with *Bradyrhizobium* sequences. The presence of *Bradyrhizobium* sequences in our two clinical specimens, in two randomly selected 1000 Genomes Project runs and in the assembled genome of *Pantholops hodgsonii* suggests that this bacterium is a common contaminant in next-generation sequencing workflows.

Randomly selected *Bradyrhizobium* sequences were then aligned against eukaryotic sequences in the NCBI BLAST databases, revealing many additional species which match specifically with *Bradyrhizobium* sequences (Table 1). It appears that *Bradyrhizobium* contamination may have been present in the sequencing runs used to assemble some genomes, such as the *Pantholops hodgsonii* genome, and were incorporated into assembled genomes in the NCBI Genome database. The problem is therefore not limited to searches for low-abundance microbes: contamination can go unrecognized in *de novo* assembled genomes, due to low coverage or inadequate curation. The seven Genbank entries listed in Table 1 suggest that *Bradyrhizobium* contamination is not limited to a single sequencing center, technology or eukaryotic species.

**Table 1:** Genbank entries which appear to be contaminated.

**Genbank sequence labeled as a eukaryote**	**Accession**	**Contig N50**	**Taxonomic label**	**Sequencing center**	**Sequencing** **platform**
**CCTCGACGTAGCGGATGACATCCTCAAGCTTGCG** **CTCGCGGAGGCGACGTTCGCCCAGATCCCAAAGCT** **CGATGTCCACCAGATAGGATGTCTGCGCGACGAAAT** **CCTCGACATCAGCGAAACCTTCTTCCCGCAAGCGTGG** **TCCGATCCGATCACGCGGCTCGACCGATCCAATGCTTT** **CGATCCCGGAGACGAAGTTGAC**	**AGTT01257760.1** **(737 to 938)**	18,674	*Pantholops hodgsoni*	Beijing Genomics Institute	Illumina GenomeAnalyser
	**APJD01000040.1** **(17553 to 17753)**	329,545	*Bradyrhizobium sp. OHSU_III*	Unknown	Illumina MiSeq
**ATCGGGAAGCGTCCGGGCTGGTAGCCGGGATTGACAT** **AGGTGACGTCGGCGATGCCGTCGCGCGCCATGTCGTA** **ATGATCGAAGGCCTTGCCGAGCTGCTGGGCCGGAAACA** **CCTTGCCGGTGATGGTGCCGCCGGAATCCTTGTTCACC** **GCAGCCACCCAGTCTTCCAGCGACTTCTGCAGCGGATGC** **GAGGCGGGCACCC**	**AUYS01000696.1** **(1109 to 1310)**	Unknown	*Melampsora pinitorqua*	Canada’s Michael Smith Genome Sciences Centre	Illumina HiSeq 2000
	**NC_017082.1** **(1001905 to 1002106)**	Complete	*Bradyrhizobium sp. S23321*	Unknown	454 GS FLX Titanium
**TTGATCCGGGCATTGTCCGCCGGGCGCAGCGGGCCCA** **GGCACTCCGCGGCCAGGATGAAGCGGGGTGAGGCGCG** **CAGCGCGATCTGCCGGCCGCCGAAATCGAGCGTTTGCA** **GATTGCCCTCGCCGGCGAGCGCCTGCACCGAGAACTCCA** **GGATCGCGGCCACCATCGCGCTGAGCAGATCGGCGCGC** **TCGTCGGGGGTCG**	**AHJH01004981.1** **(282 to 483)**	84,429	*Hammondia hammondi*	J. Craig Venter Institute	454 GS FLX Titanium and Illumina
	**AMFB01000038.1** **(11773 to 11974)**	141,525	B*radyrhizobium sp. DFCI-1*	Unknown	Illumina HiSeq 2000
**TCGGTTACGAATCCGATCGGCCTGGTTGTCGCTCAAAC** **GGCCGTCGGTGTCCCCGTCTTCAGTGGTCTCGGCGTTCG** **ACTGGCGGCGTTCGTCATTCTCCTGTCGGTCGGTGTTCTT** **TTCGTGCTGCATAAGACGGCGCGTCTGACGCTT**	**AADB02296177.1** **(1 to 150)**	Unknown	*Homo sapiens*	Celera	PE BiosystemsABI Prism
	**AMFB01000035.1** **(33174 to 33323)**	141,525	*Bradyrhizobium sp. DFCI-1*	Unknown	Illumina HiSeq 2000
**CGAGCCGCTCGGCGGCAGCGCGGATATCGTCCTTGGAGA** **GTGCCATGCGTGCTACACAAAAATGGATGCCGGGAAATGA** **TTAACATGTTAAGTGATTTCCCGCAAACTGAATCAGCGTAT** **GTTGCGGTGCAAAAGGTTGCGGCGGCGGATTAGGTGATGG** **CGCGCAAGAAATTGTCGAACGAGACAGGGCAGGAGCAGGG** **TGACGACACATCGTCGCGACGTGGCCCGATGCGCGAATTC** **TCACGTTCGCTGCCGATGTCGCTGCTCCGTGCGCGCGAGG** **CGGTGATGCGGCAGTTTCGTCCCTCGTTGCGCAATCACGG** **GCTGACCGAACAGCAAT**	**ADNL01003140.1** **(1 to 337)**	685	*Astrammina rara*	Unknown	454 GS FLX
	**AMFB01000042.1** **(23424 to 23760)**	141,525	*Bradyrhizobium sp. DFCI-1*	Unknown	Illumina HiSeq 2000
**AGACGCACTCGTTCGCGGAAGGATCGACCCAGCTCAAGAA** **CGGCCAGAGCTTCATCCTGGATTCCGACAAGACGCCGGGC** **GACAACAGCCGCGTCCAGCTTCCGCATCCGGAAATCCTCG** **CCGCGCTCCGCCCCGGCCACGCGCTGCTGCTCGACGACGG** **CAAGGTGCGGCTGATCGCCGAGGAGACCTCGCCCGAGCGC** **GCCGTGACGCGCGTCGTGATCGGCGGCAAGATGTCGGACC** **GCAAAGGCGTCAGCCTGCCCGACACCGATCTTCCCGTGTC** **CGCGATGACGCCGAAGGACCGTGCCGACCTCGAAGCTGCG** **CTGCCGGAGGGAATCGACTGGGTGGCGCTGTCCTCGTACA** **GCGGGCCGAGGACGTGATCGAGGCCAAGAAGATGATCCG** **CGGCCGCGCTGCCGTGATGGCCAAGATCGAGAAGCCTCAG** **GCGATCGACCGGCTCGCCGACATCATCGATGCGGCGGACG** **CGC**	**AKIR01004699.1** **(1 to 482)**	35,272	*Toxoplasma gondii*	J. Craig Venter Institute	454 GS FLX Titaniumand Illumina
	**AMFB01000031.1** **(95879 to 96354)**	141,525	*Bradyrhizobium sp. DFCI-1*	Unknown	Illumina HiSeq 2000
**AAGCGGCGCTGCATGTATTCGCCGAGGAATTTGCCGAGTG** **CGTCGGCCGCAAGTTCGGGAAGGGTCATCACGGCATTGAC** **CTCCGAGATCGGGTGGCGAGCTTACGGCCTGCCAGCATTT** **CAGGGTAGTCCCGCGATGTGACATAATCGTTGCGGCCGGA** **CCGAAGCGGCTGGACCGTAGTGACCCGTAATAATGCGGTG** **TGGGGTGGGAGCTGGTGGATGGGTGTCGATCTCTTGAACG** **TCAAAGGCTTGAGCGAGCTGGATCAAACGGCCCCGGTCGT** **GATGGTCAATCTGATGCGATTTCGCCAGCGGTCGCTCGAC** **GGCGACGGTTCGGGTTGGGATGCCTATCTGCGCTACAGCG** **CGCTCACTGTCCCCATGATCAAGGCCCGACGCGGGGCTAC** **GGGCTGCTCTGGACCGGCAACGCCGAGACGGTCGCGCTCG** **GCGAGCCGGACGGCCAGCGTTGGG**	**ADAS01038761.1** **(1 to 464)**	9,773	*Puccinia triticina*	Broad Institute	454; ABI
	**AMFB01000048.1** **(5318 to 5776)**	141,525	*Bradyrhizobium sp. DFCI-1*	Unknown	Illumina HiSeq 2000

Seven sequences which match specifically with both the *Bradyrhizobium* genus and a Genbank entry of a eukaryote. Two hundred thousand randomly selected *Bradyrhizobium* sequences were aligned against eukaryotes in the NCBI BLAST databases: this search is therefore indicative rather than exhaustive. *Bradyrhizobium* contamination inserted prior to *de novo* assembly of the eukaryote genome appears to have caused this double match, as it is not likely that different parts of the *Bradyrhizobium* genome would be conserved in select eukaryotes.

To compare a large number of whole genome shotgun sequencing libraries processed at different sequencing centers by a standardized protocol and not expected to contain etiologic microbes or be complicated by issues of handling of pathology samples, we used the Leif Microbiome Analyzer to identify non-aligning reads from 57 Illumina HiSeq 2000 runs performed by various sequencing centers on 1000 Genomes Project samples. The results are shown in Figure 1. Known contaminant sequences (defined here as read pairs in a human sample which match specifically with NCBI BLAST sequences other than primate, EBV, and phage) were present in all runs, varying from 0.000007% to 0.015% of total read pairs with a median of 0.0003%. Low homology sequences (defined here as read pairs which did not match with sequences in the NCBI BLAST databases, usually due to either a high number of sequencing errors or to the presence of novel contaminant strains/species in the run) are listed in a separate column and are not counted as known contaminant sequences–although some may well originate from novel contaminants. Eukaryotic DNA contamination was common, typically aligning to the genus *Bos*, which may be originating from fetal calf serum used in cell culture media. *Bradyrhizobium* contamination was found in 25 out of 57 runs. This particular contaminant varied from center to center (Figure 1). The highest levels of *Bradyrhizobium* were found in runs from center BCM, where 19 runs out of 30 were contaminated, reaching levels as high as 0.003% of reads (Figure 1). Some runs from centers SC, BI, and MPIMG contained a few reads which matched specifically with *Bradyrhizobium sp. DFCI-1* (Figure 1 and Text S5, S6 and S7). No *Bradyrhizobium* read pairs were found in two runs submitted by Illumina or four runs submitted by WUGSC. However, runs from these centers did show contamination from other organisms. Other species commonly encountered included genera *Rhizobium/Agrobacterium*, *Sphingomonas*, *Burkholderia*, *Ralstonia*, *Pseudomonas*, *Stenotrophomonas*, *Flavobacterium* (reported together in column “Ultrapure water system contaminants – Other” of Figure 1); sequence alignments to all species are reported in Spreadsheet S1. These 57 sequencing runs indicate that contamination is widespread and *Bradyrhizobium sp. DFCI-1* is a prominent contaminant, but contamination levels are highly variable between runs–even runs from the same sequencing center.

Alignment results from 142 *Candida albicans* sequencing runs performed using Illumina HiSeq 2000 are reported in Table S1 and in Spreadsheet S2. *Bradyrhizobium* contamination can be found in 136 out of 142 runs, reaching levels as high as 0.0126% of reads. Ultrapure water system contaminants were detected in all runs.

## Discussion

Many different microbe discovery techniques have been developed over the last 150 years [1]; the advent of next-generation sequencing technology has enabled molecular detection techniques which allow the discovery of fastidious or unculturable microbes in samples containing mixed flora such as the human skin [17]. These new techniques revolutionized our understanding of the human microbiome, revealing many previously unknown species in clinical specimens from healthy individuals.

The most common technique in use today for microbiome surveys is based on selective amplification of small regions of microbial DNA using consensus PCR primers prior to high-throughput sequencing. This technique has three major advantages over unbiased high-throughput sequencing: 1) most human DNA is eliminated prior to sequencing, which reduces sequencing costs for equal assay sensitivity; 2) only a single region of the genome is amplified, so aligning to all known sequences is not required, reducing alignment time approximately one hundred thousand fold; 3) only a single region of the genome is amplified, allowing the abundance of each novel species/sequence to be quantified by counting identical reads, rather than by doing a custom quantitative PCR (qPCR) reaction for each novel sequence. However, this technique has one major drawback: some medically important species’ DNA cannot be amplified by consensus PCR primers, so it only paints a partial picture of the microbiome. Despite these three disadvantages of unbiased high-throughput sequencing compared to the conventional method listed above, this new technique will likely enable a second major breakthrough in microbiome analysis, revealing medically important species whose DNA is not amplified by typically used consensus PCR primers, as is the case for *Pneumocystis jirovecii, Encephalitozoon hellem* and many other species.

Unbiased high-throughput sequencing can be done using either DNA or RNA. Library preparation for DNA is much simpler and faster than for RNA. For clinical applications or to discover novel microbes during an outbreak, this is a key consideration–especially with the introduction of real-time portable sequencers such as Oxford Nanopore’s MinION, whose complete workflow is very short. RNA sequencing was used in the two first unbiased high-throughput sequencing studies which discovered novel viruses in human specimens [2], [3]. It has two major advantages over DNA sequencing: 1) some types of viruses do not contain DNA (such as RNA viruses and retroviral virions), thus would be missed; 2) the protein product can easily be deduced from mRNA and aligned–and since proteins are typically better conserved than nucleotide sequences, protein alignment may reveal more accurate taxonomic information. For example, protein homology to known viruses allowed Palacios et al. [3] to identify unknown mRNA sequences as likely originating from an arenavirus, whereas nucleotide homology gave ambiguous results (see Table 2 of their article); this approach rendered their experiment immune to bacterial contamination and works well for studies focusing on viruses.

It is unclear whether DNA or RNA sequencing will typically yield a higher microbial/human read ratio, and thus be more sensitive: this property is obviously microbial species dependent. Furthermore, the minimum microbial/human read ratio which results in adverse health outcomes (such as acute illness, chronic inflammation or increased cancer risk) is not known, and is very likely species dependent as well. Feng et al. found a viral/human RNA read ratio of 0.0005% and associated this novel infectious agent with Merkel cell carcinoma [2]; Palacios et al. found a viral/human RNA read ratio of 0.0135%, and this novel virus caused febrile illness resulting in the death of four patients [3]. It is not possible to establish a lower bound of microbial/human read ratio at which health outcomes would be unaffected based on currently available data.

In our prostate microbiome study and in the present study, we chose whole metagenome shotgun sequencing and Illumina’s HiSeq platform as the simplest, cheapest (at equal sensitivity) and most reliable unbiased high-throughput sequencing protocol. We used the Leif Microbiome Analyzer in order to minimize costs; without this tool, the bioinformatics cost for this study would have been about fifty times higher, putting it well beyond our means (see Table S2 and S3). Aside from the presence of contaminants described here, this approach performed remarkably well, detecting many unknown DNA sequences which are novel species candidates. However, the qPCR step required to accurately assess the abundance of these microbes in clinical specimens and link them to prostate cancer proved too costly and uncertain for our project. Before proceeding to the very expensive and labor intensive qPCR step, we chose to study publicly available next-generation sequencing runs in order to better understand the scope and impact of common contaminants such as *Bradyrhizobium* species.

*Bradyrhizobium sp. DFCI-1* was first discovered in cord colitis syndrome specimens [5], suggesting that it may be medically important. Another novel *Bradyrhizobium* species has been reported in a blood culture from a patient with a poorly defined illness [18]. Prior to the cord colitis syndrome study, *Bradyrhizobium* species were not believed to infect humans. *Bradyrhizobium* species are known to be common contaminants in industrial ultrapure water systems, as a consequence of their predilection for nitrogen-flushed water [16]. Other microbes reported in ultrapure water systems and found here include genera *Rhizobium/Agrobacterium*, *Sphingomonas*, *Burkholderia*, *Ralstonia*, *Pseudomonas*, *Stenotrophomonas* and *Flavobacterium*. The presence of *Bradyrhizobium* sequences in several Genbank entries of eukaryotic species (Table 1), in sequencing runs from human specimens (Figure 1) and from *Candida albicans* specimens (Table S1) strongly suggests that it is a ubiquitous laboratory contaminant, although the exact source of contamination remains unclear: it could have been inserted during cell culture, DNA extraction, library preparation or sequencing.

## Conclusion

Novel sequences originating from clinical specimens are of great interest, but they and novel laboratory contaminants both appear in the “Low homology” column in Figure 1. It is very difficult to distinguish microbes of interest from novel contaminants by looking at individual reads. The presence of contamination from organisms that have not been fully sequenced and included in NCBI BLAST databases is particularly problematic when considering projects that aim to identify novel microbes present at low prevalence in clinical specimens, as it is possible that this contamination would obscure detection of such microbes. This problem could be addressed either by eliminating the source of contamination in the laboratory or at the reagent supplier, or by fully sequencing the genomes of contaminants known to be present in these sequencing runs, allowing them to be eliminated during data analysis. A UVC dose of 8000 J/m^2^, four times the dose specified by the US EPA for making surface water sources safe for drinking, will introduce a sufficient density of cyclobutane pyrimidine dimers to prevent ∼99.99% of 100 nucleotide long DNA fragments from being PCR-amplified or sequenced. The numbers of contaminant reads encountered in Figure 1 and Table S1 suggests that this level of inactivation may be necessary.

We advocate a directed effort to sequence contaminant genomes, as this method is well suited to detecting *Bradyrhizobium* and other contaminants that may already be present in Genbank entries. Moreover, until an effective strategy is found to eliminate contaminants from sequencing runs, novel sequences detected in such runs should be scrutinized by qPCR analysis or otherwise to ensure they originate from clinical specimens rather than from the laboratory. Comparing the number of novel organism cells per human cell calculated by using the read counts versus by qPCR or in situ hybridization could highlight discrepancies. Contamination notwithstanding, novel microbe identification based on unbiased high-throughput sequencing is very promising in the study of idiopathic disease, especially as sequencing technology continues to improve.

## Supporting Information

Table S1 **The contents of non-aligning reads from 142** ***Candida albicans*** **whole genome sequencing runs.**
(DOC)

Table S2 **Runtime and cost of Leif Microbiome Analyzer for 57 human runs.**
(DOC)

Table S3 **Runtime and cost of Leif Microbiome Analyzer for 142** ***Candida albicans*** **runs.**
(DOC)

Text S1 **Batch file commands to analyze** ***Bradyrhizobium*** **contaminants in NCBI BLAST databases using the Leif Microbiome Analyzer.**
(DOC)

Text S2 **Batch file commands to analyze contaminants in 57 publicly available 1000 Genomes Project runs using the Leif Microbiome Analyzer.**
(DOC)

Text S3 **Batch file commands to analyze contaminants in 142 publicly available** ***Candida albicans*** **runs using the Leif Microbiome Analyzer.**
(DOC)

Text S4 **Example of a specific alignment to** ***Bradyrhizobium sp. DFCI-1*** **from an Illumina HiSeq 2000 run at the Baylor College of Medicine.**
(DOC)

Text S5 **Example of a specific alignment to** ***Bradyrhizobium sp. DFCI-1*** **from an Illumina HiSeq 2000 run at the Sanger Center.**
(DOC)

Text S6 **Example of a very specific alignment to** ***Bradyrhizobium sp. DFCI-1*** **from an Illumina HiSeq 2000 run at the Broad Institute.**
(DOC)

Text S7 **Example of a less specific alignment to** ***Bradyrhizobium sp. DFCI-1*** **from an Illumina HiSeq 2000 run at the Max Planck Institute for Molecular Genetics.**
(DOC)

Spreadsheet S1 **Maximum taxonomic resolution of non-aligning reads from 57 human whole genome sequencing runs.**
(XLS)

Spreadsheet S2 **Maximum taxonomic resolution of non-aligning reads from 142** ***Candida albicans*** **whole genome sequencing runs.**
(XLS)
