# Supplementary material for: Common Contaminants in Next-Generation Sequencing That Hinder Discovery of Low-Abundance Microbes
Source: PLoS One. 2014 May 16;9(5):e97876. doi: 10.1371/journal.pone.0097876 (PMC4023998; doi:10.1371/journal.pone.0097876)
Supplement: Table S1 — The contents of non-aligning reads from 142 Candida albicans whole genome sequencing runs. (DOC) [file pone.0097876.s001.doc]

**Table S1: The contents of non-aligning reads from 142 *Candida albicans* whole genome sequencing runs**. Categories are defined in Methods. Sequencing center acronyms in this table are: the Broad Institute (BI). Most eukaryote contamination is from various fungi, *Plasmodium* and *Mus*. Runs are sorted alphabetically by center, then by SRA number which are assigned successively over time. Units are read pairs.

| Center | Run | All read pairs | | | | | | | | | |
| --- | --- | --- | --- | --- | --- | --- | --- | --- | --- | --- | --- |
|  |  | *Candida*, phage or discarded due to low quality/ duplicate/ low entropy | Contamination candidates | | | | | | | | |
|  |  |  | Known contaminants (high homology - ≥90%) | | | | | | | | Low homology (<90%) |
|  |  |  | Eukar-yote | Prokaryote | | | | | Viral | Dual homo-logy |  |
|  |  |  |  | Ultrapure water system contaminants | | | Entero-bacte-riaceae | Others & dual homo-logy |  |  |  |
|  |  |  |  | *Bradyrhizobium* | | Others & dual homo-logy |  |  |  |  |  |
|  |  |  |  | *DFCI-1* | Others & dual homology |  |  |  |  |  |  |
| BI | SRR317500 | 4277820 | 70 | 0 | 1 | 2 | 16 | 108 | 0 | 589 | 4226 |
| BI | SRR317501 | 3242857 | 445 | 0 | 0 | 39 | 56 | 1627 | 0 | 4345 | 1594 |
| BI | SRR326077 | 4805836 | 397 | 0 | 2 | 24 | 28 | 965 | 0 | 2803 | 2295 |
| BI | SRR326079 | 3105982 | 808 | 1 | 2 | 16 | 26 | 608 | 1 | 1670 | 782 |
| BI | SRR326080 | 5573966 | 576 | 1 | 1 | 12 | 14 | 373 | 0 | 1266 | 2174 |
| BI | SRR326090 | 4486745 | 101 | 0 | 0 | 5 | 8 | 152 | 0 | 932 | 1946 |
| BI | SRR326091 | 5809716 | 306 | 1 | 4 | 26 | 23 | 677 | 0 | 2001 | 2323 |
| BI | SRR326092 | 4710500 | 435 | 1 | 2 | 37 | 25 | 955 | 0 | 2844 | 1999 |
| BI | SRR326095 | 4772634 | 182 | 1 | 1 | 6 | 15 | 157 | 0 | 953 | 4407 |
| BI | SRR326097 | 4990107 | 1844 | 1 | 1 | 2 | 12 | 145 | 0 | 643 | 473 |
| BI | SRR326106 | 4133377 | 160 | 0 | 2 | 9 | 14 | 296 | 0 | 905 | 4260 |
| BI | SRR326111 | 4143771 | 648 | 2 | 2 | 9 | 19 | 259 | 0 | 1104 | 624 |
| BI | SRR326114 | 4657474 | 1300 | 3 | 1 | 13 | 12 | 287 | 0 | 896 | 614 |
| BI | SRR326115 | 4426343 | 39 | 1 | 0 | 2 | 13 | 86 | 0 | 467 | 1618 |
| BI | SRR326118 | 5103545 | 582 | 1 | 1 | 2 | 13 | 135 | 0 | 695 | 398 |
| BI | SRR326121 | 5296256 | 270 | 1 | 2 | 10 | 10 | 410 | 0 | 2879 | 1890 |
| BI | SRR326122 | 5692609 | 70 | 0 | 2 | 4 | 11 | 136 | 0 | 787 | 1989 |
| BI | SRR326124 | 5357488 | 130 | 2 | 1 | 8 | 15 | 277 | 0 | 1050 | 2169 |
| BI | SRR326125 | 4674368 | 71 | 1 | 1 | 5 | 16 | 137 | 0 | 683 | 4391 |
| BI | SRR326126 | 4350515 | 218 | 2 | 1 | 6 | 18 | 157 | 0 | 639 | 1996 |
| BI | SRR326127 | 4210958 | 331 | 1 | 4 | 18 | 19 | 833 | 0 | 2270 | 1598 |
| BI | SRR326129 | 3926346 | 104 | 1 | 2 | 3 | 13 | 185 | 0 | 904 | 1900 |
| BI | SRR326131 | 3183320 | 513 | 4 | 2 | 24 | 23 | 1431 | 0 | 3529 | 3245 |
| BI | SRR326132 | 5140779 | 1182 | 2 | 1 | 4 | 14 | 238 | 0 | 694 | 1081 |
| BI | SRR326142 | 4532080 | 157 | 2 | 2 | 4 | 15 | 191 | 0 | 1429 | 610 |
| BI | SRR326143 | 4867863 | 104 | 0 | 3 | 5 | 12 | 309 | 0 | 860 | 1078 |
| BI | SRR326150 | 4089123 | 258 | 0 | 1 | 16 | 27 | 502 | 0 | 1692 | 1526 |
| BI | SRR326152 | 5042996 | 1282 | 0 | 2 | 16 | 14 | 512 | 0 | 1415 | 618 |
| BI | SRR326153 | 5073634 | 43 | 1 | 0 | 5 | 21 | 127 | 0 | 725 | 1154 |
| BI | SRR326154 | 2253182 | 219 | 1 | 3 | 17 | 36 | 769 | 0 | 2640 | 1969 |
| BI | SRR392813 | 11307380 | 23 | 40 | 31 | 970 | 8 | 300 | 8 | 342 | 1508 |
| BI | SRR392814 | 11306781 | 1448 | 43 | 23 | 722 | 5 | 219 | 5 | 255 | 12239 |
| BI | SRR393514 | 11251440 | 49 | 69 | 53 | 1312 | 9 | 597 | 11 | 398 | 8071 |
| BI | SRR393515 | 12570954 | 252 | 39 | 33 | 1541 | 9 | 554 | 16 | 410 | 3628 |
| BI | SRR393516 | 11231413 | 25 | 25 | 13 | 741 | 9 | 251 | 7 | 326 | 1634 |
| BI | SRR393517 | 10861826 | 1784 | 17 | 11 | 342 | 5 | 125 | 4 | 189 | 3794 |
| BI | SRR393518 | 12194226 | 366 | 55 | 53 | 5347 | 12 | 1389 | 61 | 719 | 7111 |
| BI | SRR393519 | 9417433 | 26 | 15 | 7 | 682 | 6 | 318 | 4 | 383 | 5987 |
| BI | SRR393520 | 12194210 | 5 | 5 | 3 | 395 | 8 | 129 | 9 | 197 | 920 |
| BI | SRR393521 | 11685893 | 4 | 20 | 19 | 220 | 10 | 101 | 1 | 209 | 1836 |
| BI | SRR393522 | 11674670 | 56 | 54 | 46 | 1641 | 3 | 699 | 19 | 458 | 7662 |
| BI | SRR393523 | 10690329 | 9 | 6 | 5 | 204 | 9 | 76 | 2 | 184 | 703 |
| BI | SRR393524 | 10290322 | 6 | 5 | 7 | 175 | 7 | 70 | 2 | 174 | 1746 |
| BI | SRR393525 | 12968541 | 1242 | 19 | 14 | 808 | 8 | 266 | 2 | 219 | 1436 |
| BI | SRR393526 | 9843008 | 11 | 27 | 24 | 342 | 4 | 128 | 3 | 196 | 590 |
| BI | SRR393527 | 10706095 | 357 | 38 | 29 | 526 | 10 | 214 | 4 | 258 | 1771 |
| BI | SRR393528 | 11423469 | 22 | 17 | 17 | 1057 | 8 | 266 | 7 | 222 | 1398 |
| BI | SRR393529 | 14151860 | 84 | 32 | 57 | 2241 | 5 | 630 | 33 | 444 | 3262 |
| BI | SRR393530 | 13727955 | 399 | 38 | 57 | 12004 | 15 | 4318 | 108 | 2116 | 28114 |
| BI | SRR397731 | 12707033 | 18 | 44 | 47 | 1067 | 8 | 338 | 12 | 263 | 5926 |
| BI | SRR530262 | 27954193 | 625 | 0 | 0 | 6 | 134 | 64 | 0 | 2812 | 36242 |
| BI | SRR530263 | 32560729 | 609 | 0 | 2 | 22 | 161 | 117 | 0 | 3363 | 1688 |
| BI | SRR538771 | 13472726 | 14897 | 2 | 31 | 129 | 1 | 143 | 1 | 971 | 3412 |
| BI | SRR538772 | 11493492 | 3588 | 2 | 52 | 141 | 3 | 161 | 1 | 22632 | 2946 |
| BI | SRR538773 | 10556207 | 3505 | 0 | 31 | 88 | 1 | 89 | 4 | 675 | 2831 |
| BI | SRR538774 | 11753129 | 6090 | 1 | 29 | 114 | 2 | 344 | 2 | 966 | 3149 |
| BI | SRR538775 | 13501655 | 5391 | 2 | 51 | 154 | 2 | 175 | 1 | 987 | 14161 |
| BI | SRR538776 | 10473559 | 2871 | 1 | 29 | 79 | 1 | 94 | 1 | 1082 | 2474 |
| BI | SRR538777 | 11456603 | 2982 | 0 | 41 | 109 | 2 | 151 | 1 | 21302 | 2760 |
| BI | SRR538778 | 11696125 | 5852 | 0 | 27 | 92 | 0 | 287 | 0 | 904 | 3101 |
| BI | SRR538779 | 9697733 | 2976 | 0 | 27 | 120 | 2 | 80 | 1 | 452 | 1690 |
| BI | SRR538780 | 9774386 | 2999 | 0 | 37 | 95 | 0 | 106 | 1 | 494 | 1759 |
| BI | SRR538781 | 10584333 | 3557 | 2 | 46 | 219 | 0 | 183 | 2 | 626 | 2251 |
| BI | SRR538782 | 12186332 | 5531 | 1 | 34 | 110 | 4 | 480 | 1 | 1121 | 3246 |
| BI | SRR538783 | 10553656 | 3246 | 1 | 45 | 174 | 0 | 154 | 3 | 584 | 2128 |
| BI | SRR538784 | 12156588 | 5378 | 0 | 22 | 93 | 2 | 436 | 2 | 923 | 3001 |
| BI | SRR538786 | 11375545 | 3951 | 3 | 38 | 25 | 1 | 299 | 2 | 854 | 2441 |
| BI | SRR538787 | 12373747 | 3786 | 1 | 34 | 146 | 4 | 156 | 4 | 614 | 2297 |
| BI | SRR538788 | 11340952 | 3973 | 2 | 37 | 32 | 1 | 318 | 1 | 808 | 2289 |
| BI | SRR540280 | 6860929 | 86 | 5 | 2 | 421 | 18 | 639 | 4 | 1403 | 3607 |
| BI | SRR540281 | 9380142 | 3806 | 0 | 1 | 78 | 25 | 48 | 0 | 539 | 464 |
| BI | SRR540282 | 8891961 | 147 | 7 | 4 | 1106 | 14 | 465 | 13 | 1458 | 4228 |
| BI | SRR540283 | 8714952 | 80 | 3 | 13 | 311 | 35 | 716 | 4 | 1366 | 4646 |
| BI | SRR540284 | 7334619 | 1208 | 0 | 0 | 48 | 32 | 54 | 1 | 503 | 340 |
| BI | SRR540285 | 7513215 | 11 | 3 | 2 | 241 | 24 | 106 | 0 | 638 | 887 |
| BI | SRR543720 | 7525671 | 8 | 0 | 0 | 98 | 50 | 47 | 1 | 1111 | 1231 |
| BI | SRR543721 | 9535075 | 195 | 12 | 13 | 1217 | 28 | 869 | 15 | 1164 | 9776 |
| BI | SRR543722 | 10074132 | 7 | 0 | 0 | 145 | 42 | 92 | 3 | 910 | 1104 |
| BI | SRR543723 | 8574833 | 9 | 2 | 2 | 150 | 11 | 38 | 1 | 404 | 1165 |
| BI | SRR543724 | 10623074 | 2428 | 2 | 2 | 279 | 40 | 173 | 4 | 747 | 896 |
| BI | SRR543725 | 10283289 | 38 | 1 | 4 | 1113 | 36 | 339 | 10 | 690 | 4854 |
| BI | SRR543726 | 8752092 | 1147 | 1 | 0 | 286 | 22 | 73 | 5 | 606 | 343 |
| BI | SRR543727 | 12177698 | 361 | 5 | 10 | 2401 | 22 | 775 | 24 | 2773 | 4268 |
| BI | SRR543728 | 9017157 | 40 | 3 | 0 | 428 | 29 | 334 | 9 | 1758 | 1734 |
| BI | SRR543729 | 9960723 | 2136 | 1 | 1 | 160 | 40 | 65 | 4 | 2008 | 446 |
| BI | SRR543730 | 7974413 | 73 | 4 | 5 | 1389 | 23 | 495 | 17 | 873 | 5354 |
| BI | SRR543731 | 12640725 | 301 | 13 | 10 | 314 | 37 | 1784 | 3 | 1106 | 2529 |
| BI | SRR629743 | 11009803 | 5773 | 0 | 13 | 57 | 1 | 110 | 2 | 731 | 2546 |
| BI | SRR629744 | 10944026 | 5479 | 0 | 16 | 64 | 2 | 103 | 1 | 665 | 2409 |
| BI | SRR630037 | 10252140 | 579 | 1 | 3 | 384 | 33 | 175 | 7 | 1247 | 700 |
| BI | SRR640890 | 9003952 | 1588 | 2 | 520 | 115 | 1 | 373 | 1 | 667 | 3123 |
| BI | SRR640891 | 11032240 | 1979 | 1 | 236 | 286 | 1 | 397 | 5 | 762 | 13758 |
| BI | SRR640892 | 11751539 | 2755 | 0 | 385 | 223 | 0 | 549 | 1 | 1233 | 4497 |
| BI | SRR640893 | 10490762 | 1511 | 3 | 198 | 366 | 0 | 380 | 5 | 683 | 2645 |
| BI | SRR640894 | 11108573 | 2334 | 12 | 1213 | 241 | 1 | 679 | 0 | 31048 | 4174 |
| BI | SRR640895 | 12020820 | 2459 | 6 | 392 | 248 | 1 | 364 | 3 | 913 | 4127 |
| BI | SRR640896 | 9289863 | 2529 | 2 | 409 | 205 | 0 | 527 | 1 | 931 | 4255 |
| BI | SRR640897 | 8910370 | 2237 | 4 | 336 | 216 | 0 | 502 | 1 | 830 | 3410 |
| BI | SRR641726 | 11417968 | 2675 | 14 | 1320 | 280 | 1 | 790 | 2 | 32972 | 4976 |
| BI | SRR641727 | 10663566 | 1735 | 3 | 209 | 483 | 0 | 437 | 5 | 765 | 3329 |
| BI | SRR641728 | 11005504 | 2672 | 2 | 528 | 201 | 0 | 402 | 0 | 1350 | 4350 |
| BI | SRR641729 | 11971101 | 3252 | 3 | 442 | 196 | 1 | 588 | 2 | 1410 | 5370 |
| BI | SRR641730 | 11125195 | 3253 | 3 | 586 | 231 | 0 | 426 | 1 | 1389 | 5065 |
| BI | SRR641731 | 11887665 | 2095 | 4 | 377 | 229 | 0 | 339 | 5 | 831 | 3335 |
| BI | SRR641732 | 11238457 | 2218 | 2 | 286 | 358 | 1 | 431 | 5 | 1005 | 14200 |
| BI | SRR641733 | 11606342 | 3423 | 10 | 440 | 338 | 0 | 515 | 1 | 957 | 3902 |
| BI | SRR641734 | 11480576 | 1860 | 7 | 362 | 312 | 0 | 456 | 2 | 829 | 4441 |
| BI | SRR641735 | 8850028 | 1417 | 9 | 459 | 86 | 0 | 328 | 2 | 568 | 2458 |
| BI | SRR646258 | 12192790 | 2287 | 2 | 444 | 301 | 0 | 570 | 3 | 1151 | 9937 |
| BI | SRR646259 | 11539717 | 2548 | 3 | 388 | 223 | 0 | 515 | 2 | 1102 | 9793 |
| BI | SRR646260 | 11401261 | 2336 | 3 | 347 | 212 | 1 | 441 | 0 | 947 | 8973 |
| BI | SRR647101 | 12582660 | 3069 | 4 | 530 | 277 | 0 | 441 | 2 | 1283 | 5684 |
| BI | SRR647102 | 12332236 | 2089 | 1 | 520 | 366 | 0 | 613 | 2 | 1020 | 5453 |
| BI | SRR647103 | 8549662 | 1914 | 7 | 568 | 113 | 1 | 405 | 1 | 692 | 3310 |
| BI | SRR647104 | 10671308 | 2837 | 12 | 1338 | 312 | 0 | 758 | 1 | 32354 | 5329 |
| BI | SRR647105 | 10109608 | 2445 | 5 | 475 | 285 | 0 | 634 | 4 | 945 | 3922 |
| BI | SRR647106 | 10609751 | 2203 | 4 | 296 | 374 | 1 | 482 | 4 | 879 | 14806 |
| BI | SRR647107 | 10151063 | 1933 | 2 | 220 | 483 | 0 | 433 | 7 | 856 | 3486 |
| BI | SRR647108 | 11165622 | 2275 | 2 | 505 | 217 | 0 | 624 | 1 | 1050 | 3744 |
| BI | SRR647109 | 12041985 | 2983 | 8 | 697 | 283 | 0 | 546 | 0 | 1429 | 4522 |
| BI | SRR845136 | 12501494 | 1577 | 0 | 133 | 26 | 41 | 274 | 0 | 860 | 1651 |
| BI | SRR845137 | 12565644 | 1643 | 2 | 144 | 22 | 40 | 286 | 0 | 950 | 1680 |
| BI | SRR845177 | 18255595 | 3944 | 8 | 187 | 111 | 64 | 410 | 1 | 56992 | 3943 |
| BI | SRR845178 | 18499248 | 4101 | 5 | 233 | 124 | 68 | 453 | 1 | 58377 | 4242 |
| BI | SRR845179 | 13097846 | 2133 | 2 | 88 | 36 | 44 | 140 | 1 | 1000 | 7525 |
| BI | SRR845180 | 13036638 | 7563 | 3 | 91 | 53 | 51 | 157 | 0 | 918 | 1942 |
| BI | SRR845181 | 15225984 | 2145 | 4 | 179 | 10 | 53 | 263 | 0 | 2225 | 2017 |
| BI | SRR845182 | 15391924 | 2381 | 2 | 171 | 13 | 40 | 267 | 0 | 2373 | 2135 |
| BI | SRR845203 | 13627731 | 2009 | 1 | 129 | 18 | 44 | 258 | 0 | 1723 | 1512 |
| BI | SRR845204 | 13780074 | 2248 | 4 | 133 | 14 | 57 | 236 | 0 | 1787 | 1662 |
| BI | SRR845222 | 17946133 | 2962 | 3 | 113 | 75 | 66 | 258 | 0 | 1353 | 2671 |
| BI | SRR845223 | 17642179 | 2647 | 0 | 107 | 67 | 49 | 238 | 0 | 1265 | 2487 |
| BI | SRR845261 | 17127834 | 2690 | 4 | 149 | 40 | 63 | 302 | 2 | 1563 | 2484 |
| BI | SRR845262 | 17396626 | 2837 | 5 | 174 | 36 | 56 | 339 | 0 | 1612 | 2627 |
| BI | SRR845263 | 17929558 | 3031 | 3 | 102 | 47 | 75 | 255 | 0 | 1516 | 2397 |
| BI | SRR845264 | 17631158 | 2892 | 3 | 99 | 40 | 62 | 262 | 2 | 1418 | 2404 |
| BI | SRR845269 | 19139702 | 2637 | 3 | 193 | 93 | 44 | 352 | 1 | 1417 | 4739 |
| BI | SRR845272 | 18865964 | 2498 | 2 | 180 | 100 | 50 | 299 | 1 | 1412 | 4595 |
| BI | SRR845282 | 18368730 | 3014 | 1 | 99 | 80 | 57 | 217 | 1 | 1267 | 13805 |
| BI | SRR845283 | 18669313 | 3028 | 2 | 107 | 66 | 65 | 238 | 0 | 1381 | 13656 |
| BI | SRR848045 | 26059614 | 53592 | 1 | 2 | 49 | 24 | 200 | 254 | 247 | 1253 |
| BI | SRR848046 | 17629813 | 14470 | 1 | 0 | 3 | 36 | 37 | 0 | 328 | 49423 |
