# Supplementary material for: Common Contaminants in Next-Generation Sequencing That Hinder Discovery of Low-Abundance Microbes
Source: PLoS One. 2014 May 16;9(5):e97876. doi: 10.1371/journal.pone.0097876 (PMC4023998; doi:10.1371/journal.pone.0097876)
Supplement: Table S2 — Runtime and cost of Leif Microbiome Analyzer for 57 human runs. (DOC) [file pone.0097876.s002.doc]

**Table S2: Runtime and cost of Leif Microbiome Analyzer for 57 human runs**. Analyzing 57 “1000 Genomes Project” runs on Amazon AWS EC2 hi1.4xlarge instance (octal core, Intel Xeon CPU E5620 @ 2.40 GHz, Windows Server 2012 Base) using the Leif Microbiome Analyzer 0.7.3 and NCBI BLAST databases “nt” “human_genomic” “other_genomic” and “wgs” downloaded April 1^st^ 2014. Cost is calculated based on “On-Demand Instance” pricing on April 1^st^ 2014 (3.10 USD per hour).

| **Step** | **Time (hours)** | **Cost (USD)** | **Comment** |
| --- | --- | --- | --- |
| Download all files | 10 | 31 | Performed by the open source “wget” command, which is not part of the Leif Microbiome Analyzer. |
| Convert SRA to FASTQ | 76 | 236 | Performed by the NCBI program “fastq-dump”, which is not part of the Leif Microbiome Analyzer. |
| Leif library setup | 19 | 59 | Must be run when the NCBI BLAST database is updated. |
| **Setup Subtotal** | **105** | **326** |  |
|  |  |  |  |
| fastq2fx | 8 | 25 |  |
| fx2fx | 1 | 3 |  |
| fxclone | 0 | 0 |  |
| fxgroup | 0 | 0 |  |
| fxsample | 0 | 0 |  |
| **Pre-alignment Subtotal** | **9** | **28** |  |
|  |  |  |  |
| qblast | 16 | 50 | 168318 read pairs aligned against NCBI BLAST databases “nt” “human_genomic” “other_genomic” “wgs”. Aligning with NCBI blastn using the same server would have taken 2356 hours and cost 7305 USD. This is more than one hundred times slower than Leif Microbiome Analyzer’s qblast. |
| **Alignment Subtotal** | **16** | **50** |  |
|  |  |  |  |
| **Total** | **130** | **404** |  |
