# Supplementary material for: Common Contaminants in Next-Generation Sequencing That Hinder Discovery of Low-Abundance Microbes
Source: PLoS One. 2014 May 16;9(5):e97876. doi: 10.1371/journal.pone.0097876 (PMC4023998; doi:10.1371/journal.pone.0097876)
Supplement: Text S1 — Batch file commands to analyze Bradyrhizobium contaminants in NCBI BLAST databases using the Leif Microbiome Analyzer. (DOC) [file pone.0097876.s004.doc]

Text S1: Batch file commands to analyze *Bradyrhizobium* contaminants in NCBI BLAST databases using the Leif Microbiome Analyzer. The fasta2fq command randomly selects 200000 2x100 base sequences from *Bradyrhizobium* genomes found in the NCBI BLAST databases, and aligns these sequences will all eukaryotic sequences in the NCBI BLAST databases. Good matches (>90% homology) which indicate probable contamination are output. The executables required to run this script can be downloaded at [www.shipsphaw.com/leif](http://www.shipsphaw.com/leif) .

echo Started download on %date% at %time%

:: Download files required to convert "gi" to "taxid" from NCBI Taxonomy ftp site (~1 GB).

leif mpdd 0 wget ftp.ncbi.nih.gov/pub/taxonomy/gi_taxid_nucl.dmp.gz

leif mpdd 0 wget ftp.ncbi.nih.gov/pub/taxonomy/taxdump.tar.gz

:: Download NCBI BLAST databases from ftp site (~230GB).

leif mpdd 0 wget ftp.ncbi.nlm.nih.gov/blast/db/FASTA/nt.gz -O blast_nt.fa.gz

leif mpdd 0 wget ftp.ncbi.nlm.nih.gov/blast/db/FASTA/human_genomic.gz -O blast_human_genomic.fa.gz

leif mpdd 0 wget ftp.ncbi.nlm.nih.gov/blast/db/FASTA/other_genomic.gz -O blast_other_genomic.fa.gz

leif mpdd 0 wget ftp.ncbi.nlm.nih.gov/blast/db/FASTA/wgs.gz -O blast_wgs.fa.gz

echo Started Leif Microbiome Analyzer setup on %date% at %time%

:: Build compact binary file "taxid.git" (~0.3 GB).

gzip -f -d gi_taxid_nucl.dmp.gz

gzip -f -d taxdump.tar.gz

tar xvf taxdump.tar nodes.dmp

tar xvf taxdump.tar names.dmp

leif taxid taxid.git nodes.dmp names.dmp gi_taxid_nucl.dmp

:: Check NCBI BLAST files for gi2taxid consitency.

leif mpdd 0 leif facheck blast_wgs.txt blast_wgs.fa.gz taxid.git

leif mpdd 0 leif facheck blast_other_genomic.txt blast_other_genomic.fa.gz taxid.git

leif mpdd 0 leif facheck blast_human_genomic.txt blast_human_genomic.fa.gz taxid.git

leif mpdd 0 leif facheck blast_nt.txt blast_nt.fa.gz taxid.git

leif mpdd 0

echo Started Leif Microbiome Analyzer analysis on %date% at %time%

:: Extract Bradyrhizobium sequences from NCBI BLAST databases.

leif mpdd 0 leif 400 fasta2fa brady_nt.fa e:\fasta\blast\nt.fa.gz taxid.git 374

leif mpdd 0 leif 400 fasta2fa brady_og.fa e:\fasta\blast\other_genomic.fa.gz taxid.git 374

leif mpdd 0 leif 400 fasta2fa brady_wg.fa e:\fasta\blast\wgs.fa.gz taxid.git 374

:: Randomly sample Bradyrhizobium sequences into "Illumina-like" read pairs.

leif fasta2fq brady_0.fq brady_1.fq 200000 0 100 0 brady_??.fa

leif fastq2fx brady_step1.fx "#" brady_0.fq brady_1.fq

leif fxgroup brady_step2.fx brady_step1.fx

leif fxsample 0 1 brady_step3.fx brady_step2.fx

:: Align random Bradyrhizobium read pairs to all eukaryotes in NCBI BLAST databases.

echo word_length = 15; > qblast_settings.txt

echo dust = 1; >> qblast_settings.txt

echo dual_align_pct = 98; >> qblast_settings.txt

echo num_genus = 4; >> qblast_settings.txt

echo num_species = 4; >> qblast_settings.txt

echo num_consensus = 12; >> qblast_settings.txt

echo cat0= 2759; // Eukaryotes >> qblast_settings.txt

leif qblast qblast_settings.txt taxid.git e:\fasta\blast\*.fa.gz brady_step3.fx

:: Output summary of contamination in a CSV file (Excel compatible).

leif qbmajority single 90 50 brady_contam.qb brady_step4.qb brady_step3.qb taxid.git Consensus 1

leif qbconsensus species single 90 0 consensus90_5_step6.csv brady_contam.qb taxid.git

echo Finished on %date% at %time%
