# Supplementary material for: Common Contaminants in Next-Generation Sequencing That Hinder Discovery of Low-Abundance Microbes
Source: PLoS One. 2014 May 16;9(5):e97876. doi: 10.1371/journal.pone.0097876 (PMC4023998; doi:10.1371/journal.pone.0097876)
Supplement: Text S2 — Batch file commands to analyze contaminants in 57 publicly available 1000 Genomes Project runs using the Leif Microbiome Analyzer. (DOC) [file pone.0097876.s005.doc]

Text S2: Batch file commands to analyze contaminants in 57 publicly available 1000 Genomes Project runs using the Leif Microbiome Analyzer. The executables required to run this script can be downloaded at [www.shipsphaw.com/leif](http://www.shipsphaw.com/leif) .

echo Started download on %date% at %time%

:: Download files required to convert "gi" to "taxid" from NCBI Taxonomy ftp site (~1 GB).

leif mpdd 0 wget ftp.ncbi.nih.gov/pub/taxonomy/gi_taxid_nucl.dmp.gz

leif mpdd 0 wget ftp.ncbi.nih.gov/pub/taxonomy/taxdump.tar.gz

:: Download NCBI BLAST databases from ftp site (~230GB).

leif mpdd 0 wget ftp.ncbi.nlm.nih.gov/blast/db/FASTA/nt.gz -O blast_nt.fa.gz

leif mpdd 0 wget ftp.ncbi.nlm.nih.gov/blast/db/FASTA/human_genomic.gz -O blast_human_genomic.fa.gz

leif mpdd 0 wget ftp.ncbi.nlm.nih.gov/blast/db/FASTA/other_genomic.gz -O blast_other_genomic.fa.gz

leif mpdd 0 wget ftp.ncbi.nlm.nih.gov/blast/db/FASTA/wgs.gz -O blast_wgs.fa.gz

:: Download 57 1000 Genomes Project runs from ftp site (~615 GB).

leif mpdd 0 wget ftp-trace.ncbi.nlm.nih.gov/sra/sra-instant/reads/ByRun/sra/ERR/ERR050/ERR050082/ERR050082.sra -O ERR050082.sra

leif mpdd 0 wget ftp-trace.ncbi.nlm.nih.gov/sra/sra-instant/reads/ByRun/sra/ERR/ERR050/ERR050083/ERR050083.sra -O ERR050083.sra

leif mpdd 0 wget ftp-trace.ncbi.nlm.nih.gov/sra/sra-instant/reads/ByRun/sra/ERR/ERR091/ERR091571/ERR091571.sra -O ERR091571.sra

leif mpdd 0 wget ftp-trace.ncbi.nlm.nih.gov/sra/sra-instant/reads/ByRun/sra/ERR/ERR091/ERR091575/ERR091575.sra -O ERR091575.sra

leif mpdd 0 wget ftp-trace.ncbi.nlm.nih.gov/sra/sra-instant/reads/ByRun/sra/ERR/ERR233/ERR233225/ERR233225.sra -O ERR233225.sra

leif mpdd 0 wget ftp-trace.ncbi.nlm.nih.gov/sra/sra-instant/reads/ByRun/sra/ERR/ERR233/ERR233227/ERR233227.sra -O ERR233227.sra

leif mpdd 0 wget ftp-trace.ncbi.nlm.nih.gov/sra/sra-instant/reads/ByRun/sra/ERR/ERR233/ERR233301/ERR233301.sra -O ERR233301.sra

leif mpdd 0 wget ftp-trace.ncbi.nlm.nih.gov/sra/sra-instant/reads/ByRun/sra/ERR/ERR233/ERR233302/ERR233302.sra -O ERR233302.sra

leif mpdd 0 wget ftp-trace.ncbi.nlm.nih.gov/sra/sra-instant/reads/ByRun/sra/ERR/ERR234/ERR234321/ERR234321.sra -O ERR234321.sra

leif mpdd 0 wget ftp-trace.ncbi.nlm.nih.gov/sra/sra-instant/reads/ByRun/sra/ERR/ERR234/ERR234322/ERR234322.sra -O ERR234322.sra

leif mpdd 0 wget ftp-trace.ncbi.nlm.nih.gov/sra/sra-instant/reads/ByRun/sra/ERR/ERR234/ERR234323/ERR234323.sra -O ERR234323.sra

leif mpdd 0 wget ftp-trace.ncbi.nlm.nih.gov/sra/sra-instant/reads/ByRun/sra/ERR/ERR234/ERR234324/ERR234324.sra -O ERR234324.sra

leif mpdd 0 wget ftp-trace.ncbi.nlm.nih.gov/sra/sra-instant/reads/ByRun/sra/ERR/ERR234/ERR234325/ERR234325.sra -O ERR234325.sra

leif mpdd 0 wget ftp-trace.ncbi.nlm.nih.gov/sra/sra-instant/reads/ByRun/sra/ERR/ERR234/ERR234327/ERR234327.sra -O ERR234327.sra

leif mpdd 0 wget ftp-trace.ncbi.nlm.nih.gov/sra/sra-instant/reads/ByRun/sra/ERR/ERR234/ERR234328/ERR234328.sra -O ERR234328.sra

leif mpdd 0 wget ftp-trace.ncbi.nlm.nih.gov/sra/sra-instant/reads/ByRun/sra/ERR/ERR234/ERR234329/ERR234329.sra -O ERR234329.sra

leif mpdd 0 wget ftp-trace.ncbi.nlm.nih.gov/sra/sra-instant/reads/ByRun/sra/ERR/ERR239/ERR239333/ERR239333.sra -O ERR239333.sra

leif mpdd 0 wget ftp-trace.ncbi.nlm.nih.gov/sra/sra-instant/reads/ByRun/sra/ERR/ERR239/ERR239334/ERR239334.sra -O ERR239334.sra

leif mpdd 0 wget ftp-trace.ncbi.nlm.nih.gov/sra/sra-instant/reads/ByRun/sra/SRR/SRR067/SRR067576/SRR067576.sra -O SRR067576.sra

leif mpdd 0 wget ftp-trace.ncbi.nlm.nih.gov/sra/sra-instant/reads/ByRun/sra/SRR/SRR067/SRR067577/SRR067577.sra -O SRR067577.sra

leif mpdd 0 wget ftp-trace.ncbi.nlm.nih.gov/sra/sra-instant/reads/ByRun/sra/SRR/SRR067/SRR067578/SRR067578.sra -O SRR067578.sra

leif mpdd 0 wget ftp-trace.ncbi.nlm.nih.gov/sra/sra-instant/reads/ByRun/sra/SRR/SRR067/SRR067579/SRR067579.sra -O SRR067579.sra

leif mpdd 0 wget ftp-trace.ncbi.nlm.nih.gov/sra/sra-instant/reads/ByRun/sra/SRR/SRR068/SRR068130/SRR068130.sra -O SRR068130.sra

leif mpdd 0 wget ftp-trace.ncbi.nlm.nih.gov/sra/sra-instant/reads/ByRun/sra/SRR/SRR075/SRR075005/SRR075005.sra -O SRR075005.sra

leif mpdd 0 wget ftp-trace.ncbi.nlm.nih.gov/sra/sra-instant/reads/ByRun/sra/SRR/SRR075/SRR075006/SRR075006.sra -O SRR075006.sra

leif mpdd 0 wget ftp-trace.ncbi.nlm.nih.gov/sra/sra-instant/reads/ByRun/sra/SRR/SRR211/SRR211275/SRR211275.sra -O SRR211275.sra

leif mpdd 0 wget ftp-trace.ncbi.nlm.nih.gov/sra/sra-instant/reads/ByRun/sra/SRR/SRR211/SRR211278/SRR211278.sra -O SRR211278.sra

leif mpdd 0 wget ftp-trace.ncbi.nlm.nih.gov/sra/sra-instant/reads/ByRun/sra/SRR/SRR385/SRR385754/SRR385754.sra -O SRR385754.sra

leif mpdd 0 wget ftp-trace.ncbi.nlm.nih.gov/sra/sra-instant/reads/ByRun/sra/SRR/SRR385/SRR385755/SRR385755.sra -O SRR385755.sra

leif mpdd 0 wget ftp-trace.ncbi.nlm.nih.gov/sra/sra-instant/reads/ByRun/sra/SRR/SRR385/SRR385758/SRR385758.sra -O SRR385758.sra

leif mpdd 0 wget ftp-trace.ncbi.nlm.nih.gov/sra/sra-instant/reads/ByRun/sra/SRR/SRR385/SRR385759/SRR385759.sra -O SRR385759.sra

leif mpdd 0 wget ftp-trace.ncbi.nlm.nih.gov/sra/sra-instant/reads/ByRun/sra/SRR/SRR385/SRR385761/SRR385761.sra -O SRR385761.sra

leif mpdd 0 wget ftp-trace.ncbi.nlm.nih.gov/sra/sra-instant/reads/ByRun/sra/SRR/SRR385/SRR385762/SRR385762.sra -O SRR385762.sra

leif mpdd 0 wget ftp-trace.ncbi.nlm.nih.gov/sra/sra-instant/reads/ByRun/sra/SRR/SRR385/SRR385763/SRR385763.sra -O SRR385763.sra

leif mpdd 0 wget ftp-trace.ncbi.nlm.nih.gov/sra/sra-instant/reads/ByRun/sra/SRR/SRR385/SRR385764/SRR385764.sra -O SRR385764.sra

leif mpdd 0 wget ftp-trace.ncbi.nlm.nih.gov/sra/sra-instant/reads/ByRun/sra/SRR/SRR385/SRR385765/SRR385765.sra -O SRR385765.sra

leif mpdd 0 wget ftp-trace.ncbi.nlm.nih.gov/sra/sra-instant/reads/ByRun/sra/SRR/SRR385/SRR385767/SRR385767.sra -O SRR385767.sra

leif mpdd 0 wget ftp-trace.ncbi.nlm.nih.gov/sra/sra-instant/reads/ByRun/sra/SRR/SRR385/SRR385768/SRR385768.sra -O SRR385768.sra

leif mpdd 0 wget ftp-trace.ncbi.nlm.nih.gov/sra/sra-instant/reads/ByRun/sra/SRR/SRR385/SRR385769/SRR385769.sra -O SRR385769.sra

leif mpdd 0 wget ftp-trace.ncbi.nlm.nih.gov/sra/sra-instant/reads/ByRun/sra/SRR/SRR385/SRR385770/SRR385770.sra -O SRR385770.sra

leif mpdd 0 wget ftp-trace.ncbi.nlm.nih.gov/sra/sra-instant/reads/ByRun/sra/SRR/SRR385/SRR385772/SRR385772.sra -O SRR385772.sra

leif mpdd 0 wget ftp-trace.ncbi.nlm.nih.gov/sra/sra-instant/reads/ByRun/sra/SRR/SRR385/SRR385773/SRR385773.sra -O SRR385773.sra

leif mpdd 0 wget ftp-trace.ncbi.nlm.nih.gov/sra/sra-instant/reads/ByRun/sra/SRR/SRR385/SRR385774/SRR385774.sra -O SRR385774.sra

leif mpdd 0 wget ftp-trace.ncbi.nlm.nih.gov/sra/sra-instant/reads/ByRun/sra/SRR/SRR385/SRR385776/SRR385776.sra -O SRR385776.sra

leif mpdd 0 wget ftp-trace.ncbi.nlm.nih.gov/sra/sra-instant/reads/ByRun/sra/SRR/SRR385/SRR385777/SRR385777.sra -O SRR385777.sra

leif mpdd 0 wget ftp-trace.ncbi.nlm.nih.gov/sra/sra-instant/reads/ByRun/sra/SRR/SRR393/SRR393988/SRR393988.sra -O SRR393988.sra

leif mpdd 0 wget ftp-trace.ncbi.nlm.nih.gov/sra/sra-instant/reads/ByRun/sra/SRR/SRR393/SRR393989/SRR393989.sra -O SRR393989.sra

leif mpdd 0 wget ftp-trace.ncbi.nlm.nih.gov/sra/sra-instant/reads/ByRun/sra/SRR/SRR393/SRR393990/SRR393990.sra -O SRR393990.sra

leif mpdd 0 wget ftp-trace.ncbi.nlm.nih.gov/sra/sra-instant/reads/ByRun/sra/SRR/SRR393/SRR393993/SRR393993.sra -O SRR393993.sra

leif mpdd 0 wget ftp-trace.ncbi.nlm.nih.gov/sra/sra-instant/reads/ByRun/sra/SRR/SRR393/SRR393994/SRR393994.sra -O SRR393994.sra

leif mpdd 0 wget ftp-trace.ncbi.nlm.nih.gov/sra/sra-instant/reads/ByRun/sra/SRR/SRR400/SRR400037/SRR400037.sra -O SRR400037.sra

leif mpdd 0 wget ftp-trace.ncbi.nlm.nih.gov/sra/sra-instant/reads/ByRun/sra/SRR/SRR407/SRR407429/SRR407429.sra -O SRR407429.sra

leif mpdd 0 wget ftp-trace.ncbi.nlm.nih.gov/sra/sra-instant/reads/ByRun/sra/SRR/SRR407/SRR407508/SRR407508.sra -O SRR407508.sra

leif mpdd 0 wget ftp-trace.ncbi.nlm.nih.gov/sra/sra-instant/reads/ByRun/sra/SRR/SRR741/SRR741366/SRR741366.sra -O SRR741366.sra

leif mpdd 0 wget ftp-trace.ncbi.nlm.nih.gov/sra/sra-instant/reads/ByRun/sra/SRR/SRR768/SRR768303/SRR768303.sra -O SRR768303.sra

leif mpdd 0 wget ftp-trace.ncbi.nlm.nih.gov/sra/sra-instant/reads/ByRun/sra/SRR/SRR768/SRR768304/SRR768304.sra -O SRR768304.sra

leif mpdd 0 wget ftp-trace.ncbi.nlm.nih.gov/sra/sra-instant/reads/ByRun/sra/SRR/SRR768/SRR768309/SRR768309.sra -O SRR768309.sra

leif mpdd 0

echo Started SRA => FASTQ conversion on %date% at %time%

:: Convert from SRA format to FASTQ format (extracts ~700 GB).

leif mpdd 0 fastq-dump --gzip --split-files ERR050082.sra

leif mpdd 0 fastq-dump --gzip --split-files ERR050083.sra

leif mpdd 0 fastq-dump --gzip --split-files ERR091571.sra

leif mpdd 0 fastq-dump --gzip --split-files ERR091575.sra

leif mpdd 0 fastq-dump --gzip --split-files ERR233225.sra

leif mpdd 0 fastq-dump --gzip --split-files ERR233227.sra

leif mpdd 0 fastq-dump --gzip --split-files ERR233301.sra

leif mpdd 0 fastq-dump --gzip --split-files ERR233302.sra

leif mpdd 0 fastq-dump --gzip --split-files ERR234321.sra

leif mpdd 0 fastq-dump --gzip --split-files ERR234322.sra

leif mpdd 0 fastq-dump --gzip --split-files ERR234323.sra

leif mpdd 0 fastq-dump --gzip --split-files ERR234324.sra

leif mpdd 0 fastq-dump --gzip --split-files ERR234325.sra

leif mpdd 0 fastq-dump --gzip --split-files ERR234327.sra

leif mpdd 0 fastq-dump --gzip --split-files ERR234328.sra

leif mpdd 0 fastq-dump --gzip --split-files ERR234329.sra

leif mpdd 0 fastq-dump --gzip --split-files ERR239333.sra

leif mpdd 0 fastq-dump --gzip --split-files ERR239334.sra

leif mpdd 0 fastq-dump --gzip --split-files SRR067576.sra

leif mpdd 0 fastq-dump --gzip --split-files SRR067577.sra

leif mpdd 0 fastq-dump --gzip --split-files SRR067578.sra

leif mpdd 0 fastq-dump --gzip --split-files SRR067579.sra

leif mpdd 0 fastq-dump --gzip --split-files SRR068130.sra

leif mpdd 0 fastq-dump --gzip --split-files SRR075005.sra

leif mpdd 0 fastq-dump --gzip --split-files SRR075006.sra

leif mpdd 0 fastq-dump --gzip --split-files SRR211275.sra

leif mpdd 0 fastq-dump --gzip --split-files SRR211278.sra

leif mpdd 0 fastq-dump --gzip --split-files SRR385754.sra

leif mpdd 0 fastq-dump --gzip --split-files SRR385755.sra

leif mpdd 0 fastq-dump --gzip --split-files SRR385758.sra

leif mpdd 0 fastq-dump --gzip --split-files SRR385759.sra

leif mpdd 0 fastq-dump --gzip --split-files SRR385761.sra

leif mpdd 0 fastq-dump --gzip --split-files SRR385762.sra

leif mpdd 0 fastq-dump --gzip --split-files SRR385763.sra

leif mpdd 0 fastq-dump --gzip --split-files SRR385764.sra

leif mpdd 0 fastq-dump --gzip --split-files SRR385765.sra

leif mpdd 0 fastq-dump --gzip --split-files SRR385767.sra

leif mpdd 0 fastq-dump --gzip --split-files SRR385768.sra

leif mpdd 0 fastq-dump --gzip --split-files SRR385769.sra

leif mpdd 0 fastq-dump --gzip --split-files SRR385770.sra

leif mpdd 0 fastq-dump --gzip --split-files SRR385772.sra

leif mpdd 0 fastq-dump --gzip --split-files SRR385773.sra

leif mpdd 0 fastq-dump --gzip --split-files SRR385774.sra

leif mpdd 0 fastq-dump --gzip --split-files SRR385776.sra

leif mpdd 0 fastq-dump --gzip --split-files SRR385777.sra

leif mpdd 0 fastq-dump --gzip --split-files SRR393988.sra

leif mpdd 0 fastq-dump --gzip --split-files SRR393989.sra

leif mpdd 0 fastq-dump --gzip --split-files SRR393990.sra

leif mpdd 0 fastq-dump --gzip --split-files SRR393993.sra

leif mpdd 0 fastq-dump --gzip --split-files SRR393994.sra

leif mpdd 0 fastq-dump --gzip --split-files SRR400037.sra

leif mpdd 0 fastq-dump --gzip --split-files SRR407429.sra

leif mpdd 0 fastq-dump --gzip --split-files SRR407508.sra

leif mpdd 0 fastq-dump --gzip --split-files SRR741366.sra

leif mpdd 0 fastq-dump --gzip --split-files SRR768303.sra

leif mpdd 0 fastq-dump --gzip --split-files SRR768304.sra

leif mpdd 0 fastq-dump --gzip --split-files SRR768309.sra

leif mpdd 0

echo Started Leif Microbiome Analyzer setup on %date% at %time%

:: Build compact binary file "taxid.git" (~0.3 GB).

gzip -f -d gi_taxid_nucl.dmp.gz

gzip -f -d taxdump.tar.gz

tar xvf taxdump.tar nodes.dmp

tar xvf taxdump.tar names.dmp

leif taxid taxid.git nodes.dmp names.dmp gi_taxid_nucl.dmp

:: Build filter dictionnary for ebv, phage, human sequences (~50 GB).

leif mpdd 0 leif fasta2fa ebv.fa blast_nt.fa.gz taxid.git 10376

leif mpdd 0 leif fasta2fa phage.fa blast_nt.fa.gz taxid.git 10841

leif mpdd 0 leif fasta2fa human_0.fa blast_nt.fa.gz taxid.git 9606

leif mpdd 0 leif fasta2fa human_1.fa blast_human_genomic.fa.gz taxid.git 9606

leif mpdd 0 leif fasta2fa human_2.fa blast_wgs.fa.gz taxid.git 9606

leif mpdd 0

leif mpdd 0 leif fasta2fd 0x10 human_0.fd human_?.fa

leif mpdd 0 leif fasta2fd 0x11 human_1.fd human_?.fa

leif mpdd 0 leif fasta2fd 0x12 human_2.fd human_?.fa

leif mpdd 0 leif fasta2fd 0x13 human_3.fd human_?.fa

leif mpdd 0 leif fasta2fd 0x14 human_4.fd human_?.fa

leif mpdd 0 leif fasta2fd 0x15 human_5.fd human_?.fa

leif mpdd 0 leif fasta2fd 0x16 human_6.fd human_?.fa

leif mpdd 0 leif fasta2fd 0x17 human_7.fd human_?.fa

leif mpdd 0 leif fasta2fd 0x18 human_8.fd human_?.fa

leif mpdd 0 leif fasta2fd 0x19 human_9.fd human_?.fa

leif mpdd 0 leif fasta2fd 0x1a human_a.fd human_?.fa

leif mpdd 0 leif fasta2fd 0x1b human_b.fd human_?.fa

leif mpdd 0 leif fasta2fd 0x1c human_c.fd human_?.fa

leif mpdd 0 leif fasta2fd 0x1d human_d.fd human_?.fa

leif mpdd 0 leif fasta2fd 0x1e human_e.fd human_?.fa

leif mpdd 0 leif fasta2fd 0x1f human_f.fd human_?.fa

leif mpdd 0

leif fdmerge human.fd human_?.fd

leif fasta2fd phage.fd phage.fa

leif fasta2fd ebv.fd ebv.fa

leif mpdd 0

del human_?.fd

del human_?.fa

del phage.fa

del ebv.fa

:: Check NCBI BLAST files for gi2taxid consitency.

leif mpdd 0 leif facheck blast_wgs.txt blast_wgs.fa.gz taxid.git

leif mpdd 0 leif facheck blast_other_genomic.txt blast_other_genomic.fa.gz taxid.git

leif mpdd 0 leif facheck blast_human_genomic.txt blast_human_genomic.fa.gz taxid.git

leif mpdd 0 leif facheck blast_nt.txt blast_nt.fa.gz taxid.git

leif mpdd 0

echo Started Leif Microbiome Analyzer analysis on %date% at %time%

:: Align to human sequences (and EBV/phage); discard matching read pairs (part 1).

leif mpdd 0 leif fastq2fx ERR050082_step1.fx "%%" ERR050082_1.fastq.gz ERR050082_2.fastq.gz human.fd phage.fd ebv.fd

leif mpdd 0 leif fastq2fx ERR050083_step1.fx "%%" ERR050083_1.fastq.gz ERR050083_2.fastq.gz human.fd phage.fd ebv.fd

leif mpdd 0 leif fastq2fx ERR091571_step1.fx "%%" ERR091571_1.fastq.gz ERR091571_2.fastq.gz human.fd phage.fd ebv.fd

leif mpdd 0 leif fastq2fx ERR091575_step1.fx "%%" ERR091575_1.fastq.gz ERR091575_2.fastq.gz human.fd phage.fd ebv.fd

leif mpdd 0 leif fastq2fx ERR233225_step1.fx "%%" ERR233225_1.fastq.gz ERR233225_2.fastq.gz human.fd phage.fd ebv.fd

leif mpdd 0 leif fastq2fx ERR233227_step1.fx "%%" ERR233227_1.fastq.gz ERR233227_2.fastq.gz human.fd phage.fd ebv.fd

leif mpdd 0 leif fastq2fx ERR233301_step1.fx "%%" ERR233301_1.fastq.gz ERR233301_2.fastq.gz human.fd phage.fd ebv.fd

leif mpdd 0 leif fastq2fx ERR233302_step1.fx "%%" ERR233302_1.fastq.gz ERR233302_2.fastq.gz human.fd phage.fd ebv.fd

leif mpdd 0 leif fastq2fx ERR234321_step1.fx "%%" ERR234321_1.fastq.gz ERR234321_2.fastq.gz human.fd phage.fd ebv.fd

leif mpdd 0 leif fastq2fx ERR234322_step1.fx "%%" ERR234322_1.fastq.gz ERR234322_2.fastq.gz human.fd phage.fd ebv.fd

leif mpdd 0 leif fastq2fx ERR234323_step1.fx "%%" ERR234323_1.fastq.gz ERR234323_2.fastq.gz human.fd phage.fd ebv.fd

leif mpdd 0 leif fastq2fx ERR234324_step1.fx "%%" ERR234324_1.fastq.gz ERR234324_2.fastq.gz human.fd phage.fd ebv.fd

leif mpdd 0 leif fastq2fx ERR234325_step1.fx "%%" ERR234325_1.fastq.gz ERR234325_2.fastq.gz human.fd phage.fd ebv.fd

leif mpdd 0 leif fastq2fx ERR234327_step1.fx "%%" ERR234327_1.fastq.gz ERR234327_2.fastq.gz human.fd phage.fd ebv.fd

leif mpdd 0 leif fastq2fx ERR234328_step1.fx "%%" ERR234328_1.fastq.gz ERR234328_2.fastq.gz human.fd phage.fd ebv.fd

leif mpdd 0 leif fastq2fx ERR234329_step1.fx "%%" ERR234329_1.fastq.gz ERR234329_2.fastq.gz human.fd phage.fd ebv.fd

leif mpdd 0 leif fastq2fx ERR239333_step1.fx "%%" ERR239333_1.fastq.gz ERR239333_2.fastq.gz human.fd phage.fd ebv.fd

leif mpdd 0 leif fastq2fx ERR239334_step1.fx "%%" ERR239334_1.fastq.gz ERR239334_2.fastq.gz human.fd phage.fd ebv.fd

leif mpdd 0 leif fastq2fx SRR067576_step1.fx "%%" SRR067576_1.fastq.gz SRR067576_2.fastq.gz human.fd phage.fd ebv.fd

leif mpdd 0 leif fastq2fx SRR067577_step1.fx "%%" SRR067577_1.fastq.gz SRR067577_2.fastq.gz human.fd phage.fd ebv.fd

leif mpdd 0 leif fastq2fx SRR067578_step1.fx "%%" SRR067578_1.fastq.gz SRR067578_2.fastq.gz human.fd phage.fd ebv.fd

leif mpdd 0 leif fastq2fx SRR067579_step1.fx "%%" SRR067579_1.fastq.gz SRR067579_2.fastq.gz human.fd phage.fd ebv.fd

leif mpdd 0 leif fastq2fx SRR068130_step1.fx "%%" SRR068130_1.fastq.gz SRR068130_2.fastq.gz human.fd phage.fd ebv.fd

leif mpdd 0 leif fastq2fx SRR075005_step1.fx "%%" SRR075005_1.fastq.gz SRR075005_2.fastq.gz human.fd phage.fd ebv.fd

leif mpdd 0 leif fastq2fx SRR075006_step1.fx "%%" SRR075006_1.fastq.gz SRR075006_2.fastq.gz human.fd phage.fd ebv.fd

leif mpdd 0 leif fastq2fx SRR211275_step1.fx "%%" SRR211275_1.fastq.gz SRR211275_2.fastq.gz human.fd phage.fd ebv.fd

leif mpdd 0 leif fastq2fx SRR211278_step1.fx "%%" SRR211278_1.fastq.gz SRR211278_2.fastq.gz human.fd phage.fd ebv.fd

leif mpdd 0 leif fastq2fx SRR385754_step1.fx "%%" SRR385754_1.fastq.gz SRR385754_2.fastq.gz human.fd phage.fd ebv.fd

leif mpdd 0 leif fastq2fx SRR385755_step1.fx "%%" SRR385755_1.fastq.gz SRR385755_2.fastq.gz human.fd phage.fd ebv.fd

leif mpdd 0 leif fastq2fx SRR385758_step1.fx "%%" SRR385758_1.fastq.gz SRR385758_2.fastq.gz human.fd phage.fd ebv.fd

leif mpdd 0 leif fastq2fx SRR385759_step1.fx "%%" SRR385759_1.fastq.gz SRR385759_2.fastq.gz human.fd phage.fd ebv.fd

leif mpdd 0 leif fastq2fx SRR385761_step1.fx "%%" SRR385761_1.fastq.gz SRR385761_2.fastq.gz human.fd phage.fd ebv.fd

leif mpdd 0 leif fastq2fx SRR385762_step1.fx "%%" SRR385762_1.fastq.gz SRR385762_2.fastq.gz human.fd phage.fd ebv.fd

leif mpdd 0 leif fastq2fx SRR385763_step1.fx "%%" SRR385763_1.fastq.gz SRR385763_2.fastq.gz human.fd phage.fd ebv.fd

leif mpdd 0 leif fastq2fx SRR385764_step1.fx "%%" SRR385764_1.fastq.gz SRR385764_2.fastq.gz human.fd phage.fd ebv.fd

leif mpdd 0 leif fastq2fx SRR385765_step1.fx "%%" SRR385765_1.fastq.gz SRR385765_2.fastq.gz human.fd phage.fd ebv.fd

leif mpdd 0 leif fastq2fx SRR385767_step1.fx "%%" SRR385767_1.fastq.gz SRR385767_2.fastq.gz human.fd phage.fd ebv.fd

leif mpdd 0 leif fastq2fx SRR385768_step1.fx "%%" SRR385768_1.fastq.gz SRR385768_2.fastq.gz human.fd phage.fd ebv.fd

leif mpdd 0 leif fastq2fx SRR385769_step1.fx "%%" SRR385769_1.fastq.gz SRR385769_2.fastq.gz human.fd phage.fd ebv.fd

leif mpdd 0 leif fastq2fx SRR385770_step1.fx "%%" SRR385770_1.fastq.gz SRR385770_2.fastq.gz human.fd phage.fd ebv.fd

leif mpdd 0 leif fastq2fx SRR385772_step1.fx "%%" SRR385772_1.fastq.gz SRR385772_2.fastq.gz human.fd phage.fd ebv.fd

leif mpdd 0 leif fastq2fx SRR385773_step1.fx "%%" SRR385773_1.fastq.gz SRR385773_2.fastq.gz human.fd phage.fd ebv.fd

leif mpdd 0 leif fastq2fx SRR385774_step1.fx "%%" SRR385774_1.fastq.gz SRR385774_2.fastq.gz human.fd phage.fd ebv.fd

leif mpdd 0 leif fastq2fx SRR385776_step1.fx "%%" SRR385776_1.fastq.gz SRR385776_2.fastq.gz human.fd phage.fd ebv.fd

leif mpdd 0 leif fastq2fx SRR385777_step1.fx "%%" SRR385777_1.fastq.gz SRR385777_2.fastq.gz human.fd phage.fd ebv.fd

leif mpdd 0 leif fastq2fx SRR393988_step1.fx "%%" SRR393988_1.fastq.gz SRR393988_2.fastq.gz human.fd phage.fd ebv.fd

leif mpdd 0 leif fastq2fx SRR393989_step1.fx "%%" SRR393989_1.fastq.gz SRR393989_2.fastq.gz human.fd phage.fd ebv.fd

leif mpdd 0 leif fastq2fx SRR393990_step1.fx "%%" SRR393990_1.fastq.gz SRR393990_2.fastq.gz human.fd phage.fd ebv.fd

leif mpdd 0 leif fastq2fx SRR393993_step1.fx "%%" SRR393993_1.fastq.gz SRR393993_2.fastq.gz human.fd phage.fd ebv.fd

leif mpdd 0 leif fastq2fx SRR393994_step1.fx "%%" SRR393994_1.fastq.gz SRR393994_2.fastq.gz human.fd phage.fd ebv.fd

leif mpdd 0 leif fastq2fx SRR400037_step1.fx "%%" SRR400037_1.fastq.gz SRR400037_2.fastq.gz human.fd phage.fd ebv.fd

leif mpdd 0 leif fastq2fx SRR407429_step1.fx "%%" SRR407429_1.fastq.gz SRR407429_2.fastq.gz human.fd phage.fd ebv.fd

leif mpdd 0 leif fastq2fx SRR407508_step1.fx "%%" SRR407508_1.fastq.gz SRR407508_2.fastq.gz human.fd phage.fd ebv.fd

leif mpdd 0 leif fastq2fx SRR741366_step1.fx "%%" SRR741366_1.fastq.gz SRR741366_2.fastq.gz human.fd phage.fd ebv.fd

leif mpdd 0 leif fastq2fx SRR768303_step1.fx "%%" SRR768303_1.fastq.gz SRR768303_2.fastq.gz human.fd phage.fd ebv.fd

leif mpdd 0 leif fastq2fx SRR768304_step1.fx "%%" SRR768304_1.fastq.gz SRR768304_2.fastq.gz human.fd phage.fd ebv.fd

leif mpdd 0 leif fastq2fx SRR768309_step1.fx "%%" SRR768309_1.fastq.gz SRR768309_2.fastq.gz human.fd phage.fd ebv.fd

leif mpdd 0

echo %date% %time%

:: Align to human sequences (and EBV/phage); discard matching read pairs (part 2).

leif mpdd 0 leif fx2fx ERR050082_step2.fx ERR050082_step1.fx human.fd phage.fd ebv.fd

leif mpdd 0 leif fx2fx ERR050083_step2.fx ERR050083_step1.fx human.fd phage.fd ebv.fd

leif mpdd 0 leif fx2fx ERR091571_step2.fx ERR091571_step1.fx human.fd phage.fd ebv.fd

leif mpdd 0 leif fx2fx ERR091575_step2.fx ERR091575_step1.fx human.fd phage.fd ebv.fd

leif mpdd 0 leif fx2fx ERR233225_step2.fx ERR233225_step1.fx human.fd phage.fd ebv.fd

leif mpdd 0 leif fx2fx ERR233227_step2.fx ERR233227_step1.fx human.fd phage.fd ebv.fd

leif mpdd 0 leif fx2fx ERR233301_step2.fx ERR233301_step1.fx human.fd phage.fd ebv.fd

leif mpdd 0 leif fx2fx ERR233302_step2.fx ERR233302_step1.fx human.fd phage.fd ebv.fd

leif mpdd 0 leif fx2fx ERR234321_step2.fx ERR234321_step1.fx human.fd phage.fd ebv.fd

leif mpdd 0 leif fx2fx ERR234322_step2.fx ERR234322_step1.fx human.fd phage.fd ebv.fd

leif mpdd 0 leif fx2fx ERR234323_step2.fx ERR234323_step1.fx human.fd phage.fd ebv.fd

leif mpdd 0 leif fx2fx ERR234324_step2.fx ERR234324_step1.fx human.fd phage.fd ebv.fd

leif mpdd 0 leif fx2fx ERR234325_step2.fx ERR234325_step1.fx human.fd phage.fd ebv.fd

leif mpdd 0 leif fx2fx ERR234327_step2.fx ERR234327_step1.fx human.fd phage.fd ebv.fd

leif mpdd 0 leif fx2fx ERR234328_step2.fx ERR234328_step1.fx human.fd phage.fd ebv.fd

leif mpdd 0 leif fx2fx ERR234329_step2.fx ERR234329_step1.fx human.fd phage.fd ebv.fd

leif mpdd 0 leif fx2fx ERR239333_step2.fx ERR239333_step1.fx human.fd phage.fd ebv.fd

leif mpdd 0 leif fx2fx ERR239334_step2.fx ERR239334_step1.fx human.fd phage.fd ebv.fd

leif mpdd 0 leif fx2fx SRR067576_step2.fx SRR067576_step1.fx human.fd phage.fd ebv.fd

leif mpdd 0 leif fx2fx SRR067577_step2.fx SRR067577_step1.fx human.fd phage.fd ebv.fd

leif mpdd 0 leif fx2fx SRR067578_step2.fx SRR067578_step1.fx human.fd phage.fd ebv.fd

leif mpdd 0 leif fx2fx SRR067579_step2.fx SRR067579_step1.fx human.fd phage.fd ebv.fd

leif mpdd 0 leif fx2fx SRR068130_step2.fx SRR068130_step1.fx human.fd phage.fd ebv.fd

leif mpdd 0 leif fx2fx SRR075005_step2.fx SRR075005_step1.fx human.fd phage.fd ebv.fd

leif mpdd 0 leif fx2fx SRR075006_step2.fx SRR075006_step1.fx human.fd phage.fd ebv.fd

leif mpdd 0 leif fx2fx SRR211275_step2.fx SRR211275_step1.fx human.fd phage.fd ebv.fd

leif mpdd 0 leif fx2fx SRR211278_step2.fx SRR211278_step1.fx human.fd phage.fd ebv.fd

leif mpdd 0 leif fx2fx SRR385754_step2.fx SRR385754_step1.fx human.fd phage.fd ebv.fd

leif mpdd 0 leif fx2fx SRR385755_step2.fx SRR385755_step1.fx human.fd phage.fd ebv.fd

leif mpdd 0 leif fx2fx SRR385758_step2.fx SRR385758_step1.fx human.fd phage.fd ebv.fd

leif mpdd 0 leif fx2fx SRR385759_step2.fx SRR385759_step1.fx human.fd phage.fd ebv.fd

leif mpdd 0 leif fx2fx SRR385761_step2.fx SRR385761_step1.fx human.fd phage.fd ebv.fd

leif mpdd 0 leif fx2fx SRR385762_step2.fx SRR385762_step1.fx human.fd phage.fd ebv.fd

leif mpdd 0 leif fx2fx SRR385763_step2.fx SRR385763_step1.fx human.fd phage.fd ebv.fd

leif mpdd 0 leif fx2fx SRR385764_step2.fx SRR385764_step1.fx human.fd phage.fd ebv.fd

leif mpdd 0 leif fx2fx SRR385765_step2.fx SRR385765_step1.fx human.fd phage.fd ebv.fd

leif mpdd 0 leif fx2fx SRR385767_step2.fx SRR385767_step1.fx human.fd phage.fd ebv.fd

leif mpdd 0 leif fx2fx SRR385768_step2.fx SRR385768_step1.fx human.fd phage.fd ebv.fd

leif mpdd 0 leif fx2fx SRR385769_step2.fx SRR385769_step1.fx human.fd phage.fd ebv.fd

leif mpdd 0 leif fx2fx SRR385770_step2.fx SRR385770_step1.fx human.fd phage.fd ebv.fd

leif mpdd 0 leif fx2fx SRR385772_step2.fx SRR385772_step1.fx human.fd phage.fd ebv.fd

leif mpdd 0 leif fx2fx SRR385773_step2.fx SRR385773_step1.fx human.fd phage.fd ebv.fd

leif mpdd 0 leif fx2fx SRR385774_step2.fx SRR385774_step1.fx human.fd phage.fd ebv.fd

leif mpdd 0 leif fx2fx SRR385776_step2.fx SRR385776_step1.fx human.fd phage.fd ebv.fd

leif mpdd 0 leif fx2fx SRR385777_step2.fx SRR385777_step1.fx human.fd phage.fd ebv.fd

leif mpdd 0 leif fx2fx SRR393988_step2.fx SRR393988_step1.fx human.fd phage.fd ebv.fd

leif mpdd 0 leif fx2fx SRR393989_step2.fx SRR393989_step1.fx human.fd phage.fd ebv.fd

leif mpdd 0 leif fx2fx SRR393990_step2.fx SRR393990_step1.fx human.fd phage.fd ebv.fd

leif mpdd 0 leif fx2fx SRR393993_step2.fx SRR393993_step1.fx human.fd phage.fd ebv.fd

leif mpdd 0 leif fx2fx SRR393994_step2.fx SRR393994_step1.fx human.fd phage.fd ebv.fd

leif mpdd 0 leif fx2fx SRR400037_step2.fx SRR400037_step1.fx human.fd phage.fd ebv.fd

leif mpdd 0 leif fx2fx SRR407429_step2.fx SRR407429_step1.fx human.fd phage.fd ebv.fd

leif mpdd 0 leif fx2fx SRR407508_step2.fx SRR407508_step1.fx human.fd phage.fd ebv.fd

leif mpdd 0 leif fx2fx SRR741366_step2.fx SRR741366_step1.fx human.fd phage.fd ebv.fd

leif mpdd 0 leif fx2fx SRR768303_step2.fx SRR768303_step1.fx human.fd phage.fd ebv.fd

leif mpdd 0 leif fx2fx SRR768304_step2.fx SRR768304_step1.fx human.fd phage.fd ebv.fd

leif mpdd 0 leif fx2fx SRR768309_step2.fx SRR768309_step1.fx human.fd phage.fd ebv.fd

leif mpdd 0

echo %date% %time%

:: Eliminate duplicate/clonal read pairs.

leif mpdd 0 leif fxclone 3 5 60 ERR050082_step3.fx ERR050082_clonegrp.fx ERR050082_step2.fx

leif mpdd 0 leif fxclone 3 5 60 ERR050083_step3.fx ERR050083_clonegrp.fx ERR050083_step2.fx

leif mpdd 0 leif fxclone 3 5 60 ERR091571_step3.fx ERR091571_clonegrp.fx ERR091571_step2.fx

leif mpdd 0 leif fxclone 3 5 60 ERR091575_step3.fx ERR091575_clonegrp.fx ERR091575_step2.fx

leif mpdd 0 leif fxclone 3 5 60 ERR233225_step3.fx ERR233225_clonegrp.fx ERR233225_step2.fx

leif mpdd 0 leif fxclone 3 5 60 ERR233227_step3.fx ERR233227_clonegrp.fx ERR233227_step2.fx

leif mpdd 0 leif fxclone 3 5 60 ERR233301_step3.fx ERR233301_clonegrp.fx ERR233301_step2.fx

leif mpdd 0 leif fxclone 3 5 60 ERR233302_step3.fx ERR233302_clonegrp.fx ERR233302_step2.fx

leif mpdd 0 leif fxclone 3 5 60 ERR234321_step3.fx ERR234321_clonegrp.fx ERR234321_step2.fx

leif mpdd 0 leif fxclone 3 5 60 ERR234322_step3.fx ERR234322_clonegrp.fx ERR234322_step2.fx

leif mpdd 0 leif fxclone 3 5 60 ERR234323_step3.fx ERR234323_clonegrp.fx ERR234323_step2.fx

leif mpdd 0 leif fxclone 3 5 60 ERR234324_step3.fx ERR234324_clonegrp.fx ERR234324_step2.fx

leif mpdd 0 leif fxclone 3 5 60 ERR234325_step3.fx ERR234325_clonegrp.fx ERR234325_step2.fx

leif mpdd 0 leif fxclone 3 5 60 ERR234327_step3.fx ERR234327_clonegrp.fx ERR234327_step2.fx

leif mpdd 0 leif fxclone 3 5 60 ERR234328_step3.fx ERR234328_clonegrp.fx ERR234328_step2.fx

leif mpdd 0 leif fxclone 3 5 60 ERR234329_step3.fx ERR234329_clonegrp.fx ERR234329_step2.fx

leif mpdd 0 leif fxclone 3 5 60 ERR239333_step3.fx ERR239333_clonegrp.fx ERR239333_step2.fx

leif mpdd 0 leif fxclone 3 5 60 ERR239334_step3.fx ERR239334_clonegrp.fx ERR239334_step2.fx

leif mpdd 0 leif fxclone 3 5 60 SRR067576_step3.fx SRR067576_clonegrp.fx SRR067576_step2.fx

leif mpdd 0 leif fxclone 3 5 60 SRR067577_step3.fx SRR067577_clonegrp.fx SRR067577_step2.fx

leif mpdd 0 leif fxclone 3 5 60 SRR067578_step3.fx SRR067578_clonegrp.fx SRR067578_step2.fx

leif mpdd 0 leif fxclone 3 5 60 SRR067579_step3.fx SRR067579_clonegrp.fx SRR067579_step2.fx

leif mpdd 0 leif fxclone 3 5 60 SRR068130_step3.fx SRR068130_clonegrp.fx SRR068130_step2.fx

leif mpdd 0 leif fxclone 3 5 60 SRR075005_step3.fx SRR075005_clonegrp.fx SRR075005_step2.fx

leif mpdd 0 leif fxclone 3 5 60 SRR075006_step3.fx SRR075006_clonegrp.fx SRR075006_step2.fx

leif mpdd 0 leif fxclone 3 5 60 SRR211275_step3.fx SRR211275_clonegrp.fx SRR211275_step2.fx

leif mpdd 0 leif fxclone 3 5 60 SRR211278_step3.fx SRR211278_clonegrp.fx SRR211278_step2.fx

leif mpdd 0 leif fxclone 3 5 60 SRR385754_step3.fx SRR385754_clonegrp.fx SRR385754_step2.fx

leif mpdd 0 leif fxclone 3 5 60 SRR385755_step3.fx SRR385755_clonegrp.fx SRR385755_step2.fx

leif mpdd 0 leif fxclone 3 5 60 SRR385758_step3.fx SRR385758_clonegrp.fx SRR385758_step2.fx

leif mpdd 0 leif fxclone 3 5 60 SRR385759_step3.fx SRR385759_clonegrp.fx SRR385759_step2.fx

leif mpdd 0 leif fxclone 3 5 60 SRR385761_step3.fx SRR385761_clonegrp.fx SRR385761_step2.fx

leif mpdd 0 leif fxclone 3 5 60 SRR385762_step3.fx SRR385762_clonegrp.fx SRR385762_step2.fx

leif mpdd 0 leif fxclone 3 5 60 SRR385763_step3.fx SRR385763_clonegrp.fx SRR385763_step2.fx

leif mpdd 0 leif fxclone 3 5 60 SRR385764_step3.fx SRR385764_clonegrp.fx SRR385764_step2.fx

leif mpdd 0 leif fxclone 3 5 60 SRR385765_step3.fx SRR385765_clonegrp.fx SRR385765_step2.fx

leif mpdd 0 leif fxclone 3 5 60 SRR385767_step3.fx SRR385767_clonegrp.fx SRR385767_step2.fx

leif mpdd 0 leif fxclone 3 5 60 SRR385768_step3.fx SRR385768_clonegrp.fx SRR385768_step2.fx

leif mpdd 0 leif fxclone 3 5 60 SRR385769_step3.fx SRR385769_clonegrp.fx SRR385769_step2.fx

leif mpdd 0 leif fxclone 3 5 60 SRR385770_step3.fx SRR385770_clonegrp.fx SRR385770_step2.fx

leif mpdd 0 leif fxclone 3 5 60 SRR385772_step3.fx SRR385772_clonegrp.fx SRR385772_step2.fx

leif mpdd 0 leif fxclone 3 5 60 SRR385773_step3.fx SRR385773_clonegrp.fx SRR385773_step2.fx

leif mpdd 0 leif fxclone 3 5 60 SRR385774_step3.fx SRR385774_clonegrp.fx SRR385774_step2.fx

leif mpdd 0 leif fxclone 3 5 60 SRR385776_step3.fx SRR385776_clonegrp.fx SRR385776_step2.fx

leif mpdd 0 leif fxclone 3 5 60 SRR385777_step3.fx SRR385777_clonegrp.fx SRR385777_step2.fx

leif mpdd 0 leif fxclone 3 5 60 SRR393988_step3.fx SRR393988_clonegrp.fx SRR393988_step2.fx

leif mpdd 0 leif fxclone 3 5 60 SRR393989_step3.fx SRR393989_clonegrp.fx SRR393989_step2.fx

leif mpdd 0 leif fxclone 3 5 60 SRR393990_step3.fx SRR393990_clonegrp.fx SRR393990_step2.fx

leif mpdd 0 leif fxclone 3 5 60 SRR393993_step3.fx SRR393993_clonegrp.fx SRR393993_step2.fx

leif mpdd 0 leif fxclone 3 5 60 SRR393994_step3.fx SRR393994_clonegrp.fx SRR393994_step2.fx

leif mpdd 0 leif fxclone 3 5 60 SRR400037_step3.fx SRR400037_clonegrp.fx SRR400037_step2.fx

leif mpdd 0 leif fxclone 3 5 60 SRR407429_step3.fx SRR407429_clonegrp.fx SRR407429_step2.fx

leif mpdd 0 leif fxclone 3 5 60 SRR407508_step3.fx SRR407508_clonegrp.fx SRR407508_step2.fx

leif mpdd 0 leif fxclone 3 5 60 SRR741366_step3.fx SRR741366_clonegrp.fx SRR741366_step2.fx

leif mpdd 0 leif fxclone 3 5 60 SRR768303_step3.fx SRR768303_clonegrp.fx SRR768303_step2.fx

leif mpdd 0 leif fxclone 3 5 60 SRR768304_step3.fx SRR768304_clonegrp.fx SRR768304_step2.fx

leif mpdd 0 leif fxclone 3 5 60 SRR768309_step3.fx SRR768309_clonegrp.fx SRR768309_step2.fx

leif mpdd 0

echo %date% %time%

:: Group overlapping read pairs into "contig-like groups".

leif mpdd 0 leif fxgroup ERR050082_step4.fx ERR050082_step3.fx

leif mpdd 0 leif fxgroup ERR050083_step4.fx ERR050083_step3.fx

leif mpdd 0 leif fxgroup ERR091571_step4.fx ERR091571_step3.fx

leif mpdd 0 leif fxgroup ERR091575_step4.fx ERR091575_step3.fx

leif mpdd 0 leif fxgroup ERR233225_step4.fx ERR233225_step3.fx

leif mpdd 0 leif fxgroup ERR233227_step4.fx ERR233227_step3.fx

leif mpdd 0 leif fxgroup ERR233301_step4.fx ERR233301_step3.fx

leif mpdd 0 leif fxgroup ERR233302_step4.fx ERR233302_step3.fx

leif mpdd 0 leif fxgroup ERR234321_step4.fx ERR234321_step3.fx

leif mpdd 0 leif fxgroup ERR234322_step4.fx ERR234322_step3.fx

leif mpdd 0 leif fxgroup ERR234323_step4.fx ERR234323_step3.fx

leif mpdd 0 leif fxgroup ERR234324_step4.fx ERR234324_step3.fx

leif mpdd 0 leif fxgroup ERR234325_step4.fx ERR234325_step3.fx

leif mpdd 0 leif fxgroup ERR234327_step4.fx ERR234327_step3.fx

leif mpdd 0 leif fxgroup ERR234328_step4.fx ERR234328_step3.fx

leif mpdd 0 leif fxgroup ERR234329_step4.fx ERR234329_step3.fx

leif mpdd 0 leif fxgroup ERR239333_step4.fx ERR239333_step3.fx

leif mpdd 0 leif fxgroup ERR239334_step4.fx ERR239334_step3.fx

leif mpdd 0 leif fxgroup SRR067576_step4.fx SRR067576_step3.fx

leif mpdd 0 leif fxgroup SRR067577_step4.fx SRR067577_step3.fx

leif mpdd 0 leif fxgroup SRR067578_step4.fx SRR067578_step3.fx

leif mpdd 0 leif fxgroup SRR067579_step4.fx SRR067579_step3.fx

leif mpdd 0 leif fxgroup SRR068130_step4.fx SRR068130_step3.fx

leif mpdd 0 leif fxgroup SRR075005_step4.fx SRR075005_step3.fx

leif mpdd 0 leif fxgroup SRR075006_step4.fx SRR075006_step3.fx

leif mpdd 0 leif fxgroup SRR211275_step4.fx SRR211275_step3.fx

leif mpdd 0 leif fxgroup SRR211278_step4.fx SRR211278_step3.fx

leif mpdd 0 leif fxgroup SRR385754_step4.fx SRR385754_step3.fx

leif mpdd 0 leif fxgroup SRR385755_step4.fx SRR385755_step3.fx

leif mpdd 0 leif fxgroup SRR385758_step4.fx SRR385758_step3.fx

leif mpdd 0 leif fxgroup SRR385759_step4.fx SRR385759_step3.fx

leif mpdd 0 leif fxgroup SRR385761_step4.fx SRR385761_step3.fx

leif mpdd 0 leif fxgroup SRR385762_step4.fx SRR385762_step3.fx

leif mpdd 0 leif fxgroup SRR385763_step4.fx SRR385763_step3.fx

leif mpdd 0 leif fxgroup SRR385764_step4.fx SRR385764_step3.fx

leif mpdd 0 leif fxgroup SRR385765_step4.fx SRR385765_step3.fx

leif mpdd 0 leif fxgroup SRR385767_step4.fx SRR385767_step3.fx

leif mpdd 0 leif fxgroup SRR385768_step4.fx SRR385768_step3.fx

leif mpdd 0 leif fxgroup SRR385769_step4.fx SRR385769_step3.fx

leif mpdd 0 leif fxgroup SRR385770_step4.fx SRR385770_step3.fx

leif mpdd 0 leif fxgroup SRR385772_step4.fx SRR385772_step3.fx

leif mpdd 0 leif fxgroup SRR385773_step4.fx SRR385773_step3.fx

leif mpdd 0 leif fxgroup SRR385774_step4.fx SRR385774_step3.fx

leif mpdd 0 leif fxgroup SRR385776_step4.fx SRR385776_step3.fx

leif mpdd 0 leif fxgroup SRR385777_step4.fx SRR385777_step3.fx

leif mpdd 0 leif fxgroup SRR393988_step4.fx SRR393988_step3.fx

leif mpdd 0 leif fxgroup SRR393989_step4.fx SRR393989_step3.fx

leif mpdd 0 leif fxgroup SRR393990_step4.fx SRR393990_step3.fx

leif mpdd 0 leif fxgroup SRR393993_step4.fx SRR393993_step3.fx

leif mpdd 0 leif fxgroup SRR393994_step4.fx SRR393994_step3.fx

leif mpdd 0 leif fxgroup SRR400037_step4.fx SRR400037_step3.fx

leif mpdd 0 leif fxgroup SRR407429_step4.fx SRR407429_step3.fx

leif mpdd 0 leif fxgroup SRR407508_step4.fx SRR407508_step3.fx

leif mpdd 0 leif fxgroup SRR741366_step4.fx SRR741366_step3.fx

leif mpdd 0 leif fxgroup SRR768303_step4.fx SRR768303_step3.fx

leif mpdd 0 leif fxgroup SRR768304_step4.fx SRR768304_step3.fx

leif mpdd 0 leif fxgroup SRR768309_step4.fx SRR768309_step3.fx

leif mpdd 0

echo %date% %time%

:: Sample read pairs from each "contig-like group".

leif mpdd 0 leif fxsample 0 1 ERR050082_step5.fx ERR050082_step4.fx

leif mpdd 0 leif fxsample 0 1 ERR050083_step5.fx ERR050083_step4.fx

leif mpdd 0 leif fxsample 0 1 ERR091571_step5.fx ERR091571_step4.fx

leif mpdd 0 leif fxsample 0 1 ERR091575_step5.fx ERR091575_step4.fx

leif mpdd 0 leif fxsample 0 1 ERR233225_step5.fx ERR233225_step4.fx

leif mpdd 0 leif fxsample 0 1 ERR233227_step5.fx ERR233227_step4.fx

leif mpdd 0 leif fxsample 0 1 ERR233301_step5.fx ERR233301_step4.fx

leif mpdd 0 leif fxsample 0 1 ERR233302_step5.fx ERR233302_step4.fx

leif mpdd 0 leif fxsample 0 1 ERR234321_step5.fx ERR234321_step4.fx

leif mpdd 0 leif fxsample 0 1 ERR234322_step5.fx ERR234322_step4.fx

leif mpdd 0 leif fxsample 0 1 ERR234323_step5.fx ERR234323_step4.fx

leif mpdd 0 leif fxsample 0 1 ERR234324_step5.fx ERR234324_step4.fx

leif mpdd 0 leif fxsample 0 1 ERR234325_step5.fx ERR234325_step4.fx

leif mpdd 0 leif fxsample 0 1 ERR234327_step5.fx ERR234327_step4.fx

leif mpdd 0 leif fxsample 0 1 ERR234328_step5.fx ERR234328_step4.fx

leif mpdd 0 leif fxsample 0 1 ERR234329_step5.fx ERR234329_step4.fx

leif mpdd 0 leif fxsample 0 1 ERR239333_step5.fx ERR239333_step4.fx

leif mpdd 0 leif fxsample 0 1 ERR239334_step5.fx ERR239334_step4.fx

leif mpdd 0 leif fxsample 0 1 SRR067576_step5.fx SRR067576_step4.fx

leif mpdd 0 leif fxsample 0 1 SRR067577_step5.fx SRR067577_step4.fx

leif mpdd 0 leif fxsample 0 1 SRR067578_step5.fx SRR067578_step4.fx

leif mpdd 0 leif fxsample 0 1 SRR067579_step5.fx SRR067579_step4.fx

leif mpdd 0 leif fxsample 0 1 SRR068130_step5.fx SRR068130_step4.fx

leif mpdd 0 leif fxsample 0 1 SRR075005_step5.fx SRR075005_step4.fx

leif mpdd 0 leif fxsample 0 1 SRR075006_step5.fx SRR075006_step4.fx

leif mpdd 0 leif fxsample 0 1 SRR211275_step5.fx SRR211275_step4.fx

leif mpdd 0 leif fxsample 0 1 SRR211278_step5.fx SRR211278_step4.fx

leif mpdd 0 leif fxsample 0 1 SRR385754_step5.fx SRR385754_step4.fx

leif mpdd 0 leif fxsample 0 1 SRR385755_step5.fx SRR385755_step4.fx

leif mpdd 0 leif fxsample 0 1 SRR385758_step5.fx SRR385758_step4.fx

leif mpdd 0 leif fxsample 0 1 SRR385759_step5.fx SRR385759_step4.fx

leif mpdd 0 leif fxsample 0 1 SRR385761_step5.fx SRR385761_step4.fx

leif mpdd 0 leif fxsample 0 1 SRR385762_step5.fx SRR385762_step4.fx

leif mpdd 0 leif fxsample 0 1 SRR385763_step5.fx SRR385763_step4.fx

leif mpdd 0 leif fxsample 0 1 SRR385764_step5.fx SRR385764_step4.fx

leif mpdd 0 leif fxsample 0 1 SRR385765_step5.fx SRR385765_step4.fx

leif mpdd 0 leif fxsample 0 1 SRR385767_step5.fx SRR385767_step4.fx

leif mpdd 0 leif fxsample 0 1 SRR385768_step5.fx SRR385768_step4.fx

leif mpdd 0 leif fxsample 0 1 SRR385769_step5.fx SRR385769_step4.fx

leif mpdd 0 leif fxsample 0 1 SRR385770_step5.fx SRR385770_step4.fx

leif mpdd 0 leif fxsample 0 1 SRR385772_step5.fx SRR385772_step4.fx

leif mpdd 0 leif fxsample 0 1 SRR385773_step5.fx SRR385773_step4.fx

leif mpdd 0 leif fxsample 0 1 SRR385774_step5.fx SRR385774_step4.fx

leif mpdd 0 leif fxsample 0 1 SRR385776_step5.fx SRR385776_step4.fx

leif mpdd 0 leif fxsample 0 1 SRR385777_step5.fx SRR385777_step4.fx

leif mpdd 0 leif fxsample 0 1 SRR393988_step5.fx SRR393988_step4.fx

leif mpdd 0 leif fxsample 0 1 SRR393989_step5.fx SRR393989_step4.fx

leif mpdd 0 leif fxsample 0 1 SRR393990_step5.fx SRR393990_step4.fx

leif mpdd 0 leif fxsample 0 1 SRR393993_step5.fx SRR393993_step4.fx

leif mpdd 0 leif fxsample 0 1 SRR393994_step5.fx SRR393994_step4.fx

leif mpdd 0 leif fxsample 0 1 SRR400037_step5.fx SRR400037_step4.fx

leif mpdd 0 leif fxsample 0 1 SRR407429_step5.fx SRR407429_step4.fx

leif mpdd 0 leif fxsample 0 1 SRR407508_step5.fx SRR407508_step4.fx

leif mpdd 0 leif fxsample 0 1 SRR741366_step5.fx SRR741366_step4.fx

leif mpdd 0 leif fxsample 0 1 SRR768303_step5.fx SRR768303_step4.fx

leif mpdd 0 leif fxsample 0 1 SRR768304_step5.fx SRR768304_step4.fx

leif mpdd 0 leif fxsample 0 1 SRR768309_step5.fx SRR768309_step4.fx

leif mpdd 0

echo %date% %time%

:: Align to all four large NCBI BLAST databases (nt, human_genomic, other_genomic, wgs)

echo word_length = 15; > qblast_settings.txt

echo dust = 1; >> qblast_settings.txt

echo dual_align_pct = 98; >> qblast_settings.txt

echo num_genus = 4; >> qblast_settings.txt

echo num_species = 4; >> qblast_settings.txt

echo num_consensus = 12; >> qblast_settings.txt

echo score_taxid= 9443, // Primates >> qblast_settings.txt

echo 10376, // EBV >> qblast_settings.txt

echo 10841; // Microviridae (to catch Enterobacteria phage) >> qblast_settings.txt

echo // >> qblast_settings.txt

echo ignore="|AHJH01"; // Exclude Hammondia hammondi contaminated with Bradyrhizobium. >> qblast_settings.txt

echo ignore="|AGTT01"; // Exclude Pantholops hodgsonii contaminated with Bradyrhizobium. >> qblast_settings.txt

echo ignore="|KE11"; // Exclude Pantholops hodgsonii contaminated with Bradyrhizobium. >> qblast_settings.txt

echo ignore="|AUYS01"; // Exclude Melampsora pinitorqua contaminated with Bradyrhizobium. >> qblast_settings.txt

echo ignore="|ABPJ01"; // Exclude Mchenga conophoros contaminated with Bradyrhizobium. >> qblast_settings.txt

echo ignore="|AK276546.1";// Exclude Gryllus bimaculatus contaminated with E coli. >> qblast_settings.txt

echo ignore="|BADR02"; // Exclude Clonorchis sinensis contaminated with E coli. >> qblast_settings.txt

echo ignore="|CBMN01"; // Exclude Hordeum pubiflorum contaminated with Propionibacterium acnes. >> qblast_settings.txt

echo ignore="|AAHY01"; // Exclude Mus musculus contaminated with E coli. >> qblast_settings.txt

echo ignore="|CAJW01"; // Exclude Hordeum vulgare contaminated with E coli. >> qblast_settings.txt

echo ignore="|CAJX01"; // Exclude Hordeum vulgare contaminated with Ralstonia pickettii. >> qblast_settings.txt

echo ignore="|CAWI01"; // Exclude Adineta vaga contaminated with E coli. >> qblast_settings.txt

echo ignore="|NZ_AJHE02";// Retracted. >> qblast_settings.txt

echo ignore="|CACX01"; // Exclude Strongyloides ratti contaminated with E coli. >> qblast_settings.txt

echo ignore="|CH003510.1";// Exclude Homo sapiens contaminated with E coli. >> qblast_settings.txt

echo ignore="|AHIO01"; // Exclude Plutella xylostella contaminated with Salmonella enterica. >> qblast_settings.txt

echo // >> qblast_settings.txt

echo ignore=81077; // Exclude artificial sequences. >> qblast_settings.txt

echo ignore=12908; // Exclude unclassified sequences. >> qblast_settings.txt

echo cat0= others; // Prokaryotes and viruses. >> qblast_settings.txt

echo cat1= 2759; // Eukaryotes >> qblast_settings.txt

leif qblast qblast_settings.txt taxid.git blast_*.fa.gz *_step5.fx

echo %date% %time%

:: Extract reads which align to primate (taxid=9443), EBV (taxid=10376), phage (taxid=10841) to "pep.qb" file.

leif qbmajority single 70 50 ERR050082_pep.qb ERR050082_step6.qb ERR050082_step5.qb taxid.git Taxid 9443 10376 10841

leif qbmajority single 70 50 ERR050083_pep.qb ERR050083_step6.qb ERR050083_step5.qb taxid.git Taxid 9443 10376 10841

leif qbmajority single 70 50 ERR091571_pep.qb ERR091571_step6.qb ERR091571_step5.qb taxid.git Taxid 9443 10376 10841

leif qbmajority single 70 50 ERR091575_pep.qb ERR091575_step6.qb ERR091575_step5.qb taxid.git Taxid 9443 10376 10841

leif qbmajority single 70 50 ERR233225_pep.qb ERR233225_step6.qb ERR233225_step5.qb taxid.git Taxid 9443 10376 10841

leif qbmajority single 70 50 ERR233227_pep.qb ERR233227_step6.qb ERR233227_step5.qb taxid.git Taxid 9443 10376 10841

leif qbmajority single 70 50 ERR233301_pep.qb ERR233301_step6.qb ERR233301_step5.qb taxid.git Taxid 9443 10376 10841

leif qbmajority single 70 50 ERR233302_pep.qb ERR233302_step6.qb ERR233302_step5.qb taxid.git Taxid 9443 10376 10841

leif qbmajority single 70 50 ERR234321_pep.qb ERR234321_step6.qb ERR234321_step5.qb taxid.git Taxid 9443 10376 10841

leif qbmajority single 70 50 ERR234322_pep.qb ERR234322_step6.qb ERR234322_step5.qb taxid.git Taxid 9443 10376 10841

leif qbmajority single 70 50 ERR234323_pep.qb ERR234323_step6.qb ERR234323_step5.qb taxid.git Taxid 9443 10376 10841

leif qbmajority single 70 50 ERR234324_pep.qb ERR234324_step6.qb ERR234324_step5.qb taxid.git Taxid 9443 10376 10841

leif qbmajority single 70 50 ERR234325_pep.qb ERR234325_step6.qb ERR234325_step5.qb taxid.git Taxid 9443 10376 10841

leif qbmajority single 70 50 ERR234327_pep.qb ERR234327_step6.qb ERR234327_step5.qb taxid.git Taxid 9443 10376 10841

leif qbmajority single 70 50 ERR234328_pep.qb ERR234328_step6.qb ERR234328_step5.qb taxid.git Taxid 9443 10376 10841

leif qbmajority single 70 50 ERR234329_pep.qb ERR234329_step6.qb ERR234329_step5.qb taxid.git Taxid 9443 10376 10841

leif qbmajority single 70 50 ERR239333_pep.qb ERR239333_step6.qb ERR239333_step5.qb taxid.git Taxid 9443 10376 10841

leif qbmajority single 70 50 ERR239334_pep.qb ERR239334_step6.qb ERR239334_step5.qb taxid.git Taxid 9443 10376 10841

leif qbmajority single 70 50 SRR067576_pep.qb SRR067576_step6.qb SRR067576_step5.qb taxid.git Taxid 9443 10376 10841

leif qbmajority single 70 50 SRR067577_pep.qb SRR067577_step6.qb SRR067577_step5.qb taxid.git Taxid 9443 10376 10841

leif qbmajority single 70 50 SRR067578_pep.qb SRR067578_step6.qb SRR067578_step5.qb taxid.git Taxid 9443 10376 10841

leif qbmajority single 70 50 SRR067579_pep.qb SRR067579_step6.qb SRR067579_step5.qb taxid.git Taxid 9443 10376 10841

leif qbmajority single 70 50 SRR068130_pep.qb SRR068130_step6.qb SRR068130_step5.qb taxid.git Taxid 9443 10376 10841

leif qbmajority single 70 50 SRR075005_pep.qb SRR075005_step6.qb SRR075005_step5.qb taxid.git Taxid 9443 10376 10841

leif qbmajority single 70 50 SRR075006_pep.qb SRR075006_step6.qb SRR075006_step5.qb taxid.git Taxid 9443 10376 10841

leif qbmajority single 70 50 SRR211275_pep.qb SRR211275_step6.qb SRR211275_step5.qb taxid.git Taxid 9443 10376 10841

leif qbmajority single 70 50 SRR211278_pep.qb SRR211278_step6.qb SRR211278_step5.qb taxid.git Taxid 9443 10376 10841

leif qbmajority single 70 50 SRR385754_pep.qb SRR385754_step6.qb SRR385754_step5.qb taxid.git Taxid 9443 10376 10841

leif qbmajority single 70 50 SRR385755_pep.qb SRR385755_step6.qb SRR385755_step5.qb taxid.git Taxid 9443 10376 10841

leif qbmajority single 70 50 SRR385758_pep.qb SRR385758_step6.qb SRR385758_step5.qb taxid.git Taxid 9443 10376 10841

leif qbmajority single 70 50 SRR385759_pep.qb SRR385759_step6.qb SRR385759_step5.qb taxid.git Taxid 9443 10376 10841

leif qbmajority single 70 50 SRR385761_pep.qb SRR385761_step6.qb SRR385761_step5.qb taxid.git Taxid 9443 10376 10841

leif qbmajority single 70 50 SRR385762_pep.qb SRR385762_step6.qb SRR385762_step5.qb taxid.git Taxid 9443 10376 10841

leif qbmajority single 70 50 SRR385763_pep.qb SRR385763_step6.qb SRR385763_step5.qb taxid.git Taxid 9443 10376 10841

leif qbmajority single 70 50 SRR385764_pep.qb SRR385764_step6.qb SRR385764_step5.qb taxid.git Taxid 9443 10376 10841

leif qbmajority single 70 50 SRR385765_pep.qb SRR385765_step6.qb SRR385765_step5.qb taxid.git Taxid 9443 10376 10841

leif qbmajority single 70 50 SRR385767_pep.qb SRR385767_step6.qb SRR385767_step5.qb taxid.git Taxid 9443 10376 10841

leif qbmajority single 70 50 SRR385768_pep.qb SRR385768_step6.qb SRR385768_step5.qb taxid.git Taxid 9443 10376 10841

leif qbmajority single 70 50 SRR385769_pep.qb SRR385769_step6.qb SRR385769_step5.qb taxid.git Taxid 9443 10376 10841

leif qbmajority single 70 50 SRR385770_pep.qb SRR385770_step6.qb SRR385770_step5.qb taxid.git Taxid 9443 10376 10841

leif qbmajority single 70 50 SRR385772_pep.qb SRR385772_step6.qb SRR385772_step5.qb taxid.git Taxid 9443 10376 10841

leif qbmajority single 70 50 SRR385773_pep.qb SRR385773_step6.qb SRR385773_step5.qb taxid.git Taxid 9443 10376 10841

leif qbmajority single 70 50 SRR385774_pep.qb SRR385774_step6.qb SRR385774_step5.qb taxid.git Taxid 9443 10376 10841

leif qbmajority single 70 50 SRR385776_pep.qb SRR385776_step6.qb SRR385776_step5.qb taxid.git Taxid 9443 10376 10841

leif qbmajority single 70 50 SRR385777_pep.qb SRR385777_step6.qb SRR385777_step5.qb taxid.git Taxid 9443 10376 10841

leif qbmajority single 70 50 SRR393988_pep.qb SRR393988_step6.qb SRR393988_step5.qb taxid.git Taxid 9443 10376 10841

leif qbmajority single 70 50 SRR393989_pep.qb SRR393989_step6.qb SRR393989_step5.qb taxid.git Taxid 9443 10376 10841

leif qbmajority single 70 50 SRR393990_pep.qb SRR393990_step6.qb SRR393990_step5.qb taxid.git Taxid 9443 10376 10841

leif qbmajority single 70 50 SRR393993_pep.qb SRR393993_step6.qb SRR393993_step5.qb taxid.git Taxid 9443 10376 10841

leif qbmajority single 70 50 SRR393994_pep.qb SRR393994_step6.qb SRR393994_step5.qb taxid.git Taxid 9443 10376 10841

leif qbmajority single 70 50 SRR400037_pep.qb SRR400037_step6.qb SRR400037_step5.qb taxid.git Taxid 9443 10376 10841

leif qbmajority single 70 50 SRR407429_pep.qb SRR407429_step6.qb SRR407429_step5.qb taxid.git Taxid 9443 10376 10841

leif qbmajority single 70 50 SRR407508_pep.qb SRR407508_step6.qb SRR407508_step5.qb taxid.git Taxid 9443 10376 10841

leif qbmajority single 70 50 SRR741366_pep.qb SRR741366_step6.qb SRR741366_step5.qb taxid.git Taxid 9443 10376 10841

leif qbmajority single 70 50 SRR768303_pep.qb SRR768303_step6.qb SRR768303_step5.qb taxid.git Taxid 9443 10376 10841

leif qbmajority single 70 50 SRR768304_pep.qb SRR768304_step6.qb SRR768304_step5.qb taxid.git Taxid 9443 10376 10841

leif qbmajority single 70 50 SRR768309_pep.qb SRR768309_step6.qb SRR768309_step5.qb taxid.git Taxid 9443 10376 10841

:: Output summary of contamination in a CSV file (Excel compatible).

leif qbconsensus single 90 5 consensus90_5_step6.csv *_step6.qb taxid.git

echo Finished on %date% at %time%
