# Supplementary material for: Common Contaminants in Next-Generation Sequencing That Hinder Discovery of Low-Abundance Microbes
Source: PLoS One. 2014 May 16;9(5):e97876. doi: 10.1371/journal.pone.0097876 (PMC4023998; doi:10.1371/journal.pone.0097876)
Supplement: Text S3 — Batch file commands to analyze contaminants in 142 publicly available Candida albicans runs using the Leif Microbiome Analyzer. (DOC) [file pone.0097876.s006.doc]

**Text S3: Batch file commands to analyze contaminants in 142 publicly available *Candida albicans* runs using the Leif Microbiome Analyzer.** The executables required to run this script can be downloaded at [**www.shipsphaw.com/leif**](http://www.shipsphaw.com/leif) .

echo Started download on %date% at %time%

:: Download files required to convert "gi" to "taxid" from NCBI Taxonomy ftp site (~1 GB).

leif mpdd 0 wget ftp.ncbi.nih.gov/pub/taxonomy/gi_taxid_nucl.dmp.gz

leif mpdd 0 wget ftp.ncbi.nih.gov/pub/taxonomy/taxdump.tar.gz

:: Download NCBI BLAST databases from ftp site (~230GB).

leif mpdd 0 wget ftp.ncbi.nlm.nih.gov/blast/db/FASTA/nt.gz -O blast_nt.fa.gz

leif mpdd 0 wget ftp.ncbi.nlm.nih.gov/blast/db/FASTA/human_genomic.gz -O blast_human_genomic.fa.gz

leif mpdd 0 wget ftp.ncbi.nlm.nih.gov/blast/db/FASTA/other_genomic.gz -O blast_other_genomic.fa.gz

leif mpdd 0 wget ftp.ncbi.nlm.nih.gov/blast/db/FASTA/wgs.gz -O blast_wgs.fa.gz

:: Download 142 Candida albicans runs from SRA (~200 GB).

leif mpdd 0 wget ftp-trace.ncbi.nlm.nih.gov/sra/sra-instant/reads/ByRun/sra/SRR/SRR538/SRR538787/SRR538787.sra -O SRR538787.sra

leif mpdd 0 wget ftp-trace.ncbi.nlm.nih.gov/sra/sra-instant/reads/ByRun/sra/SRR/SRR538/SRR538782/SRR538782.sra -O SRR538782.sra

leif mpdd 0 wget ftp-trace.ncbi.nlm.nih.gov/sra/sra-instant/reads/ByRun/sra/SRR/SRR538/SRR538784/SRR538784.sra -O SRR538784.sra

leif mpdd 0 wget ftp-trace.ncbi.nlm.nih.gov/sra/sra-instant/reads/ByRun/sra/SRR/SRR538/SRR538788/SRR538788.sra -O SRR538788.sra

leif mpdd 0 wget ftp-trace.ncbi.nlm.nih.gov/sra/sra-instant/reads/ByRun/sra/SRR/SRR538/SRR538786/SRR538786.sra -O SRR538786.sra

leif mpdd 0 wget ftp-trace.ncbi.nlm.nih.gov/sra/sra-instant/reads/ByRun/sra/SRR/SRR538/SRR538774/SRR538774.sra -O SRR538774.sra

leif mpdd 0 wget ftp-trace.ncbi.nlm.nih.gov/sra/sra-instant/reads/ByRun/sra/SRR/SRR538/SRR538778/SRR538778.sra -O SRR538778.sra

leif mpdd 0 wget ftp-trace.ncbi.nlm.nih.gov/sra/sra-instant/reads/ByRun/sra/SRR/SRR538/SRR538781/SRR538781.sra -O SRR538781.sra

leif mpdd 0 wget ftp-trace.ncbi.nlm.nih.gov/sra/sra-instant/reads/ByRun/sra/SRR/SRR538/SRR538783/SRR538783.sra -O SRR538783.sra

leif mpdd 0 wget ftp-trace.ncbi.nlm.nih.gov/sra/sra-instant/reads/ByRun/sra/SRR/SRR393/SRR393530/SRR393530.sra -O SRR393530.sra

leif mpdd 0 wget ftp-trace.ncbi.nlm.nih.gov/sra/sra-instant/reads/ByRun/sra/SRR/SRR543/SRR543721/SRR543721.sra -O SRR543721.sra

leif mpdd 0 wget ftp-trace.ncbi.nlm.nih.gov/sra/sra-instant/reads/ByRun/sra/SRR/SRR543/SRR543723/SRR543723.sra -O SRR543723.sra

leif mpdd 0 wget ftp-trace.ncbi.nlm.nih.gov/sra/sra-instant/reads/ByRun/sra/SRR/SRR393/SRR393524/SRR393524.sra -O SRR393524.sra

leif mpdd 0 wget ftp-trace.ncbi.nlm.nih.gov/sra/sra-instant/reads/ByRun/sra/SRR/SRR392/SRR392813/SRR392813.sra -O SRR392813.sra

leif mpdd 0 wget ftp-trace.ncbi.nlm.nih.gov/sra/sra-instant/reads/ByRun/sra/SRR/SRR630/SRR630037/SRR630037.sra -O SRR630037.sra

leif mpdd 0 wget ftp-trace.ncbi.nlm.nih.gov/sra/sra-instant/reads/ByRun/sra/SRR/SRR393/SRR393526/SRR393526.sra -O SRR393526.sra

leif mpdd 0 wget ftp-trace.ncbi.nlm.nih.gov/sra/sra-instant/reads/ByRun/sra/SRR/SRR540/SRR540285/SRR540285.sra -O SRR540285.sra

leif mpdd 0 wget ftp-trace.ncbi.nlm.nih.gov/sra/sra-instant/reads/ByRun/sra/SRR/SRR540/SRR540283/SRR540283.sra -O SRR540283.sra

leif mpdd 0 wget ftp-trace.ncbi.nlm.nih.gov/sra/sra-instant/reads/ByRun/sra/SRR/SRR393/SRR393518/SRR393518.sra -O SRR393518.sra

leif mpdd 0 wget ftp-trace.ncbi.nlm.nih.gov/sra/sra-instant/reads/ByRun/sra/SRR/SRR543/SRR543729/SRR543729.sra -O SRR543729.sra

leif mpdd 0 wget ftp-trace.ncbi.nlm.nih.gov/sra/sra-instant/reads/ByRun/sra/SRR/SRR393/SRR393525/SRR393525.sra -O SRR393525.sra

leif mpdd 0 wget ftp-trace.ncbi.nlm.nih.gov/sra/sra-instant/reads/ByRun/sra/SRR/SRR540/SRR540281/SRR540281.sra -O SRR540281.sra

leif mpdd 0 wget ftp-trace.ncbi.nlm.nih.gov/sra/sra-instant/reads/ByRun/sra/SRR/SRR393/SRR393517/SRR393517.sra -O SRR393517.sra

leif mpdd 0 wget ftp-trace.ncbi.nlm.nih.gov/sra/sra-instant/reads/ByRun/sra/SRR/SRR393/SRR393527/SRR393527.sra -O SRR393527.sra

leif mpdd 0 wget ftp-trace.ncbi.nlm.nih.gov/sra/sra-instant/reads/ByRun/sra/SRR/SRR543/SRR543731/SRR543731.sra -O SRR543731.sra

leif mpdd 0 wget ftp-trace.ncbi.nlm.nih.gov/sra/sra-instant/reads/ByRun/sra/SRR/SRR392/SRR392814/SRR392814.sra -O SRR392814.sra

leif mpdd 0 wget ftp-trace.ncbi.nlm.nih.gov/sra/sra-instant/reads/ByRun/sra/SRR/SRR393/SRR393514/SRR393514.sra -O SRR393514.sra

leif mpdd 0 wget ftp-trace.ncbi.nlm.nih.gov/sra/sra-instant/reads/ByRun/sra/SRR/SRR543/SRR543727/SRR543727.sra -O SRR543727.sra

leif mpdd 0 wget ftp-trace.ncbi.nlm.nih.gov/sra/sra-instant/reads/ByRun/sra/SRR/SRR393/SRR393515/SRR393515.sra -O SRR393515.sra

leif mpdd 0 wget ftp-trace.ncbi.nlm.nih.gov/sra/sra-instant/reads/ByRun/sra/SRR/SRR540/SRR540280/SRR540280.sra -O SRR540280.sra

leif mpdd 0 wget ftp-trace.ncbi.nlm.nih.gov/sra/sra-instant/reads/ByRun/sra/SRR/SRR543/SRR543726/SRR543726.sra -O SRR543726.sra

leif mpdd 0 wget ftp-trace.ncbi.nlm.nih.gov/sra/sra-instant/reads/ByRun/sra/SRR/SRR393/SRR393520/SRR393520.sra -O SRR393520.sra

leif mpdd 0 wget ftp-trace.ncbi.nlm.nih.gov/sra/sra-instant/reads/ByRun/sra/SRR/SRR393/SRR393521/SRR393521.sra -O SRR393521.sra

leif mpdd 0 wget ftp-trace.ncbi.nlm.nih.gov/sra/sra-instant/reads/ByRun/sra/SRR/SRR543/SRR543720/SRR543720.sra -O SRR543720.sra

leif mpdd 0 wget ftp-trace.ncbi.nlm.nih.gov/sra/sra-instant/reads/ByRun/sra/SRR/SRR540/SRR540284/SRR540284.sra -O SRR540284.sra

leif mpdd 0 wget ftp-trace.ncbi.nlm.nih.gov/sra/sra-instant/reads/ByRun/sra/SRR/SRR393/SRR393523/SRR393523.sra -O SRR393523.sra

leif mpdd 0 wget ftp-trace.ncbi.nlm.nih.gov/sra/sra-instant/reads/ByRun/sra/SRR/SRR543/SRR543722/SRR543722.sra -O SRR543722.sra

leif mpdd 0 wget ftp-trace.ncbi.nlm.nih.gov/sra/sra-instant/reads/ByRun/sra/SRR/SRR393/SRR393528/SRR393528.sra -O SRR393528.sra

leif mpdd 0 wget ftp-trace.ncbi.nlm.nih.gov/sra/sra-instant/reads/ByRun/sra/SRR/SRR543/SRR543725/SRR543725.sra -O SRR543725.sra

leif mpdd 0 wget ftp-trace.ncbi.nlm.nih.gov/sra/sra-instant/reads/ByRun/sra/SRR/SRR397/SRR397731/SRR397731.sra -O SRR397731.sra

leif mpdd 0 wget ftp-trace.ncbi.nlm.nih.gov/sra/sra-instant/reads/ByRun/sra/SRR/SRR393/SRR393516/SRR393516.sra -O SRR393516.sra

leif mpdd 0 wget ftp-trace.ncbi.nlm.nih.gov/sra/sra-instant/reads/ByRun/sra/SRR/SRR543/SRR543728/SRR543728.sra -O SRR543728.sra

leif mpdd 0 wget ftp-trace.ncbi.nlm.nih.gov/sra/sra-instant/reads/ByRun/sra/SRR/SRR540/SRR540282/SRR540282.sra -O SRR540282.sra

leif mpdd 0 wget ftp-trace.ncbi.nlm.nih.gov/sra/sra-instant/reads/ByRun/sra/SRR/SRR393/SRR393519/SRR393519.sra -O SRR393519.sra

leif mpdd 0 wget ftp-trace.ncbi.nlm.nih.gov/sra/sra-instant/reads/ByRun/sra/SRR/SRR848/SRR848045/SRR848045.sra -O SRR848045.sra

leif mpdd 0 wget ftp-trace.ncbi.nlm.nih.gov/sra/sra-instant/reads/ByRun/sra/SRR/SRR848/SRR848046/SRR848046.sra -O SRR848046.sra

leif mpdd 0 wget ftp-trace.ncbi.nlm.nih.gov/sra/sra-instant/reads/ByRun/sra/SRR/SRR543/SRR543724/SRR543724.sra -O SRR543724.sra

leif mpdd 0 wget ftp-trace.ncbi.nlm.nih.gov/sra/sra-instant/reads/ByRun/sra/SRR/SRR393/SRR393529/SRR393529.sra -O SRR393529.sra

leif mpdd 0 wget ftp-trace.ncbi.nlm.nih.gov/sra/sra-instant/reads/ByRun/sra/SRR/SRR393/SRR393522/SRR393522.sra -O SRR393522.sra

leif mpdd 0 wget ftp-trace.ncbi.nlm.nih.gov/sra/sra-instant/reads/ByRun/sra/SRR/SRR543/SRR543730/SRR543730.sra -O SRR543730.sra

leif mpdd 0 wget ftp-trace.ncbi.nlm.nih.gov/sra/sra-instant/reads/ByRun/sra/SRR/SRR317/SRR317500/SRR317500.sra -O SRR317500.sra

leif mpdd 0 wget ftp-trace.ncbi.nlm.nih.gov/sra/sra-instant/reads/ByRun/sra/SRR/SRR326/SRR326122/SRR326122.sra -O SRR326122.sra

leif mpdd 0 wget ftp-trace.ncbi.nlm.nih.gov/sra/sra-instant/reads/ByRun/sra/SRR/SRR317/SRR317501/SRR317501.sra -O SRR317501.sra

leif mpdd 0 wget ftp-trace.ncbi.nlm.nih.gov/sra/sra-instant/reads/ByRun/sra/SRR/SRR326/SRR326077/SRR326077.sra -O SRR326077.sra

leif mpdd 0 wget ftp-trace.ncbi.nlm.nih.gov/sra/sra-instant/reads/ByRun/sra/SRR/SRR326/SRR326090/SRR326090.sra -O SRR326090.sra

leif mpdd 0 wget ftp-trace.ncbi.nlm.nih.gov/sra/sra-instant/reads/ByRun/sra/SRR/SRR326/SRR326091/SRR326091.sra -O SRR326091.sra

leif mpdd 0 wget ftp-trace.ncbi.nlm.nih.gov/sra/sra-instant/reads/ByRun/sra/SRR/SRR326/SRR326092/SRR326092.sra -O SRR326092.sra

leif mpdd 0 wget ftp-trace.ncbi.nlm.nih.gov/sra/sra-instant/reads/ByRun/sra/SRR/SRR326/SRR326095/SRR326095.sra -O SRR326095.sra

leif mpdd 0 wget ftp-trace.ncbi.nlm.nih.gov/sra/sra-instant/reads/ByRun/sra/SRR/SRR326/SRR326097/SRR326097.sra -O SRR326097.sra

leif mpdd 0 wget ftp-trace.ncbi.nlm.nih.gov/sra/sra-instant/reads/ByRun/sra/SRR/SRR326/SRR326114/SRR326114.sra -O SRR326114.sra

leif mpdd 0 wget ftp-trace.ncbi.nlm.nih.gov/sra/sra-instant/reads/ByRun/sra/SRR/SRR326/SRR326118/SRR326118.sra -O SRR326118.sra

leif mpdd 0 wget ftp-trace.ncbi.nlm.nih.gov/sra/sra-instant/reads/ByRun/sra/SRR/SRR326/SRR326121/SRR326121.sra -O SRR326121.sra

leif mpdd 0 wget ftp-trace.ncbi.nlm.nih.gov/sra/sra-instant/reads/ByRun/sra/SRR/SRR326/SRR326125/SRR326125.sra -O SRR326125.sra

leif mpdd 0 wget ftp-trace.ncbi.nlm.nih.gov/sra/sra-instant/reads/ByRun/sra/SRR/SRR326/SRR326129/SRR326129.sra -O SRR326129.sra

leif mpdd 0 wget ftp-trace.ncbi.nlm.nih.gov/sra/sra-instant/reads/ByRun/sra/SRR/SRR326/SRR326132/SRR326132.sra -O SRR326132.sra

leif mpdd 0 wget ftp-trace.ncbi.nlm.nih.gov/sra/sra-instant/reads/ByRun/sra/SRR/SRR326/SRR326142/SRR326142.sra -O SRR326142.sra

leif mpdd 0 wget ftp-trace.ncbi.nlm.nih.gov/sra/sra-instant/reads/ByRun/sra/SRR/SRR326/SRR326143/SRR326143.sra -O SRR326143.sra

leif mpdd 0 wget ftp-trace.ncbi.nlm.nih.gov/sra/sra-instant/reads/ByRun/sra/SRR/SRR326/SRR326150/SRR326150.sra -O SRR326150.sra

leif mpdd 0 wget ftp-trace.ncbi.nlm.nih.gov/sra/sra-instant/reads/ByRun/sra/SRR/SRR326/SRR326152/SRR326152.sra -O SRR326152.sra

leif mpdd 0 wget ftp-trace.ncbi.nlm.nih.gov/sra/sra-instant/reads/ByRun/sra/SRR/SRR326/SRR326153/SRR326153.sra -O SRR326153.sra

leif mpdd 0 wget ftp-trace.ncbi.nlm.nih.gov/sra/sra-instant/reads/ByRun/sra/SRR/SRR326/SRR326154/SRR326154.sra -O SRR326154.sra

leif mpdd 0 wget ftp-trace.ncbi.nlm.nih.gov/sra/sra-instant/reads/ByRun/sra/SRR/SRR326/SRR326079/SRR326079.sra -O SRR326079.sra

leif mpdd 0 wget ftp-trace.ncbi.nlm.nih.gov/sra/sra-instant/reads/ByRun/sra/SRR/SRR326/SRR326080/SRR326080.sra -O SRR326080.sra

leif mpdd 0 wget ftp-trace.ncbi.nlm.nih.gov/sra/sra-instant/reads/ByRun/sra/SRR/SRR326/SRR326106/SRR326106.sra -O SRR326106.sra

leif mpdd 0 wget ftp-trace.ncbi.nlm.nih.gov/sra/sra-instant/reads/ByRun/sra/SRR/SRR326/SRR326111/SRR326111.sra -O SRR326111.sra

leif mpdd 0 wget ftp-trace.ncbi.nlm.nih.gov/sra/sra-instant/reads/ByRun/sra/SRR/SRR326/SRR326115/SRR326115.sra -O SRR326115.sra

leif mpdd 0 wget ftp-trace.ncbi.nlm.nih.gov/sra/sra-instant/reads/ByRun/sra/SRR/SRR326/SRR326124/SRR326124.sra -O SRR326124.sra

leif mpdd 0 wget ftp-trace.ncbi.nlm.nih.gov/sra/sra-instant/reads/ByRun/sra/SRR/SRR326/SRR326126/SRR326126.sra -O SRR326126.sra

leif mpdd 0 wget ftp-trace.ncbi.nlm.nih.gov/sra/sra-instant/reads/ByRun/sra/SRR/SRR326/SRR326127/SRR326127.sra -O SRR326127.sra

leif mpdd 0 wget ftp-trace.ncbi.nlm.nih.gov/sra/sra-instant/reads/ByRun/sra/SRR/SRR326/SRR326131/SRR326131.sra -O SRR326131.sra

leif mpdd 0 wget ftp-trace.ncbi.nlm.nih.gov/sra/sra-instant/reads/ByRun/sra/SRR/SRR845/SRR845282/SRR845282.sra -O SRR845282.sra

leif mpdd 0 wget ftp-trace.ncbi.nlm.nih.gov/sra/sra-instant/reads/ByRun/sra/SRR/SRR845/SRR845283/SRR845283.sra -O SRR845283.sra

leif mpdd 0 wget ftp-trace.ncbi.nlm.nih.gov/sra/sra-instant/reads/ByRun/sra/SRR/SRR538/SRR538771/SRR538771.sra -O SRR538771.sra

leif mpdd 0 wget ftp-trace.ncbi.nlm.nih.gov/sra/sra-instant/reads/ByRun/sra/SRR/SRR538/SRR538775/SRR538775.sra -O SRR538775.sra

leif mpdd 0 wget ftp-trace.ncbi.nlm.nih.gov/sra/sra-instant/reads/ByRun/sra/SRR/SRR641/SRR641732/SRR641732.sra -O SRR641732.sra

leif mpdd 0 wget ftp-trace.ncbi.nlm.nih.gov/sra/sra-instant/reads/ByRun/sra/SRR/SRR640/SRR640891/SRR640891.sra -O SRR640891.sra

leif mpdd 0 wget ftp-trace.ncbi.nlm.nih.gov/sra/sra-instant/reads/ByRun/sra/SRR/SRR647/SRR647106/SRR647106.sra -O SRR647106.sra

leif mpdd 0 wget ftp-trace.ncbi.nlm.nih.gov/sra/sra-instant/reads/ByRun/sra/SRR/SRR647/SRR647101/SRR647101.sra -O SRR647101.sra

leif mpdd 0 wget ftp-trace.ncbi.nlm.nih.gov/sra/sra-instant/reads/ByRun/sra/SRR/SRR845/SRR845263/SRR845263.sra -O SRR845263.sra

leif mpdd 0 wget ftp-trace.ncbi.nlm.nih.gov/sra/sra-instant/reads/ByRun/sra/SRR/SRR845/SRR845264/SRR845264.sra -O SRR845264.sra

leif mpdd 0 wget ftp-trace.ncbi.nlm.nih.gov/sra/sra-instant/reads/ByRun/sra/SRR/SRR640/SRR640895/SRR640895.sra -O SRR640895.sra

leif mpdd 0 wget ftp-trace.ncbi.nlm.nih.gov/sra/sra-instant/reads/ByRun/sra/SRR/SRR641/SRR641731/SRR641731.sra -O SRR641731.sra

leif mpdd 0 wget ftp-trace.ncbi.nlm.nih.gov/sra/sra-instant/reads/ByRun/sra/SRR/SRR641/SRR641730/SRR641730.sra -O SRR641730.sra

leif mpdd 0 wget ftp-trace.ncbi.nlm.nih.gov/sra/sra-instant/reads/ByRun/sra/SRR/SRR641/SRR641728/SRR641728.sra -O SRR641728.sra

leif mpdd 0 wget ftp-trace.ncbi.nlm.nih.gov/sra/sra-instant/reads/ByRun/sra/SRR/SRR845/SRR845261/SRR845261.sra -O SRR845261.sra

leif mpdd 0 wget ftp-trace.ncbi.nlm.nih.gov/sra/sra-instant/reads/ByRun/sra/SRR/SRR845/SRR845262/SRR845262.sra -O SRR845262.sra

leif mpdd 0 wget ftp-trace.ncbi.nlm.nih.gov/sra/sra-instant/reads/ByRun/sra/SRR/SRR647/SRR647109/SRR647109.sra -O SRR647109.sra

leif mpdd 0 wget ftp-trace.ncbi.nlm.nih.gov/sra/sra-instant/reads/ByRun/sra/SRR/SRR647/SRR647103/SRR647103.sra -O SRR647103.sra

leif mpdd 0 wget ftp-trace.ncbi.nlm.nih.gov/sra/sra-instant/reads/ByRun/sra/SRR/SRR845/SRR845182/SRR845182.sra -O SRR845182.sra

leif mpdd 0 wget ftp-trace.ncbi.nlm.nih.gov/sra/sra-instant/reads/ByRun/sra/SRR/SRR640/SRR640890/SRR640890.sra -O SRR640890.sra

leif mpdd 0 wget ftp-trace.ncbi.nlm.nih.gov/sra/sra-instant/reads/ByRun/sra/SRR/SRR641/SRR641735/SRR641735.sra -O SRR641735.sra

leif mpdd 0 wget ftp-trace.ncbi.nlm.nih.gov/sra/sra-instant/reads/ByRun/sra/SRR/SRR845/SRR845181/SRR845181.sra -O SRR845181.sra

leif mpdd 0 wget ftp-trace.ncbi.nlm.nih.gov/sra/sra-instant/reads/ByRun/sra/SRR/SRR845/SRR845137/SRR845137.sra -O SRR845137.sra

leif mpdd 0 wget ftp-trace.ncbi.nlm.nih.gov/sra/sra-instant/reads/ByRun/sra/SRR/SRR845/SRR845136/SRR845136.sra -O SRR845136.sra

leif mpdd 0 wget ftp-trace.ncbi.nlm.nih.gov/sra/sra-instant/reads/ByRun/sra/SRR/SRR641/SRR641729/SRR641729.sra -O SRR641729.sra

leif mpdd 0 wget ftp-trace.ncbi.nlm.nih.gov/sra/sra-instant/reads/ByRun/sra/SRR/SRR640/SRR640892/SRR640892.sra -O SRR640892.sra

leif mpdd 0 wget ftp-trace.ncbi.nlm.nih.gov/sra/sra-instant/reads/ByRun/sra/SRR/SRR647/SRR647108/SRR647108.sra -O SRR647108.sra

leif mpdd 0 wget ftp-trace.ncbi.nlm.nih.gov/sra/sra-instant/reads/ByRun/sra/SRR/SRR845/SRR845179/SRR845179.sra -O SRR845179.sra

leif mpdd 0 wget ftp-trace.ncbi.nlm.nih.gov/sra/sra-instant/reads/ByRun/sra/SRR/SRR845/SRR845180/SRR845180.sra -O SRR845180.sra

leif mpdd 0 wget ftp-trace.ncbi.nlm.nih.gov/sra/sra-instant/reads/ByRun/sra/SRR/SRR629/SRR629744/SRR629744.sra -O SRR629744.sra

leif mpdd 0 wget ftp-trace.ncbi.nlm.nih.gov/sra/sra-instant/reads/ByRun/sra/SRR/SRR629/SRR629743/SRR629743.sra -O SRR629743.sra

leif mpdd 0 wget ftp-trace.ncbi.nlm.nih.gov/sra/sra-instant/reads/ByRun/sra/SRR/SRR646/SRR646259/SRR646259.sra -O SRR646259.sra

leif mpdd 0 wget ftp-trace.ncbi.nlm.nih.gov/sra/sra-instant/reads/ByRun/sra/SRR/SRR646/SRR646260/SRR646260.sra -O SRR646260.sra

leif mpdd 0 wget ftp-trace.ncbi.nlm.nih.gov/sra/sra-instant/reads/ByRun/sra/SRR/SRR646/SRR646258/SRR646258.sra -O SRR646258.sra

leif mpdd 0 wget ftp-trace.ncbi.nlm.nih.gov/sra/sra-instant/reads/ByRun/sra/SRR/SRR640/SRR640897/SRR640897.sra -O SRR640897.sra

leif mpdd 0 wget ftp-trace.ncbi.nlm.nih.gov/sra/sra-instant/reads/ByRun/sra/SRR/SRR640/SRR640896/SRR640896.sra -O SRR640896.sra

leif mpdd 0 wget ftp-trace.ncbi.nlm.nih.gov/sra/sra-instant/reads/ByRun/sra/SRR/SRR845/SRR845203/SRR845203.sra -O SRR845203.sra

leif mpdd 0 wget ftp-trace.ncbi.nlm.nih.gov/sra/sra-instant/reads/ByRun/sra/SRR/SRR845/SRR845204/SRR845204.sra -O SRR845204.sra

leif mpdd 0 wget ftp-trace.ncbi.nlm.nih.gov/sra/sra-instant/reads/ByRun/sra/SRR/SRR647/SRR647105/SRR647105.sra -O SRR647105.sra

leif mpdd 0 wget ftp-trace.ncbi.nlm.nih.gov/sra/sra-instant/reads/ByRun/sra/SRR/SRR845/SRR845269/SRR845269.sra -O SRR845269.sra

leif mpdd 0 wget ftp-trace.ncbi.nlm.nih.gov/sra/sra-instant/reads/ByRun/sra/SRR/SRR845/SRR845272/SRR845272.sra -O SRR845272.sra

leif mpdd 0 wget ftp-trace.ncbi.nlm.nih.gov/sra/sra-instant/reads/ByRun/sra/SRR/SRR647/SRR647102/SRR647102.sra -O SRR647102.sra

leif mpdd 0 wget ftp-trace.ncbi.nlm.nih.gov/sra/sra-instant/reads/ByRun/sra/SRR/SRR641/SRR641734/SRR641734.sra -O SRR641734.sra

leif mpdd 0 wget ftp-trace.ncbi.nlm.nih.gov/sra/sra-instant/reads/ByRun/sra/SRR/SRR641/SRR641733/SRR641733.sra -O SRR641733.sra

leif mpdd 0 wget ftp-trace.ncbi.nlm.nih.gov/sra/sra-instant/reads/ByRun/sra/SRR/SRR538/SRR538776/SRR538776.sra -O SRR538776.sra

leif mpdd 0 wget ftp-trace.ncbi.nlm.nih.gov/sra/sra-instant/reads/ByRun/sra/SRR/SRR538/SRR538773/SRR538773.sra -O SRR538773.sra

leif mpdd 0 wget ftp-trace.ncbi.nlm.nih.gov/sra/sra-instant/reads/ByRun/sra/SRR/SRR538/SRR538779/SRR538779.sra -O SRR538779.sra

leif mpdd 0 wget ftp-trace.ncbi.nlm.nih.gov/sra/sra-instant/reads/ByRun/sra/SRR/SRR538/SRR538780/SRR538780.sra -O SRR538780.sra

leif mpdd 0 wget ftp-trace.ncbi.nlm.nih.gov/sra/sra-instant/reads/ByRun/sra/SRR/SRR641/SRR641727/SRR641727.sra -O SRR641727.sra

leif mpdd 0 wget ftp-trace.ncbi.nlm.nih.gov/sra/sra-instant/reads/ByRun/sra/SRR/SRR640/SRR640893/SRR640893.sra -O SRR640893.sra

leif mpdd 0 wget ftp-trace.ncbi.nlm.nih.gov/sra/sra-instant/reads/ByRun/sra/SRR/SRR647/SRR647107/SRR647107.sra -O SRR647107.sra

leif mpdd 0 wget ftp-trace.ncbi.nlm.nih.gov/sra/sra-instant/reads/ByRun/sra/SRR/SRR845/SRR845223/SRR845223.sra -O SRR845223.sra

leif mpdd 0 wget ftp-trace.ncbi.nlm.nih.gov/sra/sra-instant/reads/ByRun/sra/SRR/SRR845/SRR845222/SRR845222.sra -O SRR845222.sra

leif mpdd 0 wget ftp-trace.ncbi.nlm.nih.gov/sra/sra-instant/reads/ByRun/sra/SRR/SRR845/SRR845177/SRR845177.sra -O SRR845177.sra

leif mpdd 0 wget ftp-trace.ncbi.nlm.nih.gov/sra/sra-instant/reads/ByRun/sra/SRR/SRR845/SRR845178/SRR845178.sra -O SRR845178.sra

leif mpdd 0 wget ftp-trace.ncbi.nlm.nih.gov/sra/sra-instant/reads/ByRun/sra/SRR/SRR647/SRR647104/SRR647104.sra -O SRR647104.sra

leif mpdd 0 wget ftp-trace.ncbi.nlm.nih.gov/sra/sra-instant/reads/ByRun/sra/SRR/SRR640/SRR640894/SRR640894.sra -O SRR640894.sra

leif mpdd 0 wget ftp-trace.ncbi.nlm.nih.gov/sra/sra-instant/reads/ByRun/sra/SRR/SRR641/SRR641726/SRR641726.sra -O SRR641726.sra

leif mpdd 0 wget ftp-trace.ncbi.nlm.nih.gov/sra/sra-instant/reads/ByRun/sra/SRR/SRR538/SRR538777/SRR538777.sra -O SRR538777.sra

leif mpdd 0 wget ftp-trace.ncbi.nlm.nih.gov/sra/sra-instant/reads/ByRun/sra/SRR/SRR538/SRR538772/SRR538772.sra -O SRR538772.sra

leif mpdd 0 wget ftp-trace.ncbi.nlm.nih.gov/sra/sra-instant/reads/ByRun/sra/SRR/SRR530/SRR530262/SRR530262.sra -O SRR530262.sra

leif mpdd 0 wget ftp-trace.ncbi.nlm.nih.gov/sra/sra-instant/reads/ByRun/sra/SRR/SRR530/SRR530263/SRR530263.sra -O SRR530263.sra

leif mpdd 0

echo Started SRA => FASTQ conversion on %date% at %time%

:: Convert from SRA format to FASTQ format (extracts ~230 GB).

leif mpdd 0 fastq-dump --gzip --split-files SRR538787.sra

leif mpdd 0 fastq-dump --gzip --split-files SRR538782.sra

leif mpdd 0 fastq-dump --gzip --split-files SRR538784.sra

leif mpdd 0 fastq-dump --gzip --split-files SRR538788.sra

leif mpdd 0 fastq-dump --gzip --split-files SRR538786.sra

leif mpdd 0 fastq-dump --gzip --split-files SRR538774.sra

leif mpdd 0 fastq-dump --gzip --split-files SRR538778.sra

leif mpdd 0 fastq-dump --gzip --split-files SRR538781.sra

leif mpdd 0 fastq-dump --gzip --split-files SRR538783.sra

leif mpdd 0 fastq-dump --gzip --split-files SRR393530.sra

leif mpdd 0 fastq-dump --gzip --split-files SRR543721.sra

leif mpdd 0 fastq-dump --gzip --split-files SRR543723.sra

leif mpdd 0 fastq-dump --gzip --split-files SRR393524.sra

leif mpdd 0 fastq-dump --gzip --split-files SRR392813.sra

leif mpdd 0 fastq-dump --gzip --split-files SRR630037.sra

leif mpdd 0 fastq-dump --gzip --split-files SRR393526.sra

leif mpdd 0 fastq-dump --gzip --split-files SRR540285.sra

leif mpdd 0 fastq-dump --gzip --split-files SRR540283.sra

leif mpdd 0 fastq-dump --gzip --split-files SRR393518.sra

leif mpdd 0 fastq-dump --gzip --split-files SRR543729.sra

leif mpdd 0 fastq-dump --gzip --split-files SRR393525.sra

leif mpdd 0 fastq-dump --gzip --split-files SRR540281.sra

leif mpdd 0 fastq-dump --gzip --split-files SRR393517.sra

leif mpdd 0 fastq-dump --gzip --split-files SRR393527.sra

leif mpdd 0 fastq-dump --gzip --split-files SRR543731.sra

leif mpdd 0 fastq-dump --gzip --split-files SRR392814.sra

leif mpdd 0 fastq-dump --gzip --split-files SRR393514.sra

leif mpdd 0 fastq-dump --gzip --split-files SRR543727.sra

leif mpdd 0 fastq-dump --gzip --split-files SRR393515.sra

leif mpdd 0 fastq-dump --gzip --split-files SRR540280.sra

leif mpdd 0 fastq-dump --gzip --split-files SRR543726.sra

leif mpdd 0 fastq-dump --gzip --split-files SRR393520.sra

leif mpdd 0 fastq-dump --gzip --split-files SRR393521.sra

leif mpdd 0 fastq-dump --gzip --split-files SRR543720.sra

leif mpdd 0 fastq-dump --gzip --split-files SRR540284.sra

leif mpdd 0 fastq-dump --gzip --split-files SRR393523.sra

leif mpdd 0 fastq-dump --gzip --split-files SRR543722.sra

leif mpdd 0 fastq-dump --gzip --split-files SRR393528.sra

leif mpdd 0 fastq-dump --gzip --split-files SRR543725.sra

leif mpdd 0 fastq-dump --gzip --split-files SRR397731.sra

leif mpdd 0 fastq-dump --gzip --split-files SRR393516.sra

leif mpdd 0 fastq-dump --gzip --split-files SRR543728.sra

leif mpdd 0 fastq-dump --gzip --split-files SRR540282.sra

leif mpdd 0 fastq-dump --gzip --split-files SRR393519.sra

leif mpdd 0 fastq-dump --gzip --split-files SRR848045.sra

leif mpdd 0 fastq-dump --gzip --split-files SRR848046.sra

leif mpdd 0 fastq-dump --gzip --split-files SRR543724.sra

leif mpdd 0 fastq-dump --gzip --split-files SRR393529.sra

leif mpdd 0 fastq-dump --gzip --split-files SRR393522.sra

leif mpdd 0 fastq-dump --gzip --split-files SRR543730.sra

leif mpdd 0 fastq-dump --gzip --split-files SRR317500.sra

leif mpdd 0 fastq-dump --gzip --split-files SRR326122.sra

leif mpdd 0 fastq-dump --gzip --split-files SRR317501.sra

leif mpdd 0 fastq-dump --gzip --split-files SRR326077.sra

leif mpdd 0 fastq-dump --gzip --split-files SRR326090.sra

leif mpdd 0 fastq-dump --gzip --split-files SRR326091.sra

leif mpdd 0 fastq-dump --gzip --split-files SRR326092.sra

leif mpdd 0 fastq-dump --gzip --split-files SRR326095.sra

leif mpdd 0 fastq-dump --gzip --split-files SRR326097.sra

leif mpdd 0 fastq-dump --gzip --split-files SRR326114.sra

leif mpdd 0 fastq-dump --gzip --split-files SRR326118.sra

leif mpdd 0 fastq-dump --gzip --split-files SRR326121.sra

leif mpdd 0 fastq-dump --gzip --split-files SRR326125.sra

leif mpdd 0 fastq-dump --gzip --split-files SRR326129.sra

leif mpdd 0 fastq-dump --gzip --split-files SRR326132.sra

leif mpdd 0 fastq-dump --gzip --split-files SRR326142.sra

leif mpdd 0 fastq-dump --gzip --split-files SRR326143.sra

leif mpdd 0 fastq-dump --gzip --split-files SRR326150.sra

leif mpdd 0 fastq-dump --gzip --split-files SRR326152.sra

leif mpdd 0 fastq-dump --gzip --split-files SRR326153.sra

leif mpdd 0 fastq-dump --gzip --split-files SRR326154.sra

leif mpdd 0 fastq-dump --gzip --split-files SRR326079.sra

leif mpdd 0 fastq-dump --gzip --split-files SRR326080.sra

leif mpdd 0 fastq-dump --gzip --split-files SRR326106.sra

leif mpdd 0 fastq-dump --gzip --split-files SRR326111.sra

leif mpdd 0 fastq-dump --gzip --split-files SRR326115.sra

leif mpdd 0 fastq-dump --gzip --split-files SRR326124.sra

leif mpdd 0 fastq-dump --gzip --split-files SRR326126.sra

leif mpdd 0 fastq-dump --gzip --split-files SRR326127.sra

leif mpdd 0 fastq-dump --gzip --split-files SRR326131.sra

leif mpdd 0 fastq-dump --gzip --split-files SRR845282.sra

leif mpdd 0 fastq-dump --gzip --split-files SRR845283.sra

leif mpdd 0 fastq-dump --gzip --split-files SRR538771.sra

leif mpdd 0 fastq-dump --gzip --split-files SRR538775.sra

leif mpdd 0 fastq-dump --gzip --split-files SRR641732.sra

leif mpdd 0 fastq-dump --gzip --split-files SRR640891.sra

leif mpdd 0 fastq-dump --gzip --split-files SRR647106.sra

leif mpdd 0 fastq-dump --gzip --split-files SRR647101.sra

leif mpdd 0 fastq-dump --gzip --split-files SRR845263.sra

leif mpdd 0 fastq-dump --gzip --split-files SRR845264.sra

leif mpdd 0 fastq-dump --gzip --split-files SRR640895.sra

leif mpdd 0 fastq-dump --gzip --split-files SRR641731.sra

leif mpdd 0 fastq-dump --gzip --split-files SRR641730.sra

leif mpdd 0 fastq-dump --gzip --split-files SRR641728.sra

leif mpdd 0 fastq-dump --gzip --split-files SRR845261.sra

leif mpdd 0 fastq-dump --gzip --split-files SRR845262.sra

leif mpdd 0 fastq-dump --gzip --split-files SRR647109.sra

leif mpdd 0 fastq-dump --gzip --split-files SRR647103.sra

leif mpdd 0 fastq-dump --gzip --split-files SRR845182.sra

leif mpdd 0 fastq-dump --gzip --split-files SRR640890.sra

leif mpdd 0 fastq-dump --gzip --split-files SRR641735.sra

leif mpdd 0 fastq-dump --gzip --split-files SRR845181.sra

leif mpdd 0 fastq-dump --gzip --split-files SRR845137.sra

leif mpdd 0 fastq-dump --gzip --split-files SRR845136.sra

leif mpdd 0 fastq-dump --gzip --split-files SRR641729.sra

leif mpdd 0 fastq-dump --gzip --split-files SRR640892.sra

leif mpdd 0 fastq-dump --gzip --split-files SRR647108.sra

leif mpdd 0 fastq-dump --gzip --split-files SRR845179.sra

leif mpdd 0 fastq-dump --gzip --split-files SRR845180.sra

leif mpdd 0 fastq-dump --gzip --split-files SRR629744.sra

leif mpdd 0 fastq-dump --gzip --split-files SRR629743.sra

leif mpdd 0 fastq-dump --gzip --split-files SRR646259.sra

leif mpdd 0 fastq-dump --gzip --split-files SRR646260.sra

leif mpdd 0 fastq-dump --gzip --split-files SRR646258.sra

leif mpdd 0 fastq-dump --gzip --split-files SRR640897.sra

leif mpdd 0 fastq-dump --gzip --split-files SRR640896.sra

leif mpdd 0 fastq-dump --gzip --split-files SRR845203.sra

leif mpdd 0 fastq-dump --gzip --split-files SRR845204.sra

leif mpdd 0 fastq-dump --gzip --split-files SRR647105.sra

leif mpdd 0 fastq-dump --gzip --split-files SRR845269.sra

leif mpdd 0 fastq-dump --gzip --split-files SRR845272.sra

leif mpdd 0 fastq-dump --gzip --split-files SRR647102.sra

leif mpdd 0 fastq-dump --gzip --split-files SRR641734.sra

leif mpdd 0 fastq-dump --gzip --split-files SRR641733.sra

leif mpdd 0 fastq-dump --gzip --split-files SRR538776.sra

leif mpdd 0 fastq-dump --gzip --split-files SRR538773.sra

leif mpdd 0 fastq-dump --gzip --split-files SRR538779.sra

leif mpdd 0 fastq-dump --gzip --split-files SRR538780.sra

leif mpdd 0 fastq-dump --gzip --split-files SRR641727.sra

leif mpdd 0 fastq-dump --gzip --split-files SRR640893.sra

leif mpdd 0 fastq-dump --gzip --split-files SRR647107.sra

leif mpdd 0 fastq-dump --gzip --split-files SRR845223.sra

leif mpdd 0 fastq-dump --gzip --split-files SRR845222.sra

leif mpdd 0 fastq-dump --gzip --split-files SRR845177.sra

leif mpdd 0 fastq-dump --gzip --split-files SRR845178.sra

leif mpdd 0 fastq-dump --gzip --split-files SRR647104.sra

leif mpdd 0 fastq-dump --gzip --split-files SRR640894.sra

leif mpdd 0 fastq-dump --gzip --split-files SRR641726.sra

leif mpdd 0 fastq-dump --gzip --split-files SRR538777.sra

leif mpdd 0 fastq-dump --gzip --split-files SRR538772.sra

leif mpdd 0 fastq-dump --gzip --split-files SRR530262.sra

leif mpdd 0 fastq-dump --gzip --split-files SRR530263.sra

leif mpdd 0

echo Started Leif Microbiome Analyzer setup on %date% at %time%

:: Build compact binary file "taxid.git" (~0.3 GB).

gzip -f -d gi_taxid_nucl.dmp.gz

gzip -f -d taxdump.tar.gz

tar xvf taxdump.tar nodes.dmp

tar xvf taxdump.tar names.dmp

leif taxid taxid.git nodes.dmp names.dmp gi_taxid_nucl.dmp

:: Build filter dictionnary for candida & phage sequences (~2 GB).

leif mpdd 0 leif fasta2fa phage.fa blast_nt.fa.gz taxid.git 10841

leif mpdd 0 leif fasta2fa candida_0.fa blast_nt.fa.gz taxid.git 5475

leif mpdd 0 leif fasta2fa candida_1.fa blast_other_genomic.fa.gz taxid.git 5475

leif mpdd 0 leif fasta2fa candida_2.fa blast_wgs.fa.gz taxid.git 5475

leif mpdd 0

leif fasta2fd phage.fd phage.fa

leif fasta2fd candida.fd candida_?.fa

:: Check NCBI BLAST files for gi2taxid consitency.

leif mpdd 0 leif facheck blast_wgs.txt blast_wgs.fa.gz taxid.git

leif mpdd 0 leif facheck blast_other_genomic.txt blast_other_genomic.fa.gz taxid.git

leif mpdd 0 leif facheck blast_human_genomic.txt blast_human_genomic.fa.gz taxid.git

leif mpdd 0 leif facheck blast_nt.txt blast_nt.fa.gz taxid.git

leif mpdd 0

echo Started Leif Microbiome Analyzer analysis on %date% at %time%

:: Align to candida sequences (and phage); discard matching read pairs (part 1).

leif mpdd 0 leif fastq2fx SRR538787_step1.fx "#" SRR538787_1.fastq.gz SRR538787_2.fastq.gz candida.fd phage.fd

leif mpdd 0 leif fastq2fx SRR538782_step1.fx "#" SRR538782_1.fastq.gz SRR538782_2.fastq.gz candida.fd phage.fd

leif mpdd 0 leif fastq2fx SRR538784_step1.fx "#" SRR538784_1.fastq.gz SRR538784_2.fastq.gz candida.fd phage.fd

leif mpdd 0 leif fastq2fx SRR538788_step1.fx "#" SRR538788_1.fastq.gz SRR538788_2.fastq.gz candida.fd phage.fd

leif mpdd 0 leif fastq2fx SRR538786_step1.fx "#" SRR538786_1.fastq.gz SRR538786_2.fastq.gz candida.fd phage.fd

leif mpdd 0 leif fastq2fx SRR538774_step1.fx "#" SRR538774_1.fastq.gz SRR538774_2.fastq.gz candida.fd phage.fd

leif mpdd 0 leif fastq2fx SRR538778_step1.fx "#" SRR538778_1.fastq.gz SRR538778_2.fastq.gz candida.fd phage.fd

leif mpdd 0 leif fastq2fx SRR538781_step1.fx "#" SRR538781_1.fastq.gz SRR538781_2.fastq.gz candida.fd phage.fd

leif mpdd 0 leif fastq2fx SRR538783_step1.fx "#" SRR538783_1.fastq.gz SRR538783_2.fastq.gz candida.fd phage.fd

leif mpdd 0 leif fastq2fx SRR393530_step1.fx "#" SRR393530_1.fastq.gz SRR393530_2.fastq.gz candida.fd phage.fd

leif mpdd 0 leif fastq2fx SRR543721_step1.fx "#" SRR543721_1.fastq.gz SRR543721_2.fastq.gz candida.fd phage.fd

leif mpdd 0 leif fastq2fx SRR543723_step1.fx "#" SRR543723_1.fastq.gz SRR543723_2.fastq.gz candida.fd phage.fd

leif mpdd 0 leif fastq2fx SRR393524_step1.fx "#" SRR393524_1.fastq.gz SRR393524_2.fastq.gz candida.fd phage.fd

leif mpdd 0 leif fastq2fx SRR392813_step1.fx "#" SRR392813_1.fastq.gz SRR392813_2.fastq.gz candida.fd phage.fd

leif mpdd 0 leif fastq2fx SRR630037_step1.fx "#" SRR630037_1.fastq.gz SRR630037_2.fastq.gz candida.fd phage.fd

leif mpdd 0 leif fastq2fx SRR393526_step1.fx "#" SRR393526_1.fastq.gz SRR393526_2.fastq.gz candida.fd phage.fd

leif mpdd 0 leif fastq2fx SRR540285_step1.fx "#" SRR540285_1.fastq.gz SRR540285_2.fastq.gz candida.fd phage.fd

leif mpdd 0 leif fastq2fx SRR540283_step1.fx "#" SRR540283_1.fastq.gz SRR540283_2.fastq.gz candida.fd phage.fd

leif mpdd 0 leif fastq2fx SRR393518_step1.fx "#" SRR393518_1.fastq.gz SRR393518_2.fastq.gz candida.fd phage.fd

leif mpdd 0 leif fastq2fx SRR543729_step1.fx "#" SRR543729_1.fastq.gz SRR543729_2.fastq.gz candida.fd phage.fd

leif mpdd 0 leif fastq2fx SRR393525_step1.fx "#" SRR393525_1.fastq.gz SRR393525_2.fastq.gz candida.fd phage.fd

leif mpdd 0 leif fastq2fx SRR540281_step1.fx "#" SRR540281_1.fastq.gz SRR540281_2.fastq.gz candida.fd phage.fd

leif mpdd 0 leif fastq2fx SRR393517_step1.fx "#" SRR393517_1.fastq.gz SRR393517_2.fastq.gz candida.fd phage.fd

leif mpdd 0 leif fastq2fx SRR393527_step1.fx "#" SRR393527_1.fastq.gz SRR393527_2.fastq.gz candida.fd phage.fd

leif mpdd 0 leif fastq2fx SRR543731_step1.fx "#" SRR543731_1.fastq.gz SRR543731_2.fastq.gz candida.fd phage.fd

leif mpdd 0 leif fastq2fx SRR392814_step1.fx "#" SRR392814_1.fastq.gz SRR392814_2.fastq.gz candida.fd phage.fd

leif mpdd 0 leif fastq2fx SRR393514_step1.fx "#" SRR393514_1.fastq.gz SRR393514_2.fastq.gz candida.fd phage.fd

leif mpdd 0 leif fastq2fx SRR543727_step1.fx "#" SRR543727_1.fastq.gz SRR543727_2.fastq.gz candida.fd phage.fd

leif mpdd 0 leif fastq2fx SRR393515_step1.fx "#" SRR393515_1.fastq.gz SRR393515_2.fastq.gz candida.fd phage.fd

leif mpdd 0 leif fastq2fx SRR540280_step1.fx "#" SRR540280_1.fastq.gz SRR540280_2.fastq.gz candida.fd phage.fd

leif mpdd 0 leif fastq2fx SRR543726_step1.fx "#" SRR543726_1.fastq.gz SRR543726_2.fastq.gz candida.fd phage.fd

leif mpdd 0 leif fastq2fx SRR393520_step1.fx "#" SRR393520_1.fastq.gz SRR393520_2.fastq.gz candida.fd phage.fd

leif mpdd 0 leif fastq2fx SRR393521_step1.fx "#" SRR393521_1.fastq.gz SRR393521_2.fastq.gz candida.fd phage.fd

leif mpdd 0 leif fastq2fx SRR543720_step1.fx "#" SRR543720_1.fastq.gz SRR543720_2.fastq.gz candida.fd phage.fd

leif mpdd 0 leif fastq2fx SRR540284_step1.fx "#" SRR540284_1.fastq.gz SRR540284_2.fastq.gz candida.fd phage.fd

leif mpdd 0 leif fastq2fx SRR393523_step1.fx "#" SRR393523_1.fastq.gz SRR393523_2.fastq.gz candida.fd phage.fd

leif mpdd 0 leif fastq2fx SRR543722_step1.fx "#" SRR543722_1.fastq.gz SRR543722_2.fastq.gz candida.fd phage.fd

leif mpdd 0 leif fastq2fx SRR393528_step1.fx "#" SRR393528_1.fastq.gz SRR393528_2.fastq.gz candida.fd phage.fd

leif mpdd 0 leif fastq2fx SRR543725_step1.fx "#" SRR543725_1.fastq.gz SRR543725_2.fastq.gz candida.fd phage.fd

leif mpdd 0 leif fastq2fx SRR397731_step1.fx "#" SRR397731_1.fastq.gz SRR397731_2.fastq.gz candida.fd phage.fd

leif mpdd 0 leif fastq2fx SRR393516_step1.fx "#" SRR393516_1.fastq.gz SRR393516_2.fastq.gz candida.fd phage.fd

leif mpdd 0 leif fastq2fx SRR543728_step1.fx "#" SRR543728_1.fastq.gz SRR543728_2.fastq.gz candida.fd phage.fd

leif mpdd 0 leif fastq2fx SRR540282_step1.fx "#" SRR540282_1.fastq.gz SRR540282_2.fastq.gz candida.fd phage.fd

leif mpdd 0 leif fastq2fx SRR393519_step1.fx "#" SRR393519_1.fastq.gz SRR393519_2.fastq.gz candida.fd phage.fd

leif mpdd 0 leif fastq2fx SRR848045_step1.fx "#" SRR848045_1.fastq.gz SRR848045_2.fastq.gz candida.fd phage.fd

leif mpdd 0 leif fastq2fx SRR848046_step1.fx "#" SRR848046_1.fastq.gz SRR848046_2.fastq.gz candida.fd phage.fd

leif mpdd 0 leif fastq2fx SRR543724_step1.fx "#" SRR543724_1.fastq.gz SRR543724_2.fastq.gz candida.fd phage.fd

leif mpdd 0 leif fastq2fx SRR393529_step1.fx "#" SRR393529_1.fastq.gz SRR393529_2.fastq.gz candida.fd phage.fd

leif mpdd 0 leif fastq2fx SRR393522_step1.fx "#" SRR393522_1.fastq.gz SRR393522_2.fastq.gz candida.fd phage.fd

leif mpdd 0 leif fastq2fx SRR543730_step1.fx "#" SRR543730_1.fastq.gz SRR543730_2.fastq.gz candida.fd phage.fd

leif mpdd 0 leif fastq2fx SRR317500_step1.fx "#" SRR317500_1.fastq.gz SRR317500_3.fastq.gz candida.fd phage.fd

leif mpdd 0 leif fastq2fx SRR326122_step1.fx "#" SRR326122_1.fastq.gz SRR326122_3.fastq.gz candida.fd phage.fd

leif mpdd 0 leif fastq2fx SRR317501_step1.fx "#" SRR317501_1.fastq.gz SRR317501_3.fastq.gz candida.fd phage.fd

leif mpdd 0 leif fastq2fx SRR326077_step1.fx "#" SRR326077_1.fastq.gz SRR326077_3.fastq.gz candida.fd phage.fd

leif mpdd 0 leif fastq2fx SRR326090_step1.fx "#" SRR326090_1.fastq.gz SRR326090_3.fastq.gz candida.fd phage.fd

leif mpdd 0 leif fastq2fx SRR326091_step1.fx "#" SRR326091_1.fastq.gz SRR326091_3.fastq.gz candida.fd phage.fd

leif mpdd 0 leif fastq2fx SRR326092_step1.fx "#" SRR326092_1.fastq.gz SRR326092_3.fastq.gz candida.fd phage.fd

leif mpdd 0 leif fastq2fx SRR326095_step1.fx "#" SRR326095_1.fastq.gz SRR326095_3.fastq.gz candida.fd phage.fd

leif mpdd 0 leif fastq2fx SRR326097_step1.fx "#" SRR326097_1.fastq.gz SRR326097_3.fastq.gz candida.fd phage.fd

leif mpdd 0 leif fastq2fx SRR326114_step1.fx "#" SRR326114_1.fastq.gz SRR326114_3.fastq.gz candida.fd phage.fd

leif mpdd 0 leif fastq2fx SRR326118_step1.fx "#" SRR326118_1.fastq.gz SRR326118_3.fastq.gz candida.fd phage.fd

leif mpdd 0 leif fastq2fx SRR326121_step1.fx "#" SRR326121_1.fastq.gz SRR326121_3.fastq.gz candida.fd phage.fd

leif mpdd 0 leif fastq2fx SRR326125_step1.fx "#" SRR326125_1.fastq.gz SRR326125_3.fastq.gz candida.fd phage.fd

leif mpdd 0 leif fastq2fx SRR326129_step1.fx "#" SRR326129_1.fastq.gz SRR326129_3.fastq.gz candida.fd phage.fd

leif mpdd 0 leif fastq2fx SRR326132_step1.fx "#" SRR326132_1.fastq.gz SRR326132_3.fastq.gz candida.fd phage.fd

leif mpdd 0 leif fastq2fx SRR326142_step1.fx "#" SRR326142_1.fastq.gz SRR326142_3.fastq.gz candida.fd phage.fd

leif mpdd 0 leif fastq2fx SRR326143_step1.fx "#" SRR326143_1.fastq.gz SRR326143_3.fastq.gz candida.fd phage.fd

leif mpdd 0 leif fastq2fx SRR326150_step1.fx "#" SRR326150_1.fastq.gz SRR326150_3.fastq.gz candida.fd phage.fd

leif mpdd 0 leif fastq2fx SRR326152_step1.fx "#" SRR326152_1.fastq.gz SRR326152_3.fastq.gz candida.fd phage.fd

leif mpdd 0 leif fastq2fx SRR326153_step1.fx "#" SRR326153_1.fastq.gz SRR326153_3.fastq.gz candida.fd phage.fd

leif mpdd 0 leif fastq2fx SRR326154_step1.fx "#" SRR326154_1.fastq.gz SRR326154_3.fastq.gz candida.fd phage.fd

leif mpdd 0 leif fastq2fx SRR326079_step1.fx "#" SRR326079_1.fastq.gz SRR326079_3.fastq.gz candida.fd phage.fd

leif mpdd 0 leif fastq2fx SRR326080_step1.fx "#" SRR326080_1.fastq.gz SRR326080_3.fastq.gz candida.fd phage.fd

leif mpdd 0 leif fastq2fx SRR326106_step1.fx "#" SRR326106_1.fastq.gz SRR326106_3.fastq.gz candida.fd phage.fd

leif mpdd 0 leif fastq2fx SRR326111_step1.fx "#" SRR326111_1.fastq.gz SRR326111_3.fastq.gz candida.fd phage.fd

leif mpdd 0 leif fastq2fx SRR326115_step1.fx "#" SRR326115_1.fastq.gz SRR326115_3.fastq.gz candida.fd phage.fd

leif mpdd 0 leif fastq2fx SRR326124_step1.fx "#" SRR326124_1.fastq.gz SRR326124_3.fastq.gz candida.fd phage.fd

leif mpdd 0 leif fastq2fx SRR326126_step1.fx "#" SRR326126_1.fastq.gz SRR326126_3.fastq.gz candida.fd phage.fd

leif mpdd 0 leif fastq2fx SRR326127_step1.fx "#" SRR326127_1.fastq.gz SRR326127_3.fastq.gz candida.fd phage.fd

leif mpdd 0 leif fastq2fx SRR326131_step1.fx "#" SRR326131_1.fastq.gz SRR326131_3.fastq.gz candida.fd phage.fd

leif mpdd 0 leif fastq2fx SRR845282_step1.fx "#" SRR845282_1.fastq.gz SRR845282_2.fastq.gz candida.fd phage.fd

leif mpdd 0 leif fastq2fx SRR845283_step1.fx "#" SRR845283_1.fastq.gz SRR845283_2.fastq.gz candida.fd phage.fd

leif mpdd 0 leif fastq2fx SRR538771_step1.fx "#" SRR538771_1.fastq.gz SRR538771_2.fastq.gz candida.fd phage.fd

leif mpdd 0 leif fastq2fx SRR538775_step1.fx "#" SRR538775_1.fastq.gz SRR538775_2.fastq.gz candida.fd phage.fd

leif mpdd 0 leif fastq2fx SRR641732_step1.fx "#" SRR641732_1.fastq.gz SRR641732_2.fastq.gz candida.fd phage.fd

leif mpdd 0 leif fastq2fx SRR640891_step1.fx "#" SRR640891_1.fastq.gz SRR640891_2.fastq.gz candida.fd phage.fd

leif mpdd 0 leif fastq2fx SRR647106_step1.fx "#" SRR647106_1.fastq.gz SRR647106_2.fastq.gz candida.fd phage.fd

leif mpdd 0 leif fastq2fx SRR647101_step1.fx "#" SRR647101_1.fastq.gz SRR647101_2.fastq.gz candida.fd phage.fd

leif mpdd 0 leif fastq2fx SRR845263_step1.fx "#" SRR845263_1.fastq.gz SRR845263_2.fastq.gz candida.fd phage.fd

leif mpdd 0 leif fastq2fx SRR845264_step1.fx "#" SRR845264_1.fastq.gz SRR845264_2.fastq.gz candida.fd phage.fd

leif mpdd 0 leif fastq2fx SRR640895_step1.fx "#" SRR640895_1.fastq.gz SRR640895_2.fastq.gz candida.fd phage.fd

leif mpdd 0 leif fastq2fx SRR641731_step1.fx "#" SRR641731_1.fastq.gz SRR641731_2.fastq.gz candida.fd phage.fd

leif mpdd 0 leif fastq2fx SRR641730_step1.fx "#" SRR641730_1.fastq.gz SRR641730_2.fastq.gz candida.fd phage.fd

leif mpdd 0 leif fastq2fx SRR641728_step1.fx "#" SRR641728_1.fastq.gz SRR641728_2.fastq.gz candida.fd phage.fd

leif mpdd 0 leif fastq2fx SRR845261_step1.fx "#" SRR845261_1.fastq.gz SRR845261_2.fastq.gz candida.fd phage.fd

leif mpdd 0 leif fastq2fx SRR845262_step1.fx "#" SRR845262_1.fastq.gz SRR845262_2.fastq.gz candida.fd phage.fd

leif mpdd 0 leif fastq2fx SRR647109_step1.fx "#" SRR647109_1.fastq.gz SRR647109_2.fastq.gz candida.fd phage.fd

leif mpdd 0 leif fastq2fx SRR647103_step1.fx "#" SRR647103_1.fastq.gz SRR647103_2.fastq.gz candida.fd phage.fd

leif mpdd 0 leif fastq2fx SRR845182_step1.fx "#" SRR845182_1.fastq.gz SRR845182_2.fastq.gz candida.fd phage.fd

leif mpdd 0 leif fastq2fx SRR640890_step1.fx "#" SRR640890_1.fastq.gz SRR640890_2.fastq.gz candida.fd phage.fd

leif mpdd 0 leif fastq2fx SRR641735_step1.fx "#" SRR641735_1.fastq.gz SRR641735_2.fastq.gz candida.fd phage.fd

leif mpdd 0 leif fastq2fx SRR845181_step1.fx "#" SRR845181_1.fastq.gz SRR845181_2.fastq.gz candida.fd phage.fd

leif mpdd 0 leif fastq2fx SRR845137_step1.fx "#" SRR845137_1.fastq.gz SRR845137_2.fastq.gz candida.fd phage.fd

leif mpdd 0 leif fastq2fx SRR845136_step1.fx "#" SRR845136_1.fastq.gz SRR845136_2.fastq.gz candida.fd phage.fd

leif mpdd 0 leif fastq2fx SRR641729_step1.fx "#" SRR641729_1.fastq.gz SRR641729_2.fastq.gz candida.fd phage.fd

leif mpdd 0 leif fastq2fx SRR640892_step1.fx "#" SRR640892_1.fastq.gz SRR640892_2.fastq.gz candida.fd phage.fd

leif mpdd 0 leif fastq2fx SRR647108_step1.fx "#" SRR647108_1.fastq.gz SRR647108_2.fastq.gz candida.fd phage.fd

leif mpdd 0 leif fastq2fx SRR845179_step1.fx "#" SRR845179_1.fastq.gz SRR845179_2.fastq.gz candida.fd phage.fd

leif mpdd 0 leif fastq2fx SRR845180_step1.fx "#" SRR845180_1.fastq.gz SRR845180_2.fastq.gz candida.fd phage.fd

leif mpdd 0 leif fastq2fx SRR629744_step1.fx "#" SRR629744_1.fastq.gz SRR629744_2.fastq.gz candida.fd phage.fd

leif mpdd 0 leif fastq2fx SRR629743_step1.fx "#" SRR629743_1.fastq.gz SRR629743_2.fastq.gz candida.fd phage.fd

leif mpdd 0 leif fastq2fx SRR646259_step1.fx "#" SRR646259_1.fastq.gz SRR646259_2.fastq.gz candida.fd phage.fd

leif mpdd 0 leif fastq2fx SRR646260_step1.fx "#" SRR646260_1.fastq.gz SRR646260_2.fastq.gz candida.fd phage.fd

leif mpdd 0 leif fastq2fx SRR646258_step1.fx "#" SRR646258_1.fastq.gz SRR646258_2.fastq.gz candida.fd phage.fd

leif mpdd 0 leif fastq2fx SRR640897_step1.fx "#" SRR640897_1.fastq.gz SRR640897_2.fastq.gz candida.fd phage.fd

leif mpdd 0 leif fastq2fx SRR640896_step1.fx "#" SRR640896_1.fastq.gz SRR640896_2.fastq.gz candida.fd phage.fd

leif mpdd 0 leif fastq2fx SRR845203_step1.fx "#" SRR845203_1.fastq.gz SRR845203_2.fastq.gz candida.fd phage.fd

leif mpdd 0 leif fastq2fx SRR845204_step1.fx "#" SRR845204_1.fastq.gz SRR845204_2.fastq.gz candida.fd phage.fd

leif mpdd 0 leif fastq2fx SRR647105_step1.fx "#" SRR647105_1.fastq.gz SRR647105_2.fastq.gz candida.fd phage.fd

leif mpdd 0 leif fastq2fx SRR845269_step1.fx "#" SRR845269_1.fastq.gz SRR845269_2.fastq.gz candida.fd phage.fd

leif mpdd 0 leif fastq2fx SRR845272_step1.fx "#" SRR845272_1.fastq.gz SRR845272_2.fastq.gz candida.fd phage.fd

leif mpdd 0 leif fastq2fx SRR647102_step1.fx "#" SRR647102_1.fastq.gz SRR647102_2.fastq.gz candida.fd phage.fd

leif mpdd 0 leif fastq2fx SRR641734_step1.fx "#" SRR641734_1.fastq.gz SRR641734_2.fastq.gz candida.fd phage.fd

leif mpdd 0 leif fastq2fx SRR641733_step1.fx "#" SRR641733_1.fastq.gz SRR641733_2.fastq.gz candida.fd phage.fd

leif mpdd 0 leif fastq2fx SRR538776_step1.fx "#" SRR538776_1.fastq.gz SRR538776_2.fastq.gz candida.fd phage.fd

leif mpdd 0 leif fastq2fx SRR538773_step1.fx "#" SRR538773_1.fastq.gz SRR538773_2.fastq.gz candida.fd phage.fd

leif mpdd 0 leif fastq2fx SRR538779_step1.fx "#" SRR538779_1.fastq.gz SRR538779_2.fastq.gz candida.fd phage.fd

leif mpdd 0 leif fastq2fx SRR538780_step1.fx "#" SRR538780_1.fastq.gz SRR538780_2.fastq.gz candida.fd phage.fd

leif mpdd 0 leif fastq2fx SRR641727_step1.fx "#" SRR641727_1.fastq.gz SRR641727_2.fastq.gz candida.fd phage.fd

leif mpdd 0 leif fastq2fx SRR640893_step1.fx "#" SRR640893_1.fastq.gz SRR640893_2.fastq.gz candida.fd phage.fd

leif mpdd 0 leif fastq2fx SRR647107_step1.fx "#" SRR647107_1.fastq.gz SRR647107_2.fastq.gz candida.fd phage.fd

leif mpdd 0 leif fastq2fx SRR845223_step1.fx "#" SRR845223_1.fastq.gz SRR845223_2.fastq.gz candida.fd phage.fd

leif mpdd 0 leif fastq2fx SRR845222_step1.fx "#" SRR845222_1.fastq.gz SRR845222_2.fastq.gz candida.fd phage.fd

leif mpdd 0 leif fastq2fx SRR845177_step1.fx "#" SRR845177_1.fastq.gz SRR845177_2.fastq.gz candida.fd phage.fd

leif mpdd 0 leif fastq2fx SRR845178_step1.fx "#" SRR845178_1.fastq.gz SRR845178_2.fastq.gz candida.fd phage.fd

leif mpdd 0 leif fastq2fx SRR647104_step1.fx "#" SRR647104_1.fastq.gz SRR647104_2.fastq.gz candida.fd phage.fd

leif mpdd 0 leif fastq2fx SRR640894_step1.fx "#" SRR640894_1.fastq.gz SRR640894_2.fastq.gz candida.fd phage.fd

leif mpdd 0 leif fastq2fx SRR641726_step1.fx "#" SRR641726_1.fastq.gz SRR641726_2.fastq.gz candida.fd phage.fd

leif mpdd 0 leif fastq2fx SRR538777_step1.fx "#" SRR538777_1.fastq.gz SRR538777_2.fastq.gz candida.fd phage.fd

leif mpdd 0 leif fastq2fx SRR538772_step1.fx "#" SRR538772_1.fastq.gz SRR538772_2.fastq.gz candida.fd phage.fd

leif mpdd 0 leif fastq2fx SRR530262_step1.fx "#" SRR530262_1.fastq.gz SRR530262_2.fastq.gz candida.fd phage.fd

leif mpdd 0 leif fastq2fx SRR530263_step1.fx "#" SRR530263_1.fastq.gz SRR530263_2.fastq.gz candida.fd phage.fd

leif mpdd 0

echo %date% %time%

:: Align to human sequences (and EBV/phage); discard matching read pairs (part 2).

leif mpdd 0 leif fx2fx SRR538787_step2.fx SRR538787_step1.fx candida.fd phage.fd

leif mpdd 0 leif fx2fx SRR538782_step2.fx SRR538782_step1.fx candida.fd phage.fd

leif mpdd 0 leif fx2fx SRR538784_step2.fx SRR538784_step1.fx candida.fd phage.fd

leif mpdd 0 leif fx2fx SRR538788_step2.fx SRR538788_step1.fx candida.fd phage.fd

leif mpdd 0 leif fx2fx SRR538786_step2.fx SRR538786_step1.fx candida.fd phage.fd

leif mpdd 0 leif fx2fx SRR538774_step2.fx SRR538774_step1.fx candida.fd phage.fd

leif mpdd 0 leif fx2fx SRR538778_step2.fx SRR538778_step1.fx candida.fd phage.fd

leif mpdd 0 leif fx2fx SRR538781_step2.fx SRR538781_step1.fx candida.fd phage.fd

leif mpdd 0 leif fx2fx SRR538783_step2.fx SRR538783_step1.fx candida.fd phage.fd

leif mpdd 0 leif fx2fx SRR393530_step2.fx SRR393530_step1.fx candida.fd phage.fd

leif mpdd 0 leif fx2fx SRR543721_step2.fx SRR543721_step1.fx candida.fd phage.fd

leif mpdd 0 leif fx2fx SRR543723_step2.fx SRR543723_step1.fx candida.fd phage.fd

leif mpdd 0 leif fx2fx SRR393524_step2.fx SRR393524_step1.fx candida.fd phage.fd

leif mpdd 0 leif fx2fx SRR392813_step2.fx SRR392813_step1.fx candida.fd phage.fd

leif mpdd 0 leif fx2fx SRR630037_step2.fx SRR630037_step1.fx candida.fd phage.fd

leif mpdd 0 leif fx2fx SRR393526_step2.fx SRR393526_step1.fx candida.fd phage.fd

leif mpdd 0 leif fx2fx SRR540285_step2.fx SRR540285_step1.fx candida.fd phage.fd

leif mpdd 0 leif fx2fx SRR540283_step2.fx SRR540283_step1.fx candida.fd phage.fd

leif mpdd 0 leif fx2fx SRR393518_step2.fx SRR393518_step1.fx candida.fd phage.fd

leif mpdd 0 leif fx2fx SRR543729_step2.fx SRR543729_step1.fx candida.fd phage.fd

leif mpdd 0 leif fx2fx SRR393525_step2.fx SRR393525_step1.fx candida.fd phage.fd

leif mpdd 0 leif fx2fx SRR540281_step2.fx SRR540281_step1.fx candida.fd phage.fd

leif mpdd 0 leif fx2fx SRR393517_step2.fx SRR393517_step1.fx candida.fd phage.fd

leif mpdd 0 leif fx2fx SRR393527_step2.fx SRR393527_step1.fx candida.fd phage.fd

leif mpdd 0 leif fx2fx SRR543731_step2.fx SRR543731_step1.fx candida.fd phage.fd

leif mpdd 0 leif fx2fx SRR392814_step2.fx SRR392814_step1.fx candida.fd phage.fd

leif mpdd 0 leif fx2fx SRR393514_step2.fx SRR393514_step1.fx candida.fd phage.fd

leif mpdd 0 leif fx2fx SRR543727_step2.fx SRR543727_step1.fx candida.fd phage.fd

leif mpdd 0 leif fx2fx SRR393515_step2.fx SRR393515_step1.fx candida.fd phage.fd

leif mpdd 0 leif fx2fx SRR540280_step2.fx SRR540280_step1.fx candida.fd phage.fd

leif mpdd 0 leif fx2fx SRR543726_step2.fx SRR543726_step1.fx candida.fd phage.fd

leif mpdd 0 leif fx2fx SRR393520_step2.fx SRR393520_step1.fx candida.fd phage.fd

leif mpdd 0 leif fx2fx SRR393521_step2.fx SRR393521_step1.fx candida.fd phage.fd

leif mpdd 0 leif fx2fx SRR543720_step2.fx SRR543720_step1.fx candida.fd phage.fd

leif mpdd 0 leif fx2fx SRR540284_step2.fx SRR540284_step1.fx candida.fd phage.fd

leif mpdd 0 leif fx2fx SRR393523_step2.fx SRR393523_step1.fx candida.fd phage.fd

leif mpdd 0 leif fx2fx SRR543722_step2.fx SRR543722_step1.fx candida.fd phage.fd

leif mpdd 0 leif fx2fx SRR393528_step2.fx SRR393528_step1.fx candida.fd phage.fd

leif mpdd 0 leif fx2fx SRR543725_step2.fx SRR543725_step1.fx candida.fd phage.fd

leif mpdd 0 leif fx2fx SRR397731_step2.fx SRR397731_step1.fx candida.fd phage.fd

leif mpdd 0 leif fx2fx SRR393516_step2.fx SRR393516_step1.fx candida.fd phage.fd

leif mpdd 0 leif fx2fx SRR543728_step2.fx SRR543728_step1.fx candida.fd phage.fd

leif mpdd 0 leif fx2fx SRR540282_step2.fx SRR540282_step1.fx candida.fd phage.fd

leif mpdd 0 leif fx2fx SRR393519_step2.fx SRR393519_step1.fx candida.fd phage.fd

leif mpdd 0 leif fx2fx SRR848045_step2.fx SRR848045_step1.fx candida.fd phage.fd

leif mpdd 0 leif fx2fx SRR848046_step2.fx SRR848046_step1.fx candida.fd phage.fd

leif mpdd 0 leif fx2fx SRR543724_step2.fx SRR543724_step1.fx candida.fd phage.fd

leif mpdd 0 leif fx2fx SRR393529_step2.fx SRR393529_step1.fx candida.fd phage.fd

leif mpdd 0 leif fx2fx SRR393522_step2.fx SRR393522_step1.fx candida.fd phage.fd

leif mpdd 0 leif fx2fx SRR543730_step2.fx SRR543730_step1.fx candida.fd phage.fd

leif mpdd 0 leif fx2fx SRR317500_step2.fx SRR317500_step1.fx candida.fd phage.fd

leif mpdd 0 leif fx2fx SRR326122_step2.fx SRR326122_step1.fx candida.fd phage.fd

leif mpdd 0 leif fx2fx SRR317501_step2.fx SRR317501_step1.fx candida.fd phage.fd

leif mpdd 0 leif fx2fx SRR326077_step2.fx SRR326077_step1.fx candida.fd phage.fd

leif mpdd 0 leif fx2fx SRR326090_step2.fx SRR326090_step1.fx candida.fd phage.fd

leif mpdd 0 leif fx2fx SRR326091_step2.fx SRR326091_step1.fx candida.fd phage.fd

leif mpdd 0 leif fx2fx SRR326092_step2.fx SRR326092_step1.fx candida.fd phage.fd

leif mpdd 0 leif fx2fx SRR326095_step2.fx SRR326095_step1.fx candida.fd phage.fd

leif mpdd 0 leif fx2fx SRR326097_step2.fx SRR326097_step1.fx candida.fd phage.fd

leif mpdd 0 leif fx2fx SRR326114_step2.fx SRR326114_step1.fx candida.fd phage.fd

leif mpdd 0 leif fx2fx SRR326118_step2.fx SRR326118_step1.fx candida.fd phage.fd

leif mpdd 0 leif fx2fx SRR326121_step2.fx SRR326121_step1.fx candida.fd phage.fd

leif mpdd 0 leif fx2fx SRR326125_step2.fx SRR326125_step1.fx candida.fd phage.fd

leif mpdd 0 leif fx2fx SRR326129_step2.fx SRR326129_step1.fx candida.fd phage.fd

leif mpdd 0 leif fx2fx SRR326132_step2.fx SRR326132_step1.fx candida.fd phage.fd

leif mpdd 0 leif fx2fx SRR326142_step2.fx SRR326142_step1.fx candida.fd phage.fd

leif mpdd 0 leif fx2fx SRR326143_step2.fx SRR326143_step1.fx candida.fd phage.fd

leif mpdd 0 leif fx2fx SRR326150_step2.fx SRR326150_step1.fx candida.fd phage.fd

leif mpdd 0 leif fx2fx SRR326152_step2.fx SRR326152_step1.fx candida.fd phage.fd

leif mpdd 0 leif fx2fx SRR326153_step2.fx SRR326153_step1.fx candida.fd phage.fd

leif mpdd 0 leif fx2fx SRR326154_step2.fx SRR326154_step1.fx candida.fd phage.fd

leif mpdd 0 leif fx2fx SRR326079_step2.fx SRR326079_step1.fx candida.fd phage.fd

leif mpdd 0 leif fx2fx SRR326080_step2.fx SRR326080_step1.fx candida.fd phage.fd

leif mpdd 0 leif fx2fx SRR326106_step2.fx SRR326106_step1.fx candida.fd phage.fd

leif mpdd 0 leif fx2fx SRR326111_step2.fx SRR326111_step1.fx candida.fd phage.fd

leif mpdd 0 leif fx2fx SRR326115_step2.fx SRR326115_step1.fx candida.fd phage.fd

leif mpdd 0 leif fx2fx SRR326124_step2.fx SRR326124_step1.fx candida.fd phage.fd

leif mpdd 0 leif fx2fx SRR326126_step2.fx SRR326126_step1.fx candida.fd phage.fd

leif mpdd 0 leif fx2fx SRR326127_step2.fx SRR326127_step1.fx candida.fd phage.fd

leif mpdd 0 leif fx2fx SRR326131_step2.fx SRR326131_step1.fx candida.fd phage.fd

leif mpdd 0 leif fx2fx SRR845282_step2.fx SRR845282_step1.fx candida.fd phage.fd

leif mpdd 0 leif fx2fx SRR845283_step2.fx SRR845283_step1.fx candida.fd phage.fd

leif mpdd 0 leif fx2fx SRR538771_step2.fx SRR538771_step1.fx candida.fd phage.fd

leif mpdd 0 leif fx2fx SRR538775_step2.fx SRR538775_step1.fx candida.fd phage.fd

leif mpdd 0 leif fx2fx SRR641732_step2.fx SRR641732_step1.fx candida.fd phage.fd

leif mpdd 0 leif fx2fx SRR640891_step2.fx SRR640891_step1.fx candida.fd phage.fd

leif mpdd 0 leif fx2fx SRR647106_step2.fx SRR647106_step1.fx candida.fd phage.fd

leif mpdd 0 leif fx2fx SRR647101_step2.fx SRR647101_step1.fx candida.fd phage.fd

leif mpdd 0 leif fx2fx SRR845263_step2.fx SRR845263_step1.fx candida.fd phage.fd

leif mpdd 0 leif fx2fx SRR845264_step2.fx SRR845264_step1.fx candida.fd phage.fd

leif mpdd 0 leif fx2fx SRR640895_step2.fx SRR640895_step1.fx candida.fd phage.fd

leif mpdd 0 leif fx2fx SRR641731_step2.fx SRR641731_step1.fx candida.fd phage.fd

leif mpdd 0 leif fx2fx SRR641730_step2.fx SRR641730_step1.fx candida.fd phage.fd

leif mpdd 0 leif fx2fx SRR641728_step2.fx SRR641728_step1.fx candida.fd phage.fd

leif mpdd 0 leif fx2fx SRR845261_step2.fx SRR845261_step1.fx candida.fd phage.fd

leif mpdd 0 leif fx2fx SRR845262_step2.fx SRR845262_step1.fx candida.fd phage.fd

leif mpdd 0 leif fx2fx SRR647109_step2.fx SRR647109_step1.fx candida.fd phage.fd

leif mpdd 0 leif fx2fx SRR647103_step2.fx SRR647103_step1.fx candida.fd phage.fd

leif mpdd 0 leif fx2fx SRR845182_step2.fx SRR845182_step1.fx candida.fd phage.fd

leif mpdd 0 leif fx2fx SRR640890_step2.fx SRR640890_step1.fx candida.fd phage.fd

leif mpdd 0 leif fx2fx SRR641735_step2.fx SRR641735_step1.fx candida.fd phage.fd

leif mpdd 0 leif fx2fx SRR845181_step2.fx SRR845181_step1.fx candida.fd phage.fd

leif mpdd 0 leif fx2fx SRR845137_step2.fx SRR845137_step1.fx candida.fd phage.fd

leif mpdd 0 leif fx2fx SRR845136_step2.fx SRR845136_step1.fx candida.fd phage.fd

leif mpdd 0 leif fx2fx SRR641729_step2.fx SRR641729_step1.fx candida.fd phage.fd

leif mpdd 0 leif fx2fx SRR640892_step2.fx SRR640892_step1.fx candida.fd phage.fd

leif mpdd 0 leif fx2fx SRR647108_step2.fx SRR647108_step1.fx candida.fd phage.fd

leif mpdd 0 leif fx2fx SRR845179_step2.fx SRR845179_step1.fx candida.fd phage.fd

leif mpdd 0 leif fx2fx SRR845180_step2.fx SRR845180_step1.fx candida.fd phage.fd

leif mpdd 0 leif fx2fx SRR629744_step2.fx SRR629744_step1.fx candida.fd phage.fd

leif mpdd 0 leif fx2fx SRR629743_step2.fx SRR629743_step1.fx candida.fd phage.fd

leif mpdd 0 leif fx2fx SRR646259_step2.fx SRR646259_step1.fx candida.fd phage.fd

leif mpdd 0 leif fx2fx SRR646260_step2.fx SRR646260_step1.fx candida.fd phage.fd

leif mpdd 0 leif fx2fx SRR646258_step2.fx SRR646258_step1.fx candida.fd phage.fd

leif mpdd 0 leif fx2fx SRR640897_step2.fx SRR640897_step1.fx candida.fd phage.fd

leif mpdd 0 leif fx2fx SRR640896_step2.fx SRR640896_step1.fx candida.fd phage.fd

leif mpdd 0 leif fx2fx SRR845203_step2.fx SRR845203_step1.fx candida.fd phage.fd

leif mpdd 0 leif fx2fx SRR845204_step2.fx SRR845204_step1.fx candida.fd phage.fd

leif mpdd 0 leif fx2fx SRR647105_step2.fx SRR647105_step1.fx candida.fd phage.fd

leif mpdd 0 leif fx2fx SRR845269_step2.fx SRR845269_step1.fx candida.fd phage.fd

leif mpdd 0 leif fx2fx SRR845272_step2.fx SRR845272_step1.fx candida.fd phage.fd

leif mpdd 0 leif fx2fx SRR647102_step2.fx SRR647102_step1.fx candida.fd phage.fd

leif mpdd 0 leif fx2fx SRR641734_step2.fx SRR641734_step1.fx candida.fd phage.fd

leif mpdd 0 leif fx2fx SRR641733_step2.fx SRR641733_step1.fx candida.fd phage.fd

leif mpdd 0 leif fx2fx SRR538776_step2.fx SRR538776_step1.fx candida.fd phage.fd

leif mpdd 0 leif fx2fx SRR538773_step2.fx SRR538773_step1.fx candida.fd phage.fd

leif mpdd 0 leif fx2fx SRR538779_step2.fx SRR538779_step1.fx candida.fd phage.fd

leif mpdd 0 leif fx2fx SRR538780_step2.fx SRR538780_step1.fx candida.fd phage.fd

leif mpdd 0 leif fx2fx SRR641727_step2.fx SRR641727_step1.fx candida.fd phage.fd

leif mpdd 0 leif fx2fx SRR640893_step2.fx SRR640893_step1.fx candida.fd phage.fd

leif mpdd 0 leif fx2fx SRR647107_step2.fx SRR647107_step1.fx candida.fd phage.fd

leif mpdd 0 leif fx2fx SRR845223_step2.fx SRR845223_step1.fx candida.fd phage.fd

leif mpdd 0 leif fx2fx SRR845222_step2.fx SRR845222_step1.fx candida.fd phage.fd

leif mpdd 0 leif fx2fx SRR845177_step2.fx SRR845177_step1.fx candida.fd phage.fd

leif mpdd 0 leif fx2fx SRR845178_step2.fx SRR845178_step1.fx candida.fd phage.fd

leif mpdd 0 leif fx2fx SRR647104_step2.fx SRR647104_step1.fx candida.fd phage.fd

leif mpdd 0 leif fx2fx SRR640894_step2.fx SRR640894_step1.fx candida.fd phage.fd

leif mpdd 0 leif fx2fx SRR641726_step2.fx SRR641726_step1.fx candida.fd phage.fd

leif mpdd 0 leif fx2fx SRR538777_step2.fx SRR538777_step1.fx candida.fd phage.fd

leif mpdd 0 leif fx2fx SRR538772_step2.fx SRR538772_step1.fx candida.fd phage.fd

leif mpdd 0 leif fx2fx SRR530262_step2.fx SRR530262_step1.fx candida.fd phage.fd

leif mpdd 0 leif fx2fx SRR530263_step2.fx SRR530263_step1.fx candida.fd phage.fd

leif mpdd 0

echo %date% %time%

:: Eliminate duplicate/clonal read pairs.

leif mpdd 0 leif fxclone 3 5 60 SRR538787_step3.fx SRR538787_clone.fx SRR538787_step2.fx

leif mpdd 0 leif fxclone 3 5 60 SRR538782_step3.fx SRR538782_clone.fx SRR538782_step2.fx

leif mpdd 0 leif fxclone 3 5 60 SRR538784_step3.fx SRR538784_clone.fx SRR538784_step2.fx

leif mpdd 0 leif fxclone 3 5 60 SRR538788_step3.fx SRR538788_clone.fx SRR538788_step2.fx

leif mpdd 0 leif fxclone 3 5 60 SRR538786_step3.fx SRR538786_clone.fx SRR538786_step2.fx

leif mpdd 0 leif fxclone 3 5 60 SRR538774_step3.fx SRR538774_clone.fx SRR538774_step2.fx

leif mpdd 0 leif fxclone 3 5 60 SRR538778_step3.fx SRR538778_clone.fx SRR538778_step2.fx

leif mpdd 0 leif fxclone 3 5 60 SRR538781_step3.fx SRR538781_clone.fx SRR538781_step2.fx

leif mpdd 0 leif fxclone 3 5 60 SRR538783_step3.fx SRR538783_clone.fx SRR538783_step2.fx

leif mpdd 0 leif fxclone 3 5 60 SRR393530_step3.fx SRR393530_clone.fx SRR393530_step2.fx

leif mpdd 0 leif fxclone 3 5 60 SRR543721_step3.fx SRR543721_clone.fx SRR543721_step2.fx

leif mpdd 0 leif fxclone 3 5 60 SRR543723_step3.fx SRR543723_clone.fx SRR543723_step2.fx

leif mpdd 0 leif fxclone 3 5 60 SRR393524_step3.fx SRR393524_clone.fx SRR393524_step2.fx

leif mpdd 0 leif fxclone 3 5 60 SRR392813_step3.fx SRR392813_clone.fx SRR392813_step2.fx

leif mpdd 0 leif fxclone 3 5 60 SRR630037_step3.fx SRR630037_clone.fx SRR630037_step2.fx

leif mpdd 0 leif fxclone 3 5 60 SRR393526_step3.fx SRR393526_clone.fx SRR393526_step2.fx

leif mpdd 0 leif fxclone 3 5 60 SRR540285_step3.fx SRR540285_clone.fx SRR540285_step2.fx

leif mpdd 0 leif fxclone 3 5 60 SRR540283_step3.fx SRR540283_clone.fx SRR540283_step2.fx

leif mpdd 0 leif fxclone 3 5 60 SRR393518_step3.fx SRR393518_clone.fx SRR393518_step2.fx

leif mpdd 0 leif fxclone 3 5 60 SRR543729_step3.fx SRR543729_clone.fx SRR543729_step2.fx

leif mpdd 0 leif fxclone 3 5 60 SRR393525_step3.fx SRR393525_clone.fx SRR393525_step2.fx

leif mpdd 0 leif fxclone 3 5 60 SRR540281_step3.fx SRR540281_clone.fx SRR540281_step2.fx

leif mpdd 0 leif fxclone 3 5 60 SRR393517_step3.fx SRR393517_clone.fx SRR393517_step2.fx

leif mpdd 0 leif fxclone 3 5 60 SRR393527_step3.fx SRR393527_clone.fx SRR393527_step2.fx

leif mpdd 0 leif fxclone 3 5 60 SRR543731_step3.fx SRR543731_clone.fx SRR543731_step2.fx

leif mpdd 0 leif fxclone 3 5 60 SRR392814_step3.fx SRR392814_clone.fx SRR392814_step2.fx

leif mpdd 0 leif fxclone 3 5 60 SRR393514_step3.fx SRR393514_clone.fx SRR393514_step2.fx

leif mpdd 0 leif fxclone 3 5 60 SRR543727_step3.fx SRR543727_clone.fx SRR543727_step2.fx

leif mpdd 0 leif fxclone 3 5 60 SRR393515_step3.fx SRR393515_clone.fx SRR393515_step2.fx

leif mpdd 0 leif fxclone 3 5 60 SRR540280_step3.fx SRR540280_clone.fx SRR540280_step2.fx

leif mpdd 0 leif fxclone 3 5 60 SRR543726_step3.fx SRR543726_clone.fx SRR543726_step2.fx

leif mpdd 0 leif fxclone 3 5 60 SRR393520_step3.fx SRR393520_clone.fx SRR393520_step2.fx

leif mpdd 0 leif fxclone 3 5 60 SRR393521_step3.fx SRR393521_clone.fx SRR393521_step2.fx

leif mpdd 0 leif fxclone 3 5 60 SRR543720_step3.fx SRR543720_clone.fx SRR543720_step2.fx

leif mpdd 0 leif fxclone 3 5 60 SRR540284_step3.fx SRR540284_clone.fx SRR540284_step2.fx

leif mpdd 0 leif fxclone 3 5 60 SRR393523_step3.fx SRR393523_clone.fx SRR393523_step2.fx

leif mpdd 0 leif fxclone 3 5 60 SRR543722_step3.fx SRR543722_clone.fx SRR543722_step2.fx

leif mpdd 0 leif fxclone 3 5 60 SRR393528_step3.fx SRR393528_clone.fx SRR393528_step2.fx

leif mpdd 0 leif fxclone 3 5 60 SRR543725_step3.fx SRR543725_clone.fx SRR543725_step2.fx

leif mpdd 0 leif fxclone 3 5 60 SRR397731_step3.fx SRR397731_clone.fx SRR397731_step2.fx

leif mpdd 0 leif fxclone 3 5 60 SRR393516_step3.fx SRR393516_clone.fx SRR393516_step2.fx

leif mpdd 0 leif fxclone 3 5 60 SRR543728_step3.fx SRR543728_clone.fx SRR543728_step2.fx

leif mpdd 0 leif fxclone 3 5 60 SRR540282_step3.fx SRR540282_clone.fx SRR540282_step2.fx

leif mpdd 0 leif fxclone 3 5 60 SRR393519_step3.fx SRR393519_clone.fx SRR393519_step2.fx

leif mpdd 0 leif fxclone 3 5 60 SRR848045_step3.fx SRR848045_clone.fx SRR848045_step2.fx

leif mpdd 0 leif fxclone 3 5 60 SRR848046_step3.fx SRR848046_clone.fx SRR848046_step2.fx

leif mpdd 0 leif fxclone 3 5 60 SRR543724_step3.fx SRR543724_clone.fx SRR543724_step2.fx

leif mpdd 0 leif fxclone 3 5 60 SRR393529_step3.fx SRR393529_clone.fx SRR393529_step2.fx

leif mpdd 0 leif fxclone 3 5 60 SRR393522_step3.fx SRR393522_clone.fx SRR393522_step2.fx

leif mpdd 0 leif fxclone 3 5 60 SRR543730_step3.fx SRR543730_clone.fx SRR543730_step2.fx

leif mpdd 0 leif fxclone 3 5 60 SRR317500_step3.fx SRR317500_clone.fx SRR317500_step2.fx

leif mpdd 0 leif fxclone 3 5 60 SRR326122_step3.fx SRR326122_clone.fx SRR326122_step2.fx

leif mpdd 0 leif fxclone 3 5 60 SRR317501_step3.fx SRR317501_clone.fx SRR317501_step2.fx

leif mpdd 0 leif fxclone 3 5 60 SRR326077_step3.fx SRR326077_clone.fx SRR326077_step2.fx

leif mpdd 0 leif fxclone 3 5 60 SRR326090_step3.fx SRR326090_clone.fx SRR326090_step2.fx

leif mpdd 0 leif fxclone 3 5 60 SRR326091_step3.fx SRR326091_clone.fx SRR326091_step2.fx

leif mpdd 0 leif fxclone 3 5 60 SRR326092_step3.fx SRR326092_clone.fx SRR326092_step2.fx

leif mpdd 0 leif fxclone 3 5 60 SRR326095_step3.fx SRR326095_clone.fx SRR326095_step2.fx

leif mpdd 0 leif fxclone 3 5 60 SRR326097_step3.fx SRR326097_clone.fx SRR326097_step2.fx

leif mpdd 0 leif fxclone 3 5 60 SRR326114_step3.fx SRR326114_clone.fx SRR326114_step2.fx

leif mpdd 0 leif fxclone 3 5 60 SRR326118_step3.fx SRR326118_clone.fx SRR326118_step2.fx

leif mpdd 0 leif fxclone 3 5 60 SRR326121_step3.fx SRR326121_clone.fx SRR326121_step2.fx

leif mpdd 0 leif fxclone 3 5 60 SRR326125_step3.fx SRR326125_clone.fx SRR326125_step2.fx

leif mpdd 0 leif fxclone 3 5 60 SRR326129_step3.fx SRR326129_clone.fx SRR326129_step2.fx

leif mpdd 0 leif fxclone 3 5 60 SRR326132_step3.fx SRR326132_clone.fx SRR326132_step2.fx

leif mpdd 0 leif fxclone 3 5 60 SRR326142_step3.fx SRR326142_clone.fx SRR326142_step2.fx

leif mpdd 0 leif fxclone 3 5 60 SRR326143_step3.fx SRR326143_clone.fx SRR326143_step2.fx

leif mpdd 0 leif fxclone 3 5 60 SRR326150_step3.fx SRR326150_clone.fx SRR326150_step2.fx

leif mpdd 0 leif fxclone 3 5 60 SRR326152_step3.fx SRR326152_clone.fx SRR326152_step2.fx

leif mpdd 0 leif fxclone 3 5 60 SRR326153_step3.fx SRR326153_clone.fx SRR326153_step2.fx

leif mpdd 0 leif fxclone 3 5 60 SRR326154_step3.fx SRR326154_clone.fx SRR326154_step2.fx

leif mpdd 0 leif fxclone 3 5 60 SRR326079_step3.fx SRR326079_clone.fx SRR326079_step2.fx

leif mpdd 0 leif fxclone 3 5 60 SRR326080_step3.fx SRR326080_clone.fx SRR326080_step2.fx

leif mpdd 0 leif fxclone 3 5 60 SRR326106_step3.fx SRR326106_clone.fx SRR326106_step2.fx

leif mpdd 0 leif fxclone 3 5 60 SRR326111_step3.fx SRR326111_clone.fx SRR326111_step2.fx

leif mpdd 0 leif fxclone 3 5 60 SRR326115_step3.fx SRR326115_clone.fx SRR326115_step2.fx

leif mpdd 0 leif fxclone 3 5 60 SRR326124_step3.fx SRR326124_clone.fx SRR326124_step2.fx

leif mpdd 0 leif fxclone 3 5 60 SRR326126_step3.fx SRR326126_clone.fx SRR326126_step2.fx

leif mpdd 0 leif fxclone 3 5 60 SRR326127_step3.fx SRR326127_clone.fx SRR326127_step2.fx

leif mpdd 0 leif fxclone 3 5 60 SRR326131_step3.fx SRR326131_clone.fx SRR326131_step2.fx

leif mpdd 0 leif fxclone 3 5 60 SRR845282_step3.fx SRR845282_clone.fx SRR845282_step2.fx

leif mpdd 0 leif fxclone 3 5 60 SRR845283_step3.fx SRR845283_clone.fx SRR845283_step2.fx

leif mpdd 0 leif fxclone 3 5 60 SRR538771_step3.fx SRR538771_clone.fx SRR538771_step2.fx

leif mpdd 0 leif fxclone 3 5 60 SRR538775_step3.fx SRR538775_clone.fx SRR538775_step2.fx

leif mpdd 0 leif fxclone 3 5 60 SRR641732_step3.fx SRR641732_clone.fx SRR641732_step2.fx

leif mpdd 0 leif fxclone 3 5 60 SRR640891_step3.fx SRR640891_clone.fx SRR640891_step2.fx

leif mpdd 0 leif fxclone 3 5 60 SRR647106_step3.fx SRR647106_clone.fx SRR647106_step2.fx

leif mpdd 0 leif fxclone 3 5 60 SRR647101_step3.fx SRR647101_clone.fx SRR647101_step2.fx

leif mpdd 0 leif fxclone 3 5 60 SRR845263_step3.fx SRR845263_clone.fx SRR845263_step2.fx

leif mpdd 0 leif fxclone 3 5 60 SRR845264_step3.fx SRR845264_clone.fx SRR845264_step2.fx

leif mpdd 0 leif fxclone 3 5 60 SRR640895_step3.fx SRR640895_clone.fx SRR640895_step2.fx

leif mpdd 0 leif fxclone 3 5 60 SRR641731_step3.fx SRR641731_clone.fx SRR641731_step2.fx

leif mpdd 0 leif fxclone 3 5 60 SRR641730_step3.fx SRR641730_clone.fx SRR641730_step2.fx

leif mpdd 0 leif fxclone 3 5 60 SRR641728_step3.fx SRR641728_clone.fx SRR641728_step2.fx

leif mpdd 0 leif fxclone 3 5 60 SRR845261_step3.fx SRR845261_clone.fx SRR845261_step2.fx

leif mpdd 0 leif fxclone 3 5 60 SRR845262_step3.fx SRR845262_clone.fx SRR845262_step2.fx

leif mpdd 0 leif fxclone 3 5 60 SRR647109_step3.fx SRR647109_clone.fx SRR647109_step2.fx

leif mpdd 0 leif fxclone 3 5 60 SRR647103_step3.fx SRR647103_clone.fx SRR647103_step2.fx

leif mpdd 0 leif fxclone 3 5 60 SRR845182_step3.fx SRR845182_clone.fx SRR845182_step2.fx

leif mpdd 0 leif fxclone 3 5 60 SRR640890_step3.fx SRR640890_clone.fx SRR640890_step2.fx

leif mpdd 0 leif fxclone 3 5 60 SRR641735_step3.fx SRR641735_clone.fx SRR641735_step2.fx

leif mpdd 0 leif fxclone 3 5 60 SRR845181_step3.fx SRR845181_clone.fx SRR845181_step2.fx

leif mpdd 0 leif fxclone 3 5 60 SRR845137_step3.fx SRR845137_clone.fx SRR845137_step2.fx

leif mpdd 0 leif fxclone 3 5 60 SRR845136_step3.fx SRR845136_clone.fx SRR845136_step2.fx

leif mpdd 0 leif fxclone 3 5 60 SRR641729_step3.fx SRR641729_clone.fx SRR641729_step2.fx

leif mpdd 0 leif fxclone 3 5 60 SRR640892_step3.fx SRR640892_clone.fx SRR640892_step2.fx

leif mpdd 0 leif fxclone 3 5 60 SRR647108_step3.fx SRR647108_clone.fx SRR647108_step2.fx

leif mpdd 0 leif fxclone 3 5 60 SRR845179_step3.fx SRR845179_clone.fx SRR845179_step2.fx

leif mpdd 0 leif fxclone 3 5 60 SRR845180_step3.fx SRR845180_clone.fx SRR845180_step2.fx

leif mpdd 0 leif fxclone 3 5 60 SRR629744_step3.fx SRR629744_clone.fx SRR629744_step2.fx

leif mpdd 0 leif fxclone 3 5 60 SRR629743_step3.fx SRR629743_clone.fx SRR629743_step2.fx

leif mpdd 0 leif fxclone 3 5 60 SRR646259_step3.fx SRR646259_clone.fx SRR646259_step2.fx

leif mpdd 0 leif fxclone 3 5 60 SRR646260_step3.fx SRR646260_clone.fx SRR646260_step2.fx

leif mpdd 0 leif fxclone 3 5 60 SRR646258_step3.fx SRR646258_clone.fx SRR646258_step2.fx

leif mpdd 0 leif fxclone 3 5 60 SRR640897_step3.fx SRR640897_clone.fx SRR640897_step2.fx

leif mpdd 0 leif fxclone 3 5 60 SRR640896_step3.fx SRR640896_clone.fx SRR640896_step2.fx

leif mpdd 0 leif fxclone 3 5 60 SRR845203_step3.fx SRR845203_clone.fx SRR845203_step2.fx

leif mpdd 0 leif fxclone 3 5 60 SRR845204_step3.fx SRR845204_clone.fx SRR845204_step2.fx

leif mpdd 0 leif fxclone 3 5 60 SRR647105_step3.fx SRR647105_clone.fx SRR647105_step2.fx

leif mpdd 0 leif fxclone 3 5 60 SRR845269_step3.fx SRR845269_clone.fx SRR845269_step2.fx

leif mpdd 0 leif fxclone 3 5 60 SRR845272_step3.fx SRR845272_clone.fx SRR845272_step2.fx

leif mpdd 0 leif fxclone 3 5 60 SRR647102_step3.fx SRR647102_clone.fx SRR647102_step2.fx

leif mpdd 0 leif fxclone 3 5 60 SRR641734_step3.fx SRR641734_clone.fx SRR641734_step2.fx

leif mpdd 0 leif fxclone 3 5 60 SRR641733_step3.fx SRR641733_clone.fx SRR641733_step2.fx

leif mpdd 0 leif fxclone 3 5 60 SRR538776_step3.fx SRR538776_clone.fx SRR538776_step2.fx

leif mpdd 0 leif fxclone 3 5 60 SRR538773_step3.fx SRR538773_clone.fx SRR538773_step2.fx

leif mpdd 0 leif fxclone 3 5 60 SRR538779_step3.fx SRR538779_clone.fx SRR538779_step2.fx

leif mpdd 0 leif fxclone 3 5 60 SRR538780_step3.fx SRR538780_clone.fx SRR538780_step2.fx

leif mpdd 0 leif fxclone 3 5 60 SRR641727_step3.fx SRR641727_clone.fx SRR641727_step2.fx

leif mpdd 0 leif fxclone 3 5 60 SRR640893_step3.fx SRR640893_clone.fx SRR640893_step2.fx

leif mpdd 0 leif fxclone 3 5 60 SRR647107_step3.fx SRR647107_clone.fx SRR647107_step2.fx

leif mpdd 0 leif fxclone 3 5 60 SRR845223_step3.fx SRR845223_clone.fx SRR845223_step2.fx

leif mpdd 0 leif fxclone 3 5 60 SRR845222_step3.fx SRR845222_clone.fx SRR845222_step2.fx

leif mpdd 0 leif fxclone 3 5 60 SRR845177_step3.fx SRR845177_clone.fx SRR845177_step2.fx

leif mpdd 0 leif fxclone 3 5 60 SRR845178_step3.fx SRR845178_clone.fx SRR845178_step2.fx

leif mpdd 0 leif fxclone 3 5 60 SRR647104_step3.fx SRR647104_clone.fx SRR647104_step2.fx

leif mpdd 0 leif fxclone 3 5 60 SRR640894_step3.fx SRR640894_clone.fx SRR640894_step2.fx

leif mpdd 0 leif fxclone 3 5 60 SRR641726_step3.fx SRR641726_clone.fx SRR641726_step2.fx

leif mpdd 0 leif fxclone 3 5 60 SRR538777_step3.fx SRR538777_clone.fx SRR538777_step2.fx

leif mpdd 0 leif fxclone 3 5 60 SRR538772_step3.fx SRR538772_clone.fx SRR538772_step2.fx

leif mpdd 0 leif fxclone 3 5 60 SRR530262_step3.fx SRR530262_clone.fx SRR530262_step2.fx

leif mpdd 0 leif fxclone 3 5 60 SRR530263_step3.fx SRR530263_clone.fx SRR530263_step2.fx

leif mpdd 0

echo %date% %time%

:: Group overlapping read pairs into "contig-like groups".

leif mpdd 0 leif fxgroup SRR538787_step4.fx SRR538787_step3.fx

leif mpdd 0 leif fxgroup SRR538782_step4.fx SRR538782_step3.fx

leif mpdd 0 leif fxgroup SRR538784_step4.fx SRR538784_step3.fx

leif mpdd 0 leif fxgroup SRR538788_step4.fx SRR538788_step3.fx

leif mpdd 0 leif fxgroup SRR538786_step4.fx SRR538786_step3.fx

leif mpdd 0 leif fxgroup SRR538774_step4.fx SRR538774_step3.fx

leif mpdd 0 leif fxgroup SRR538778_step4.fx SRR538778_step3.fx

leif mpdd 0 leif fxgroup SRR538781_step4.fx SRR538781_step3.fx

leif mpdd 0 leif fxgroup SRR538783_step4.fx SRR538783_step3.fx

leif mpdd 0 leif fxgroup SRR393530_step4.fx SRR393530_step3.fx

leif mpdd 0 leif fxgroup SRR543721_step4.fx SRR543721_step3.fx

leif mpdd 0 leif fxgroup SRR543723_step4.fx SRR543723_step3.fx

leif mpdd 0 leif fxgroup SRR393524_step4.fx SRR393524_step3.fx

leif mpdd 0 leif fxgroup SRR392813_step4.fx SRR392813_step3.fx

leif mpdd 0 leif fxgroup SRR630037_step4.fx SRR630037_step3.fx

leif mpdd 0 leif fxgroup SRR393526_step4.fx SRR393526_step3.fx

leif mpdd 0 leif fxgroup SRR540285_step4.fx SRR540285_step3.fx

leif mpdd 0 leif fxgroup SRR540283_step4.fx SRR540283_step3.fx

leif mpdd 0 leif fxgroup SRR393518_step4.fx SRR393518_step3.fx

leif mpdd 0 leif fxgroup SRR543729_step4.fx SRR543729_step3.fx

leif mpdd 0 leif fxgroup SRR393525_step4.fx SRR393525_step3.fx

leif mpdd 0 leif fxgroup SRR540281_step4.fx SRR540281_step3.fx

leif mpdd 0 leif fxgroup SRR393517_step4.fx SRR393517_step3.fx

leif mpdd 0 leif fxgroup SRR393527_step4.fx SRR393527_step3.fx

leif mpdd 0 leif fxgroup SRR543731_step4.fx SRR543731_step3.fx

leif mpdd 0 leif fxgroup SRR392814_step4.fx SRR392814_step3.fx

leif mpdd 0 leif fxgroup SRR393514_step4.fx SRR393514_step3.fx

leif mpdd 0 leif fxgroup SRR543727_step4.fx SRR543727_step3.fx

leif mpdd 0 leif fxgroup SRR393515_step4.fx SRR393515_step3.fx

leif mpdd 0 leif fxgroup SRR540280_step4.fx SRR540280_step3.fx

leif mpdd 0 leif fxgroup SRR543726_step4.fx SRR543726_step3.fx

leif mpdd 0 leif fxgroup SRR393520_step4.fx SRR393520_step3.fx

leif mpdd 0 leif fxgroup SRR393521_step4.fx SRR393521_step3.fx

leif mpdd 0 leif fxgroup SRR543720_step4.fx SRR543720_step3.fx

leif mpdd 0 leif fxgroup SRR540284_step4.fx SRR540284_step3.fx

leif mpdd 0 leif fxgroup SRR393523_step4.fx SRR393523_step3.fx

leif mpdd 0 leif fxgroup SRR543722_step4.fx SRR543722_step3.fx

leif mpdd 0 leif fxgroup SRR393528_step4.fx SRR393528_step3.fx

leif mpdd 0 leif fxgroup SRR543725_step4.fx SRR543725_step3.fx

leif mpdd 0 leif fxgroup SRR397731_step4.fx SRR397731_step3.fx

leif mpdd 0 leif fxgroup SRR393516_step4.fx SRR393516_step3.fx

leif mpdd 0 leif fxgroup SRR543728_step4.fx SRR543728_step3.fx

leif mpdd 0 leif fxgroup SRR540282_step4.fx SRR540282_step3.fx

leif mpdd 0 leif fxgroup SRR393519_step4.fx SRR393519_step3.fx

leif mpdd 0 leif fxgroup SRR848045_step4.fx SRR848045_step3.fx

leif mpdd 0 leif fxgroup SRR848046_step4.fx SRR848046_step3.fx

leif mpdd 0 leif fxgroup SRR543724_step4.fx SRR543724_step3.fx

leif mpdd 0 leif fxgroup SRR393529_step4.fx SRR393529_step3.fx

leif mpdd 0 leif fxgroup SRR393522_step4.fx SRR393522_step3.fx

leif mpdd 0 leif fxgroup SRR543730_step4.fx SRR543730_step3.fx

leif mpdd 0 leif fxgroup SRR317500_step4.fx SRR317500_step3.fx

leif mpdd 0 leif fxgroup SRR326122_step4.fx SRR326122_step3.fx

leif mpdd 0 leif fxgroup SRR317501_step4.fx SRR317501_step3.fx

leif mpdd 0 leif fxgroup SRR326077_step4.fx SRR326077_step3.fx

leif mpdd 0 leif fxgroup SRR326090_step4.fx SRR326090_step3.fx

leif mpdd 0 leif fxgroup SRR326091_step4.fx SRR326091_step3.fx

leif mpdd 0 leif fxgroup SRR326092_step4.fx SRR326092_step3.fx

leif mpdd 0 leif fxgroup SRR326095_step4.fx SRR326095_step3.fx

leif mpdd 0 leif fxgroup SRR326097_step4.fx SRR326097_step3.fx

leif mpdd 0 leif fxgroup SRR326114_step4.fx SRR326114_step3.fx

leif mpdd 0 leif fxgroup SRR326118_step4.fx SRR326118_step3.fx

leif mpdd 0 leif fxgroup SRR326121_step4.fx SRR326121_step3.fx

leif mpdd 0 leif fxgroup SRR326125_step4.fx SRR326125_step3.fx

leif mpdd 0 leif fxgroup SRR326129_step4.fx SRR326129_step3.fx

leif mpdd 0 leif fxgroup SRR326132_step4.fx SRR326132_step3.fx

leif mpdd 0 leif fxgroup SRR326142_step4.fx SRR326142_step3.fx

leif mpdd 0 leif fxgroup SRR326143_step4.fx SRR326143_step3.fx

leif mpdd 0 leif fxgroup SRR326150_step4.fx SRR326150_step3.fx

leif mpdd 0 leif fxgroup SRR326152_step4.fx SRR326152_step3.fx

leif mpdd 0 leif fxgroup SRR326153_step4.fx SRR326153_step3.fx

leif mpdd 0 leif fxgroup SRR326154_step4.fx SRR326154_step3.fx

leif mpdd 0 leif fxgroup SRR326079_step4.fx SRR326079_step3.fx

leif mpdd 0 leif fxgroup SRR326080_step4.fx SRR326080_step3.fx

leif mpdd 0 leif fxgroup SRR326106_step4.fx SRR326106_step3.fx

leif mpdd 0 leif fxgroup SRR326111_step4.fx SRR326111_step3.fx

leif mpdd 0 leif fxgroup SRR326115_step4.fx SRR326115_step3.fx

leif mpdd 0 leif fxgroup SRR326124_step4.fx SRR326124_step3.fx

leif mpdd 0 leif fxgroup SRR326126_step4.fx SRR326126_step3.fx

leif mpdd 0 leif fxgroup SRR326127_step4.fx SRR326127_step3.fx

leif mpdd 0 leif fxgroup SRR326131_step4.fx SRR326131_step3.fx

leif mpdd 0 leif fxgroup SRR845282_step4.fx SRR845282_step3.fx

leif mpdd 0 leif fxgroup SRR845283_step4.fx SRR845283_step3.fx

leif mpdd 0 leif fxgroup SRR538771_step4.fx SRR538771_step3.fx

leif mpdd 0 leif fxgroup SRR538775_step4.fx SRR538775_step3.fx

leif mpdd 0 leif fxgroup SRR641732_step4.fx SRR641732_step3.fx

leif mpdd 0 leif fxgroup SRR640891_step4.fx SRR640891_step3.fx

leif mpdd 0 leif fxgroup SRR647106_step4.fx SRR647106_step3.fx

leif mpdd 0 leif fxgroup SRR647101_step4.fx SRR647101_step3.fx

leif mpdd 0 leif fxgroup SRR845263_step4.fx SRR845263_step3.fx

leif mpdd 0 leif fxgroup SRR845264_step4.fx SRR845264_step3.fx

leif mpdd 0 leif fxgroup SRR640895_step4.fx SRR640895_step3.fx

leif mpdd 0 leif fxgroup SRR641731_step4.fx SRR641731_step3.fx

leif mpdd 0 leif fxgroup SRR641730_step4.fx SRR641730_step3.fx

leif mpdd 0 leif fxgroup SRR641728_step4.fx SRR641728_step3.fx

leif mpdd 0 leif fxgroup SRR845261_step4.fx SRR845261_step3.fx

leif mpdd 0 leif fxgroup SRR845262_step4.fx SRR845262_step3.fx

leif mpdd 0 leif fxgroup SRR647109_step4.fx SRR647109_step3.fx

leif mpdd 0 leif fxgroup SRR647103_step4.fx SRR647103_step3.fx

leif mpdd 0 leif fxgroup SRR845182_step4.fx SRR845182_step3.fx

leif mpdd 0 leif fxgroup SRR640890_step4.fx SRR640890_step3.fx

leif mpdd 0 leif fxgroup SRR641735_step4.fx SRR641735_step3.fx

leif mpdd 0 leif fxgroup SRR845181_step4.fx SRR845181_step3.fx

leif mpdd 0 leif fxgroup SRR845137_step4.fx SRR845137_step3.fx

leif mpdd 0 leif fxgroup SRR845136_step4.fx SRR845136_step3.fx

leif mpdd 0 leif fxgroup SRR641729_step4.fx SRR641729_step3.fx

leif mpdd 0 leif fxgroup SRR640892_step4.fx SRR640892_step3.fx

leif mpdd 0 leif fxgroup SRR647108_step4.fx SRR647108_step3.fx

leif mpdd 0 leif fxgroup SRR845179_step4.fx SRR845179_step3.fx

leif mpdd 0 leif fxgroup SRR845180_step4.fx SRR845180_step3.fx

leif mpdd 0 leif fxgroup SRR629744_step4.fx SRR629744_step3.fx

leif mpdd 0 leif fxgroup SRR629743_step4.fx SRR629743_step3.fx

leif mpdd 0 leif fxgroup SRR646259_step4.fx SRR646259_step3.fx

leif mpdd 0 leif fxgroup SRR646260_step4.fx SRR646260_step3.fx

leif mpdd 0 leif fxgroup SRR646258_step4.fx SRR646258_step3.fx

leif mpdd 0 leif fxgroup SRR640897_step4.fx SRR640897_step3.fx

leif mpdd 0 leif fxgroup SRR640896_step4.fx SRR640896_step3.fx

leif mpdd 0 leif fxgroup SRR845203_step4.fx SRR845203_step3.fx

leif mpdd 0 leif fxgroup SRR845204_step4.fx SRR845204_step3.fx

leif mpdd 0 leif fxgroup SRR647105_step4.fx SRR647105_step3.fx

leif mpdd 0 leif fxgroup SRR845269_step4.fx SRR845269_step3.fx

leif mpdd 0 leif fxgroup SRR845272_step4.fx SRR845272_step3.fx

leif mpdd 0 leif fxgroup SRR647102_step4.fx SRR647102_step3.fx

leif mpdd 0 leif fxgroup SRR641734_step4.fx SRR641734_step3.fx

leif mpdd 0 leif fxgroup SRR641733_step4.fx SRR641733_step3.fx

leif mpdd 0 leif fxgroup SRR538776_step4.fx SRR538776_step3.fx

leif mpdd 0 leif fxgroup SRR538773_step4.fx SRR538773_step3.fx

leif mpdd 0 leif fxgroup SRR538779_step4.fx SRR538779_step3.fx

leif mpdd 0 leif fxgroup SRR538780_step4.fx SRR538780_step3.fx

leif mpdd 0 leif fxgroup SRR641727_step4.fx SRR641727_step3.fx

leif mpdd 0 leif fxgroup SRR640893_step4.fx SRR640893_step3.fx

leif mpdd 0 leif fxgroup SRR647107_step4.fx SRR647107_step3.fx

leif mpdd 0 leif fxgroup SRR845223_step4.fx SRR845223_step3.fx

leif mpdd 0 leif fxgroup SRR845222_step4.fx SRR845222_step3.fx

leif mpdd 0 leif fxgroup SRR845177_step4.fx SRR845177_step3.fx

leif mpdd 0 leif fxgroup SRR845178_step4.fx SRR845178_step3.fx

leif mpdd 0 leif fxgroup SRR647104_step4.fx SRR647104_step3.fx

leif mpdd 0 leif fxgroup SRR640894_step4.fx SRR640894_step3.fx

leif mpdd 0 leif fxgroup SRR641726_step4.fx SRR641726_step3.fx

leif mpdd 0 leif fxgroup SRR538777_step4.fx SRR538777_step3.fx

leif mpdd 0 leif fxgroup SRR538772_step4.fx SRR538772_step3.fx

leif mpdd 0 leif fxgroup SRR530262_step4.fx SRR530262_step3.fx

leif mpdd 0 leif fxgroup SRR530263_step4.fx SRR530263_step3.fx

leif mpdd 0

echo %date% %time%

:: Sample read pairs from each "contig-like group".

leif mpdd 0 leif fxsample 0 1 SRR538787_step5.fxa SRR538787_step4.fx

leif mpdd 0 leif fxsample 0 1 SRR538782_step5.fxa SRR538782_step4.fx

leif mpdd 0 leif fxsample 0 1 SRR538784_step5.fxa SRR538784_step4.fx

leif mpdd 0 leif fxsample 0 1 SRR538788_step5.fxa SRR538788_step4.fx

leif mpdd 0 leif fxsample 0 1 SRR538786_step5.fxa SRR538786_step4.fx

leif mpdd 0 leif fxsample 0 1 SRR538774_step5.fxa SRR538774_step4.fx

leif mpdd 0 leif fxsample 0 1 SRR538778_step5.fxa SRR538778_step4.fx

leif mpdd 0 leif fxsample 0 1 SRR538781_step5.fxa SRR538781_step4.fx

leif mpdd 0 leif fxsample 0 1 SRR538783_step5.fxa SRR538783_step4.fx

leif mpdd 0 leif fxsample 0 1 SRR393530_step5.fxa SRR393530_step4.fx

leif mpdd 0 leif fxsample 0 1 SRR543721_step5.fxa SRR543721_step4.fx

leif mpdd 0 leif fxsample 0 1 SRR543723_step5.fxa SRR543723_step4.fx

leif mpdd 0 leif fxsample 0 1 SRR393524_step5.fxa SRR393524_step4.fx

leif mpdd 0 leif fxsample 0 1 SRR392813_step5.fxa SRR392813_step4.fx

leif mpdd 0 leif fxsample 0 1 SRR630037_step5.fxa SRR630037_step4.fx

leif mpdd 0 leif fxsample 0 1 SRR393526_step5.fxa SRR393526_step4.fx

leif mpdd 0 leif fxsample 0 1 SRR540285_step5.fxa SRR540285_step4.fx

leif mpdd 0 leif fxsample 0 1 SRR540283_step5.fxa SRR540283_step4.fx

leif mpdd 0 leif fxsample 0 1 SRR393518_step5.fxa SRR393518_step4.fx

leif mpdd 0 leif fxsample 0 1 SRR543729_step5.fxa SRR543729_step4.fx

leif mpdd 0 leif fxsample 0 1 SRR393525_step5.fxa SRR393525_step4.fx

leif mpdd 0 leif fxsample 0 1 SRR540281_step5.fxa SRR540281_step4.fx

leif mpdd 0 leif fxsample 0 1 SRR393517_step5.fxa SRR393517_step4.fx

leif mpdd 0 leif fxsample 0 1 SRR393527_step5.fxa SRR393527_step4.fx

leif mpdd 0 leif fxsample 0 1 SRR543731_step5.fxa SRR543731_step4.fx

leif mpdd 0 leif fxsample 0 1 SRR392814_step5.fxa SRR392814_step4.fx

leif mpdd 0 leif fxsample 0 1 SRR393514_step5.fxa SRR393514_step4.fx

leif mpdd 0 leif fxsample 0 1 SRR543727_step5.fxa SRR543727_step4.fx

leif mpdd 0 leif fxsample 0 1 SRR393515_step5.fxa SRR393515_step4.fx

leif mpdd 0 leif fxsample 0 1 SRR540280_step5.fxa SRR540280_step4.fx

leif mpdd 0 leif fxsample 0 1 SRR543726_step5.fxa SRR543726_step4.fx

leif mpdd 0 leif fxsample 0 1 SRR393520_step5.fxa SRR393520_step4.fx

leif mpdd 0 leif fxsample 0 1 SRR393521_step5.fxa SRR393521_step4.fx

leif mpdd 0 leif fxsample 0 1 SRR543720_step5.fxa SRR543720_step4.fx

leif mpdd 0 leif fxsample 0 1 SRR540284_step5.fxa SRR540284_step4.fx

leif mpdd 0 leif fxsample 0 1 SRR393523_step5.fxa SRR393523_step4.fx

leif mpdd 0 leif fxsample 0 1 SRR543722_step5.fxa SRR543722_step4.fx

leif mpdd 0 leif fxsample 0 1 SRR393528_step5.fxa SRR393528_step4.fx

leif mpdd 0 leif fxsample 0 1 SRR543725_step5.fxa SRR543725_step4.fx

leif mpdd 0 leif fxsample 0 1 SRR397731_step5.fxb SRR397731_step4.fx

leif mpdd 0 leif fxsample 0 1 SRR393516_step5.fxb SRR393516_step4.fx

leif mpdd 0 leif fxsample 0 1 SRR543728_step5.fxb SRR543728_step4.fx

leif mpdd 0 leif fxsample 0 1 SRR540282_step5.fxb SRR540282_step4.fx

leif mpdd 0 leif fxsample 0 1 SRR393519_step5.fxb SRR393519_step4.fx

leif mpdd 0 leif fxsample 0 1 SRR848045_step5.fxb SRR848045_step4.fx

leif mpdd 0 leif fxsample 0 1 SRR848046_step5.fxb SRR848046_step4.fx

leif mpdd 0 leif fxsample 0 1 SRR543724_step5.fxb SRR543724_step4.fx

leif mpdd 0 leif fxsample 0 1 SRR393529_step5.fxb SRR393529_step4.fx

leif mpdd 0 leif fxsample 0 1 SRR393522_step5.fxb SRR393522_step4.fx

leif mpdd 0 leif fxsample 0 1 SRR543730_step5.fxb SRR543730_step4.fx

leif mpdd 0 leif fxsample 0 1 SRR317500_step5.fxb SRR317500_step4.fx

leif mpdd 0 leif fxsample 0 1 SRR326122_step5.fxb SRR326122_step4.fx

leif mpdd 0 leif fxsample 0 1 SRR317501_step5.fxb SRR317501_step4.fx

leif mpdd 0 leif fxsample 0 1 SRR326077_step5.fxb SRR326077_step4.fx

leif mpdd 0 leif fxsample 0 1 SRR326090_step5.fxb SRR326090_step4.fx

leif mpdd 0 leif fxsample 0 1 SRR326091_step5.fxb SRR326091_step4.fx

leif mpdd 0 leif fxsample 0 1 SRR326092_step5.fxb SRR326092_step4.fx

leif mpdd 0 leif fxsample 0 1 SRR326095_step5.fxb SRR326095_step4.fx

leif mpdd 0 leif fxsample 0 1 SRR326097_step5.fxb SRR326097_step4.fx

leif mpdd 0 leif fxsample 0 1 SRR326114_step5.fxb SRR326114_step4.fx

leif mpdd 0 leif fxsample 0 1 SRR326118_step5.fxb SRR326118_step4.fx

leif mpdd 0 leif fxsample 0 1 SRR326121_step5.fxb SRR326121_step4.fx

leif mpdd 0 leif fxsample 0 1 SRR326125_step5.fxb SRR326125_step4.fx

leif mpdd 0 leif fxsample 0 1 SRR326129_step5.fxb SRR326129_step4.fx

leif mpdd 0 leif fxsample 0 1 SRR326132_step5.fxb SRR326132_step4.fx

leif mpdd 0 leif fxsample 0 1 SRR326142_step5.fxb SRR326142_step4.fx

leif mpdd 0 leif fxsample 0 1 SRR326143_step5.fxb SRR326143_step4.fx

leif mpdd 0 leif fxsample 0 1 SRR326150_step5.fxb SRR326150_step4.fx

leif mpdd 0 leif fxsample 0 1 SRR326152_step5.fxb SRR326152_step4.fx

leif mpdd 0 leif fxsample 0 1 SRR326153_step5.fxb SRR326153_step4.fx

leif mpdd 0 leif fxsample 0 1 SRR326154_step5.fxb SRR326154_step4.fx

leif mpdd 0 leif fxsample 0 1 SRR326079_step5.fxb SRR326079_step4.fx

leif mpdd 0 leif fxsample 0 1 SRR326080_step5.fxb SRR326080_step4.fx

leif mpdd 0 leif fxsample 0 1 SRR326106_step5.fxb SRR326106_step4.fx

leif mpdd 0 leif fxsample 0 1 SRR326111_step5.fxb SRR326111_step4.fx

leif mpdd 0 leif fxsample 0 1 SRR326115_step5.fxb SRR326115_step4.fx

leif mpdd 0 leif fxsample 0 1 SRR326124_step5.fxb SRR326124_step4.fx

leif mpdd 0 leif fxsample 0 1 SRR326126_step5.fxb SRR326126_step4.fx

leif mpdd 0 leif fxsample 0 1 SRR326127_step5.fxb SRR326127_step4.fx

leif mpdd 0 leif fxsample 0 1 SRR326131_step5.fxb SRR326131_step4.fx

leif mpdd 0 leif fxsample 0 1 SRR845282_step5.fxb SRR845282_step4.fx

leif mpdd 0 leif fxsample 0 1 SRR845283_step5.fxb SRR845283_step4.fx

leif mpdd 0 leif fxsample 0 1 SRR538771_step5.fxb SRR538771_step4.fx

leif mpdd 0 leif fxsample 0 1 SRR538775_step5.fxb SRR538775_step4.fx

leif mpdd 0 leif fxsample 0 1 SRR641732_step5.fxb SRR641732_step4.fx

leif mpdd 0 leif fxsample 0 1 SRR640891_step5.fxc SRR640891_step4.fx

leif mpdd 0 leif fxsample 0 1 SRR647106_step5.fxc SRR647106_step4.fx

leif mpdd 0 leif fxsample 0 1 SRR647101_step5.fxc SRR647101_step4.fx

leif mpdd 0 leif fxsample 0 1 SRR845263_step5.fxc SRR845263_step4.fx

leif mpdd 0 leif fxsample 0 1 SRR845264_step5.fxc SRR845264_step4.fx

leif mpdd 0 leif fxsample 0 1 SRR640895_step5.fxc SRR640895_step4.fx

leif mpdd 0 leif fxsample 0 1 SRR641731_step5.fxc SRR641731_step4.fx

leif mpdd 0 leif fxsample 0 1 SRR641730_step5.fxc SRR641730_step4.fx

leif mpdd 0 leif fxsample 0 1 SRR641728_step5.fxc SRR641728_step4.fx

leif mpdd 0 leif fxsample 0 1 SRR845261_step5.fxc SRR845261_step4.fx

leif mpdd 0 leif fxsample 0 1 SRR845262_step5.fxc SRR845262_step4.fx

leif mpdd 0 leif fxsample 0 1 SRR647109_step5.fxc SRR647109_step4.fx

leif mpdd 0 leif fxsample 0 1 SRR647103_step5.fxc SRR647103_step4.fx

leif mpdd 0 leif fxsample 0 1 SRR845182_step5.fxc SRR845182_step4.fx

leif mpdd 0 leif fxsample 0 1 SRR640890_step5.fxc SRR640890_step4.fx

leif mpdd 0 leif fxsample 0 1 SRR641735_step5.fxc SRR641735_step4.fx

leif mpdd 0 leif fxsample 0 1 SRR845181_step5.fxc SRR845181_step4.fx

leif mpdd 0 leif fxsample 0 1 SRR845137_step5.fxc SRR845137_step4.fx

leif mpdd 0 leif fxsample 0 1 SRR845136_step5.fxc SRR845136_step4.fx

leif mpdd 0 leif fxsample 0 1 SRR641729_step5.fxc SRR641729_step4.fx

leif mpdd 0 leif fxsample 0 1 SRR640892_step5.fxc SRR640892_step4.fx

leif mpdd 0 leif fxsample 0 1 SRR647108_step5.fxc SRR647108_step4.fx

leif mpdd 0 leif fxsample 0 1 SRR845179_step5.fxc SRR845179_step4.fx

leif mpdd 0 leif fxsample 0 1 SRR845180_step5.fxc SRR845180_step4.fx

leif mpdd 0 leif fxsample 0 1 SRR629744_step5.fxc SRR629744_step4.fx

leif mpdd 0 leif fxsample 0 1 SRR629743_step5.fxc SRR629743_step4.fx

leif mpdd 0 leif fxsample 0 1 SRR646259_step5.fxc SRR646259_step4.fx

leif mpdd 0 leif fxsample 0 1 SRR646260_step5.fxc SRR646260_step4.fx

leif mpdd 0 leif fxsample 0 1 SRR646258_step5.fxd SRR646258_step4.fx

leif mpdd 0 leif fxsample 0 1 SRR640897_step5.fxd SRR640897_step4.fx

leif mpdd 0 leif fxsample 0 1 SRR640896_step5.fxd SRR640896_step4.fx

leif mpdd 0 leif fxsample 0 1 SRR845203_step5.fxd SRR845203_step4.fx

leif mpdd 0 leif fxsample 0 1 SRR845204_step5.fxd SRR845204_step4.fx

leif mpdd 0 leif fxsample 0 1 SRR647105_step5.fxd SRR647105_step4.fx

leif mpdd 0 leif fxsample 0 1 SRR845269_step5.fxd SRR845269_step4.fx

leif mpdd 0 leif fxsample 0 1 SRR845272_step5.fxd SRR845272_step4.fx

leif mpdd 0 leif fxsample 0 1 SRR647102_step5.fxd SRR647102_step4.fx

leif mpdd 0 leif fxsample 0 1 SRR641734_step5.fxd SRR641734_step4.fx

leif mpdd 0 leif fxsample 0 1 SRR641733_step5.fxd SRR641733_step4.fx

leif mpdd 0 leif fxsample 0 1 SRR538776_step5.fxd SRR538776_step4.fx

leif mpdd 0 leif fxsample 0 1 SRR538773_step5.fxd SRR538773_step4.fx

leif mpdd 0 leif fxsample 0 1 SRR538779_step5.fxd SRR538779_step4.fx

leif mpdd 0 leif fxsample 0 1 SRR538780_step5.fxd SRR538780_step4.fx

leif mpdd 0 leif fxsample 0 1 SRR641727_step5.fxd SRR641727_step4.fx

leif mpdd 0 leif fxsample 0 1 SRR640893_step5.fxd SRR640893_step4.fx

leif mpdd 0 leif fxsample 0 1 SRR647107_step5.fxd SRR647107_step4.fx

leif mpdd 0 leif fxsample 0 1 SRR845223_step5.fxd SRR845223_step4.fx

leif mpdd 0 leif fxsample 0 1 SRR845222_step5.fxd SRR845222_step4.fx

leif mpdd 0 leif fxsample 0 1 SRR845177_step5.fxd SRR845177_step4.fx

leif mpdd 0 leif fxsample 0 1 SRR845178_step5.fxe SRR845178_step4.fx

leif mpdd 0 leif fxsample 0 1 SRR647104_step5.fxe SRR647104_step4.fx

leif mpdd 0 leif fxsample 0 1 SRR640894_step5.fxe SRR640894_step4.fx

leif mpdd 0 leif fxsample 0 1 SRR641726_step5.fxe SRR641726_step4.fx

leif mpdd 0 leif fxsample 0 1 SRR538777_step5.fxe SRR538777_step4.fx

leif mpdd 0 leif fxsample 0 1 SRR538772_step5.fxe SRR538772_step4.fx

leif mpdd 0 leif fxsample 0 1 SRR530262_step5.fxe SRR530262_step4.fx

leif mpdd 0 leif fxsample 0 1 SRR530263_step5.fxe SRR530263_step4.fx

leif mpdd 0

echo %date% %time%

:: Align to all four large NCBI BLAST databases (nt, human_genomic, other_genomic, wgs)

echo word_length = 15; > qblast_settings.txt

echo dust = 1; >> qblast_settings.txt

echo dual_align_pct = 98; >> qblast_settings.txt

echo num_genus = 4; >> qblast_settings.txt

echo num_species = 4; >> qblast_settings.txt

echo num_consensus = 12; >> qblast_settings.txt

echo score_taxid= 5475, // Candida >> qblast_settings.txt

echo 10841; // Microviridae (to catch Enterobacteria phage) >> qblast_settings.txt

echo // >> qblast_settings.txt

echo ignore="|AHJH01"; // Exclude Hammondia hammondi contaminated with Bradyrhizobium. >> qblast_settings.txt

echo ignore="|AGTT01"; // Exclude Pantholops hodgsonii contaminated with Bradyrhizobium. >> qblast_settings.txt

echo ignore="|KE11"; // Exclude Pantholops hodgsonii contaminated with Bradyrhizobium. >> qblast_settings.txt

echo ignore="|AUYS01"; // Exclude Melampsora pinitorqua contaminated with Bradyrhizobium. >> qblast_settings.txt

echo ignore="|ABPJ01"; // Exclude Mchenga conophoros contaminated with Bradyrhizobium. >> qblast_settings.txt

echo ignore="|AK276546.1";// Exclude Gryllus bimaculatus contaminated with E coli. >> qblast_settings.txt

echo ignore="|BADR02"; // Exclude Clonorchis sinensis contaminated with E coli. >> qblast_settings.txt

echo ignore="|CBMN01"; // Exclude Hordeum pubiflorum contaminated with Propionibacterium acnes. >> qblast_settings.txt

echo ignore="|AAHY01"; // Exclude Mus musculus contaminated with E coli. >> qblast_settings.txt

echo ignore="|CAJW01"; // Exclude Hordeum vulgare contaminated with E coli. >> qblast_settings.txt

echo ignore="|CAJX01"; // Exclude Hordeum vulgare contaminated with Ralstonia pickettii. >> qblast_settings.txt

echo ignore="|CAWI01"; // Exclude Adineta vaga contaminated with E coli. >> qblast_settings.txt

echo ignore="|NZ_AJHE02";// Retracted. >> qblast_settings.txt

echo ignore="|CACX01"; // Exclude Strongyloides ratti contaminated with E coli. >> qblast_settings.txt

echo ignore="|CH003510.1";// Exclude Homo sapiens contaminated with E coli. >> qblast_settings.txt

echo ignore="|AHIO01"; // Exclude Plutella xylostella contaminated with Salmonella enterica. >> qblast_settings.txt

echo // >> qblast_settings.txt

echo ignore=81077; // Exclude artificial sequences. >> qblast_settings.txt

echo ignore=12908; // Exclude unclassified sequences. >> qblast_settings.txt

echo cat0= others; // Prokaryotes and viruses. >> qblast_settings.txt

echo cat1= 2759; // Eukaryotes >> qblast_settings.txt

leif qblast qblast_settings.txt taxid.git blast_*.fa.gz *_step5.fxa

leif qblast qblast_settings.txt taxid.git blast_*.fa.gz *_step5.fxb

leif qblast qblast_settings.txt taxid.git blast_*.fa.gz *_step5.fxc

leif qblast qblast_settings.txt taxid.git blast_*.fa.gz *_step5.fxd

leif qblast qblast_settings.txt taxid.git blast_*.fa.gz *_step5.fxe

echo %date% %time%

:: Extract reads which align to Candida (taxid=5475) or phage (taxid=10841) to "cp.qb" file.

leif qbmajority single 70 50 SRR538787_cp.qb SRR538787_step6.qb SRR538787_step5.qb taxid.git Taxid 5475 10841

leif qbmajority single 70 50 SRR538782_cp.qb SRR538782_step6.qb SRR538782_step5.qb taxid.git Taxid 5475 10841

leif qbmajority single 70 50 SRR538784_cp.qb SRR538784_step6.qb SRR538784_step5.qb taxid.git Taxid 5475 10841

leif qbmajority single 70 50 SRR538788_cp.qb SRR538788_step6.qb SRR538788_step5.qb taxid.git Taxid 5475 10841

leif qbmajority single 70 50 SRR538786_cp.qb SRR538786_step6.qb SRR538786_step5.qb taxid.git Taxid 5475 10841

leif qbmajority single 70 50 SRR538774_cp.qb SRR538774_step6.qb SRR538774_step5.qb taxid.git Taxid 5475 10841

leif qbmajority single 70 50 SRR538778_cp.qb SRR538778_step6.qb SRR538778_step5.qb taxid.git Taxid 5475 10841

leif qbmajority single 70 50 SRR538781_cp.qb SRR538781_step6.qb SRR538781_step5.qb taxid.git Taxid 5475 10841

leif qbmajority single 70 50 SRR538783_cp.qb SRR538783_step6.qb SRR538783_step5.qb taxid.git Taxid 5475 10841

leif qbmajority single 70 50 SRR393530_cp.qb SRR393530_step6.qb SRR393530_step5.qb taxid.git Taxid 5475 10841

leif qbmajority single 70 50 SRR543721_cp.qb SRR543721_step6.qb SRR543721_step5.qb taxid.git Taxid 5475 10841

leif qbmajority single 70 50 SRR543723_cp.qb SRR543723_step6.qb SRR543723_step5.qb taxid.git Taxid 5475 10841

leif qbmajority single 70 50 SRR393524_cp.qb SRR393524_step6.qb SRR393524_step5.qb taxid.git Taxid 5475 10841

leif qbmajority single 70 50 SRR392813_cp.qb SRR392813_step6.qb SRR392813_step5.qb taxid.git Taxid 5475 10841

leif qbmajority single 70 50 SRR630037_cp.qb SRR630037_step6.qb SRR630037_step5.qb taxid.git Taxid 5475 10841

leif qbmajority single 70 50 SRR393526_cp.qb SRR393526_step6.qb SRR393526_step5.qb taxid.git Taxid 5475 10841

leif qbmajority single 70 50 SRR540285_cp.qb SRR540285_step6.qb SRR540285_step5.qb taxid.git Taxid 5475 10841

leif qbmajority single 70 50 SRR540283_cp.qb SRR540283_step6.qb SRR540283_step5.qb taxid.git Taxid 5475 10841

leif qbmajority single 70 50 SRR393518_cp.qb SRR393518_step6.qb SRR393518_step5.qb taxid.git Taxid 5475 10841

leif qbmajority single 70 50 SRR543729_cp.qb SRR543729_step6.qb SRR543729_step5.qb taxid.git Taxid 5475 10841

leif qbmajority single 70 50 SRR393525_cp.qb SRR393525_step6.qb SRR393525_step5.qb taxid.git Taxid 5475 10841

leif qbmajority single 70 50 SRR540281_cp.qb SRR540281_step6.qb SRR540281_step5.qb taxid.git Taxid 5475 10841

leif qbmajority single 70 50 SRR393517_cp.qb SRR393517_step6.qb SRR393517_step5.qb taxid.git Taxid 5475 10841

leif qbmajority single 70 50 SRR393527_cp.qb SRR393527_step6.qb SRR393527_step5.qb taxid.git Taxid 5475 10841

leif qbmajority single 70 50 SRR543731_cp.qb SRR543731_step6.qb SRR543731_step5.qb taxid.git Taxid 5475 10841

leif qbmajority single 70 50 SRR392814_cp.qb SRR392814_step6.qb SRR392814_step5.qb taxid.git Taxid 5475 10841

leif qbmajority single 70 50 SRR393514_cp.qb SRR393514_step6.qb SRR393514_step5.qb taxid.git Taxid 5475 10841

leif qbmajority single 70 50 SRR543727_cp.qb SRR543727_step6.qb SRR543727_step5.qb taxid.git Taxid 5475 10841

leif qbmajority single 70 50 SRR393515_cp.qb SRR393515_step6.qb SRR393515_step5.qb taxid.git Taxid 5475 10841

leif qbmajority single 70 50 SRR540280_cp.qb SRR540280_step6.qb SRR540280_step5.qb taxid.git Taxid 5475 10841

leif qbmajority single 70 50 SRR543726_cp.qb SRR543726_step6.qb SRR543726_step5.qb taxid.git Taxid 5475 10841

leif qbmajority single 70 50 SRR393520_cp.qb SRR393520_step6.qb SRR393520_step5.qb taxid.git Taxid 5475 10841

leif qbmajority single 70 50 SRR393521_cp.qb SRR393521_step6.qb SRR393521_step5.qb taxid.git Taxid 5475 10841

leif qbmajority single 70 50 SRR543720_cp.qb SRR543720_step6.qb SRR543720_step5.qb taxid.git Taxid 5475 10841

leif qbmajority single 70 50 SRR540284_cp.qb SRR540284_step6.qb SRR540284_step5.qb taxid.git Taxid 5475 10841

leif qbmajority single 70 50 SRR393523_cp.qb SRR393523_step6.qb SRR393523_step5.qb taxid.git Taxid 5475 10841

leif qbmajority single 70 50 SRR543722_cp.qb SRR543722_step6.qb SRR543722_step5.qb taxid.git Taxid 5475 10841

leif qbmajority single 70 50 SRR393528_cp.qb SRR393528_step6.qb SRR393528_step5.qb taxid.git Taxid 5475 10841

leif qbmajority single 70 50 SRR543725_cp.qb SRR543725_step6.qb SRR543725_step5.qb taxid.git Taxid 5475 10841

leif qbmajority single 70 50 SRR397731_cp.qb SRR397731_step6.qb SRR397731_step5.qb taxid.git Taxid 5475 10841

leif qbmajority single 70 50 SRR393516_cp.qb SRR393516_step6.qb SRR393516_step5.qb taxid.git Taxid 5475 10841

leif qbmajority single 70 50 SRR543728_cp.qb SRR543728_step6.qb SRR543728_step5.qb taxid.git Taxid 5475 10841

leif qbmajority single 70 50 SRR540282_cp.qb SRR540282_step6.qb SRR540282_step5.qb taxid.git Taxid 5475 10841

leif qbmajority single 70 50 SRR393519_cp.qb SRR393519_step6.qb SRR393519_step5.qb taxid.git Taxid 5475 10841

leif qbmajority single 70 50 SRR848045_cp.qb SRR848045_step6.qb SRR848045_step5.qb taxid.git Taxid 5475 10841

leif qbmajority single 70 50 SRR848046_cp.qb SRR848046_step6.qb SRR848046_step5.qb taxid.git Taxid 5475 10841

leif qbmajority single 70 50 SRR543724_cp.qb SRR543724_step6.qb SRR543724_step5.qb taxid.git Taxid 5475 10841

leif qbmajority single 70 50 SRR393529_cp.qb SRR393529_step6.qb SRR393529_step5.qb taxid.git Taxid 5475 10841

leif qbmajority single 70 50 SRR393522_cp.qb SRR393522_step6.qb SRR393522_step5.qb taxid.git Taxid 5475 10841

leif qbmajority single 70 50 SRR543730_cp.qb SRR543730_step6.qb SRR543730_step5.qb taxid.git Taxid 5475 10841

leif qbmajority single 70 50 SRR317500_cp.qb SRR317500_step6.qb SRR317500_step5.qb taxid.git Taxid 5475 10841

leif qbmajority single 70 50 SRR326122_cp.qb SRR326122_step6.qb SRR326122_step5.qb taxid.git Taxid 5475 10841

leif qbmajority single 70 50 SRR317501_cp.qb SRR317501_step6.qb SRR317501_step5.qb taxid.git Taxid 5475 10841

leif qbmajority single 70 50 SRR326077_cp.qb SRR326077_step6.qb SRR326077_step5.qb taxid.git Taxid 5475 10841

leif qbmajority single 70 50 SRR326090_cp.qb SRR326090_step6.qb SRR326090_step5.qb taxid.git Taxid 5475 10841

leif qbmajority single 70 50 SRR326091_cp.qb SRR326091_step6.qb SRR326091_step5.qb taxid.git Taxid 5475 10841

leif qbmajority single 70 50 SRR326092_cp.qb SRR326092_step6.qb SRR326092_step5.qb taxid.git Taxid 5475 10841

leif qbmajority single 70 50 SRR326095_cp.qb SRR326095_step6.qb SRR326095_step5.qb taxid.git Taxid 5475 10841

leif qbmajority single 70 50 SRR326097_cp.qb SRR326097_step6.qb SRR326097_step5.qb taxid.git Taxid 5475 10841

leif qbmajority single 70 50 SRR326114_cp.qb SRR326114_step6.qb SRR326114_step5.qb taxid.git Taxid 5475 10841

leif qbmajority single 70 50 SRR326118_cp.qb SRR326118_step6.qb SRR326118_step5.qb taxid.git Taxid 5475 10841

leif qbmajority single 70 50 SRR326121_cp.qb SRR326121_step6.qb SRR326121_step5.qb taxid.git Taxid 5475 10841

leif qbmajority single 70 50 SRR326125_cp.qb SRR326125_step6.qb SRR326125_step5.qb taxid.git Taxid 5475 10841

leif qbmajority single 70 50 SRR326129_cp.qb SRR326129_step6.qb SRR326129_step5.qb taxid.git Taxid 5475 10841

leif qbmajority single 70 50 SRR326132_cp.qb SRR326132_step6.qb SRR326132_step5.qb taxid.git Taxid 5475 10841

leif qbmajority single 70 50 SRR326142_cp.qb SRR326142_step6.qb SRR326142_step5.qb taxid.git Taxid 5475 10841

leif qbmajority single 70 50 SRR326143_cp.qb SRR326143_step6.qb SRR326143_step5.qb taxid.git Taxid 5475 10841

leif qbmajority single 70 50 SRR326150_cp.qb SRR326150_step6.qb SRR326150_step5.qb taxid.git Taxid 5475 10841

leif qbmajority single 70 50 SRR326152_cp.qb SRR326152_step6.qb SRR326152_step5.qb taxid.git Taxid 5475 10841

leif qbmajority single 70 50 SRR326153_cp.qb SRR326153_step6.qb SRR326153_step5.qb taxid.git Taxid 5475 10841

leif qbmajority single 70 50 SRR326154_cp.qb SRR326154_step6.qb SRR326154_step5.qb taxid.git Taxid 5475 10841

leif qbmajority single 70 50 SRR326079_cp.qb SRR326079_step6.qb SRR326079_step5.qb taxid.git Taxid 5475 10841

leif qbmajority single 70 50 SRR326080_cp.qb SRR326080_step6.qb SRR326080_step5.qb taxid.git Taxid 5475 10841

leif qbmajority single 70 50 SRR326106_cp.qb SRR326106_step6.qb SRR326106_step5.qb taxid.git Taxid 5475 10841

leif qbmajority single 70 50 SRR326111_cp.qb SRR326111_step6.qb SRR326111_step5.qb taxid.git Taxid 5475 10841

leif qbmajority single 70 50 SRR326115_cp.qb SRR326115_step6.qb SRR326115_step5.qb taxid.git Taxid 5475 10841

leif qbmajority single 70 50 SRR326124_cp.qb SRR326124_step6.qb SRR326124_step5.qb taxid.git Taxid 5475 10841

leif qbmajority single 70 50 SRR326126_cp.qb SRR326126_step6.qb SRR326126_step5.qb taxid.git Taxid 5475 10841

leif qbmajority single 70 50 SRR326127_cp.qb SRR326127_step6.qb SRR326127_step5.qb taxid.git Taxid 5475 10841

leif qbmajority single 70 50 SRR326131_cp.qb SRR326131_step6.qb SRR326131_step5.qb taxid.git Taxid 5475 10841

leif qbmajority single 70 50 SRR845282_cp.qb SRR845282_step6.qb SRR845282_step5.qb taxid.git Taxid 5475 10841

leif qbmajority single 70 50 SRR845283_cp.qb SRR845283_step6.qb SRR845283_step5.qb taxid.git Taxid 5475 10841

leif qbmajority single 70 50 SRR538771_cp.qb SRR538771_step6.qb SRR538771_step5.qb taxid.git Taxid 5475 10841

leif qbmajority single 70 50 SRR538775_cp.qb SRR538775_step6.qb SRR538775_step5.qb taxid.git Taxid 5475 10841

leif qbmajority single 70 50 SRR641732_cp.qb SRR641732_step6.qb SRR641732_step5.qb taxid.git Taxid 5475 10841

leif qbmajority single 70 50 SRR640891_cp.qb SRR640891_step6.qb SRR640891_step5.qb taxid.git Taxid 5475 10841

leif qbmajority single 70 50 SRR647106_cp.qb SRR647106_step6.qb SRR647106_step5.qb taxid.git Taxid 5475 10841

leif qbmajority single 70 50 SRR647101_cp.qb SRR647101_step6.qb SRR647101_step5.qb taxid.git Taxid 5475 10841

leif qbmajority single 70 50 SRR845263_cp.qb SRR845263_step6.qb SRR845263_step5.qb taxid.git Taxid 5475 10841

leif qbmajority single 70 50 SRR845264_cp.qb SRR845264_step6.qb SRR845264_step5.qb taxid.git Taxid 5475 10841

leif qbmajority single 70 50 SRR640895_cp.qb SRR640895_step6.qb SRR640895_step5.qb taxid.git Taxid 5475 10841

leif qbmajority single 70 50 SRR641731_cp.qb SRR641731_step6.qb SRR641731_step5.qb taxid.git Taxid 5475 10841

leif qbmajority single 70 50 SRR641730_cp.qb SRR641730_step6.qb SRR641730_step5.qb taxid.git Taxid 5475 10841

leif qbmajority single 70 50 SRR641728_cp.qb SRR641728_step6.qb SRR641728_step5.qb taxid.git Taxid 5475 10841

leif qbmajority single 70 50 SRR845261_cp.qb SRR845261_step6.qb SRR845261_step5.qb taxid.git Taxid 5475 10841

leif qbmajority single 70 50 SRR845262_cp.qb SRR845262_step6.qb SRR845262_step5.qb taxid.git Taxid 5475 10841

leif qbmajority single 70 50 SRR647109_cp.qb SRR647109_step6.qb SRR647109_step5.qb taxid.git Taxid 5475 10841

leif qbmajority single 70 50 SRR647103_cp.qb SRR647103_step6.qb SRR647103_step5.qb taxid.git Taxid 5475 10841

leif qbmajority single 70 50 SRR845182_cp.qb SRR845182_step6.qb SRR845182_step5.qb taxid.git Taxid 5475 10841

leif qbmajority single 70 50 SRR640890_cp.qb SRR640890_step6.qb SRR640890_step5.qb taxid.git Taxid 5475 10841

leif qbmajority single 70 50 SRR641735_cp.qb SRR641735_step6.qb SRR641735_step5.qb taxid.git Taxid 5475 10841

leif qbmajority single 70 50 SRR845181_cp.qb SRR845181_step6.qb SRR845181_step5.qb taxid.git Taxid 5475 10841

leif qbmajority single 70 50 SRR845137_cp.qb SRR845137_step6.qb SRR845137_step5.qb taxid.git Taxid 5475 10841

leif qbmajority single 70 50 SRR845136_cp.qb SRR845136_step6.qb SRR845136_step5.qb taxid.git Taxid 5475 10841

leif qbmajority single 70 50 SRR641729_cp.qb SRR641729_step6.qb SRR641729_step5.qb taxid.git Taxid 5475 10841

leif qbmajority single 70 50 SRR640892_cp.qb SRR640892_step6.qb SRR640892_step5.qb taxid.git Taxid 5475 10841

leif qbmajority single 70 50 SRR647108_cp.qb SRR647108_step6.qb SRR647108_step5.qb taxid.git Taxid 5475 10841

leif qbmajority single 70 50 SRR845179_cp.qb SRR845179_step6.qb SRR845179_step5.qb taxid.git Taxid 5475 10841

leif qbmajority single 70 50 SRR845180_cp.qb SRR845180_step6.qb SRR845180_step5.qb taxid.git Taxid 5475 10841

leif qbmajority single 70 50 SRR629744_cp.qb SRR629744_step6.qb SRR629744_step5.qb taxid.git Taxid 5475 10841

leif qbmajority single 70 50 SRR629743_cp.qb SRR629743_step6.qb SRR629743_step5.qb taxid.git Taxid 5475 10841

leif qbmajority single 70 50 SRR646259_cp.qb SRR646259_step6.qb SRR646259_step5.qb taxid.git Taxid 5475 10841

leif qbmajority single 70 50 SRR646260_cp.qb SRR646260_step6.qb SRR646260_step5.qb taxid.git Taxid 5475 10841

leif qbmajority single 70 50 SRR646258_cp.qb SRR646258_step6.qb SRR646258_step5.qb taxid.git Taxid 5475 10841

leif qbmajority single 70 50 SRR640897_cp.qb SRR640897_step6.qb SRR640897_step5.qb taxid.git Taxid 5475 10841

leif qbmajority single 70 50 SRR640896_cp.qb SRR640896_step6.qb SRR640896_step5.qb taxid.git Taxid 5475 10841

leif qbmajority single 70 50 SRR845203_cp.qb SRR845203_step6.qb SRR845203_step5.qb taxid.git Taxid 5475 10841

leif qbmajority single 70 50 SRR845204_cp.qb SRR845204_step6.qb SRR845204_step5.qb taxid.git Taxid 5475 10841

leif qbmajority single 70 50 SRR647105_cp.qb SRR647105_step6.qb SRR647105_step5.qb taxid.git Taxid 5475 10841

leif qbmajority single 70 50 SRR845269_cp.qb SRR845269_step6.qb SRR845269_step5.qb taxid.git Taxid 5475 10841

leif qbmajority single 70 50 SRR845272_cp.qb SRR845272_step6.qb SRR845272_step5.qb taxid.git Taxid 5475 10841

leif qbmajority single 70 50 SRR647102_cp.qb SRR647102_step6.qb SRR647102_step5.qb taxid.git Taxid 5475 10841

leif qbmajority single 70 50 SRR641734_cp.qb SRR641734_step6.qb SRR641734_step5.qb taxid.git Taxid 5475 10841

leif qbmajority single 70 50 SRR641733_cp.qb SRR641733_step6.qb SRR641733_step5.qb taxid.git Taxid 5475 10841

leif qbmajority single 70 50 SRR538776_cp.qb SRR538776_step6.qb SRR538776_step5.qb taxid.git Taxid 5475 10841

leif qbmajority single 70 50 SRR538773_cp.qb SRR538773_step6.qb SRR538773_step5.qb taxid.git Taxid 5475 10841

leif qbmajority single 70 50 SRR538779_cp.qb SRR538779_step6.qb SRR538779_step5.qb taxid.git Taxid 5475 10841

leif qbmajority single 70 50 SRR538780_cp.qb SRR538780_step6.qb SRR538780_step5.qb taxid.git Taxid 5475 10841

leif qbmajority single 70 50 SRR641727_cp.qb SRR641727_step6.qb SRR641727_step5.qb taxid.git Taxid 5475 10841

leif qbmajority single 70 50 SRR640893_cp.qb SRR640893_step6.qb SRR640893_step5.qb taxid.git Taxid 5475 10841

leif qbmajority single 70 50 SRR647107_cp.qb SRR647107_step6.qb SRR647107_step5.qb taxid.git Taxid 5475 10841

leif qbmajority single 70 50 SRR845223_cp.qb SRR845223_step6.qb SRR845223_step5.qb taxid.git Taxid 5475 10841

leif qbmajority single 70 50 SRR845222_cp.qb SRR845222_step6.qb SRR845222_step5.qb taxid.git Taxid 5475 10841

leif qbmajority single 70 50 SRR845177_cp.qb SRR845177_step6.qb SRR845177_step5.qb taxid.git Taxid 5475 10841

leif qbmajority single 70 50 SRR845178_cp.qb SRR845178_step6.qb SRR845178_step5.qb taxid.git Taxid 5475 10841

leif qbmajority single 70 50 SRR647104_cp.qb SRR647104_step6.qb SRR647104_step5.qb taxid.git Taxid 5475 10841

leif qbmajority single 70 50 SRR640894_cp.qb SRR640894_step6.qb SRR640894_step5.qb taxid.git Taxid 5475 10841

leif qbmajority single 70 50 SRR641726_cp.qb SRR641726_step6.qb SRR641726_step5.qb taxid.git Taxid 5475 10841

leif qbmajority single 70 50 SRR538777_cp.qb SRR538777_step6.qb SRR538777_step5.qb taxid.git Taxid 5475 10841

leif qbmajority single 70 50 SRR538772_cp.qb SRR538772_step6.qb SRR538772_step5.qb taxid.git Taxid 5475 10841

leif qbmajority single 70 50 SRR530262_cp.qb SRR530262_step6.qb SRR530262_step5.qb taxid.git Taxid 5475 10841

leif qbmajority single 70 50 SRR530263_cp.qb SRR530263_step6.qb SRR530263_step5.qb taxid.git Taxid 5475 10841

:: Output summary of contamination in a CSV file (Excel compatible).

leif qbconsensus single 90 5 consensus90_5_step6.csv *_step6.qb taxid.git

echo Finished on %date% at %time%
