# Supplementary material for: Common Contaminants in Next-Generation Sequencing That Hinder Discovery of Low-Abundance Microbes
Source: PLoS One. 2014 May 16;9(5):e97876. doi: 10.1371/journal.pone.0097876 (PMC4023998; doi:10.1371/journal.pone.0097876)
Supplement: Text S4 — Example of a specific alignment to Bradyrhizobium sp. DFCI-1 from an Illumina HiSeq 2000 run at the Baylor College of Medicine. (DOC) [file pone.0097876.s007.doc]

Text S4: Example of a specific alignment to *Bradyrhizobium sp. DFCI-1* from an Illumina HiSeq 2000 run at the Baylor College of Medicine. A read pair was extracted from the 1000 Genomes Project run labeled “SRR768303” and the qblast tool (Leif Microbiome Analyzer) was used to align to all sequences in the NCBI “nt”, “human_genomic”, “other_genomic” and “wgs” databases downloaded on October 12^th^ 2013. Note that the reverse complement of Mate B is shown in the alignment results.

| ****************************************************************************  ****** Example read pair from 1000 Genome Project run “SRR768303” ******  ****************************************************************************  **Mate A (in FASTQ format):**  **@SRR768303.1980508 HWI-ST1154:205:D1M9PACXX:7:1104:19145:26501 length=101**  **CCGGAAGCGGCGCATGGCGTGCGACGCTGCCGCACGTTCTGCCATTCGTCGGCATTTTGCTGGCCGCTGTGCTGCTGCCCTTCGTCAGCAATGACTACTGG**  **+SRR768303.1980508 HWI-ST1154:205:D1M9PACXX:7:1104:19145:26501 length=101**  **C@BFFFFFHHGHHJIJJFGGGIIIJIHHHFFFDDDDDDDDDDCDDDEECDDDDDDDDEC@CCC@BDDDD9CDCDCCDCAACCCDD?BDDDCCDDDDDCAC@**  **Mate B (in FASTQ format):**  **@SRR768303.1980508 HWI-ST1154:205:D1M9PACXX:7:1104:19145:26501 length=101**  **CACCGAGCGTCAGCAGCGCGACATAGCCGATCGCGAGATGACCGGCAAAGCCGACCACCAGATTCAGTCCCGACACCAGCACCCAGTAGATCGCCATCCGG**  **+SRR768303.1980508 HWI-ST1154:205:D1M9PACXX:7:1104:19145:26501 length=101**  **@@@FFFDDFHGFF@FGHIIIJIJIJGIIIIFAHIGDCCA;5>CDDBDBB?<<:BBDBDDDD?CCCCCCCC:>@B@<BDA89ABBDD::::@>AB9BBDD<5**  ****************************************************************************  ****** qblast alignment results for example read pair shown above ******  ****************************************************************************  **48222 -> 48122 "NCBI wgs database>gi|540137305|gb|AMFB01000023.1| Bradyrhizobium sp. DFCI-1"**  **Mate A: CCGGAAGCGGCGCATGGCGTGCGACGCTGCCGCACGTTCTGCCATTCGTCGGCATTTTGCTGGCCGCTGTGCTGCTGCCCTTCGTCAGCAATGACTACTGG**  **100% |||||||||||||||||||||||||||||||||||||||||||||||||||||||||||||||||||||||||||||||||||||||||||||||||||||**  **NCBI wgs:CGGAAGCGGCGCATGGCGTGCGACGCTGCCGCACGTTCTGCCATTCGTCGGCATTTTGCTGGCCGCTGTGCTGCTGCCCTTCGTCAGCAATGACTACTGGG**  **Best homology:**  **100%: Bradyrhizobium sp. DFCI-1(taxid:1230476)**  **56%: pseudomallei group(taxid:111527)**  **56%: Herbaspirillum sp. CF444(taxid:1144319)**  **55%: Burkholderia cepacia complex(taxid:87882)**  **Gap between mates: 16 bases**  **48107 -> 48007 "NCBI wgs database>gi|540137305|gb|AMFB01000023.1| Bradyrhizobium sp. DFCI-1"**  **Mate Brc:CCGGATGGCGATCTACTGGGTGCTGGTGTCGGGACTGAATCTGGTGGTCGGCTTTGCCGGTCATCTCGCGATCGGCTATGTCGCGCTGCTGACGCTCGGTG**  **100% |||||||||||||||||||||||||||||||||||||||||||||||||||||||||||||||||||||||||||||||||||||||||||||||||||||**  **NCBI wgs:CCGGATGGCGATCTACTGGGTGCTGGTGTCGGGACTGAATCTGGTGGTCGGCTTTGCCGGTCATCTCGCGATCGGCTATGTCGCGCTGCTGACGCTCGGTG**  **Best homology:**  **100%: Bradyrhizobium sp. DFCI-1(taxid:1230476)**  **93%: Bradyrhizobium elkanii(taxid:29448)**  **90%: Bradyrhizobium sp. Ai1a-2(taxid:196490)**  **89%: Bradyrhizobium sp. th.b2(taxid:172088)** |
| --- |
