# Supplementary material for: Common Contaminants in Next-Generation Sequencing That Hinder Discovery of Low-Abundance Microbes
Source: PLoS One. 2014 May 16;9(5):e97876. doi: 10.1371/journal.pone.0097876 (PMC4023998; doi:10.1371/journal.pone.0097876)
Supplement: Text S5 — Example of a specific alignment to Bradyrhizobium sp. DFCI-1 from an Illumina HiSeq 2000 run at the Sanger Center. (DOC) [file pone.0097876.s008.doc]

Text S5: Example of a specific alignment to *Bradyrhizobium sp. DFCI-1* from an Illumina HiSeq 2000 run at the Sanger Center. A read pair was extracted from the 1000 Genomes Project run labeled “ERR050083” and the qblast tool (Leif Microbiome Analyzer) was used to align to all sequences in the NCBI “nt”, “human_genomic”, “other_genomic” and “wgs” databases downloaded on October 12^th^ 2013. Note that the reverse complement of Mate B is shown in the alignment results.

| ****************************************************************************  ****** Example read pair from 1000 Genome Project run “ERR050083” ******  ****************************************************************************  **Mate A (in FASTQ format):**  **@ERR050083.66294004 HS18_6628:6:2307:12675:172459#4 length=100**  **AGAAGATCGAGGGCATCGGCGATCTCCGCGACGAGTCCGACCGCGACGGCTATCGCGTCGTGATCGAGCTGAAGCGCGAGGCGGTGCCTGACGTCGTGCT**  **+ERR050083.66294004 HS18_6628:6:2307:12675:172459#4 length=100**  **DCIHHGJKGJIKKLKKMMKKNLIJJIOLLLNMJKLLKLKHKLNMGKLMKKJIGJGJHGKLGJEKEGEGFGJEJLHDKIKIGEJJ@NHF>DGGG8EB>>>(**  **Mate B (in FASTQ format):**  **@ERR050083.66294004 HS18_6628:6:2307:12675:172459#4 length=100**  **AGTCGCGCGACATCAGCGTGTCGCGCGCCGTGTTCGGATCGGGAGAGGTCCGGATCACCCGGATCATCTCGTCGATATTGGCAACCGCGATGGCGAGGCC**  **+ERR050083.66294004 HS18_6628:6:2307:12675:172459#4 length=100**  **:FDGEFHFJJG>JMJFMKINHKKILKMJJHJIFLJFLJIIHHLHHGJI?JIAKJIGIHJLKEFBFGJJCLGH<ICJCGKE6I>K4LCFFIDDF:1@2?>6**  ****************************************************************************  ****** qblast alignment results for example read pair shown above ******  ****************************************************************************  **180734 -> 180833 "NCBI wgs database>** **gi|540139214|gb|AMFB01000008.1| Bradyrhizobium sp. DFCI-1"**  **Mate A: AGAAGATCGAGGGCATCGGCGATCTCCGCGACGAGTCCGACCGCGACGGCTATCGCGTCGTGATCGAGCTGAAGCGCGAGGCGGTGCCTGACGTCGTGCT**  **100% ||||||||||||||||||||||||||||||||||||||||||||||||||||||||||||||||||||||||||||||||||||||||||||||||||||**  **NCBI wgs:AGAAGATCGAGGGCATCGGCGATCTCCGCGACGAGTCCGACCGCGACGGCTATCGCGTCGTGATCGAGCTGAAGCGCGAGGCGGTGCCTGACGTCGTGCT**  **Best homology:**  **100%: Bradyrhizobium sp. DFCI-1(taxid:1230476)**  **93%: Bradyrhizobium sp. th.b2(taxid:172088)**  **92%: Bradyrhizobium elkanii(taxid:29448)**  **90%: Bradyrhizobium sp. YR681(taxid:1144344)**  **Gap between mates: 193 bases**  **181027 -> 181126 "NCBI wgs database>** **gi|540139214|gb|AMFB01000008.1| Bradyrhizobium sp. DFCI-1"**  **Mate Brc:GGCCTCGCCATCGCGGTTGCCAATATCGACGAGATGATCCGGGTGATCCGGACCTCTCCCGATCCGAACACGGCGCGCGACACGCTGATGTCGCGCGACT**  **100% ||||||||||||||||||||||||||||||||||||||||||||||||||||||||||||||||||||||||||||||||||||||||||||||||||||**  **NCBI wgs:GGCCTCGCCATCGCGGTTGCCAATATCGACGAGATGATCCGGGTGATCCGGACCTCTCCCGATCCGAACACGGCGCGCGACACGCTGATGTCGCGCGACT**  **Best homology:**  **100%: Bradyrhizobium sp. DFCI-1(taxid:1230476)**  **91%: Bradyrhizobium sp. ORS 278(taxid:114615)**  **90%: Bradyrhizobium sp. CCBAU 15615(taxid:1128179)**  **90%: Rhodopseudomonas sp. B29(taxid:95607)** |
| --- |
