# Supplementary material for: Common Contaminants in Next-Generation Sequencing That Hinder Discovery of Low-Abundance Microbes
Source: PLoS One. 2014 May 16;9(5):e97876. doi: 10.1371/journal.pone.0097876 (PMC4023998; doi:10.1371/journal.pone.0097876)
Supplement: Text S6 — Example of a very specific alignment to Bradyrhizobium sp. DFCI-1 from an Illumina HiSeq 2000 run at the Broad Institute. (DOC) [file pone.0097876.s009.doc]

Text S6: Example of a very specific alignment to *Bradyrhizobium sp. DFCI-1* from an Illumina HiSeq 2000 run at the Broad Institute. A read pair was extracted from the 1000 Genomes Project run labeled “SRR075006” and the qblast tool (Leif Microbiome Analyzer) was used to align to all sequences in the NCBI “nt”, “human_genomic”, “other_genomic” and “wgs” databases downloaded on October 12^th^ 2013. Note that the reverse complement of Mate B is shown in the alignment results.

| ****************************************************************************  ****** Example read pair from 1000 Genome Project run “SRR075006” ******  ****************************************************************************  **Mate A (in FASTQ format):**  **@SRR075006.13530729 20A2FABXX101115:4:7:7368:200372 length=101**  **ACGGAGATTTCTTCAGGAGAGCCAAGCGCTATATTACAGATCCAATAGAAGGATCTATCGTATTCTCACTTCAATTCTATTCGTATTTGGAAAGACAAGAA**  **+SRR075006.13530729 20A2FABXX101115:4:7:7368:200372 length=101**  **HHHHHHHHHHHHHHFHGHHFHFHHEHHHHHHHHHGGHHHHHHHHHHHDHCHHFFH?EEEFEBHDCFBEDECDFDFHHHHHHHFEFB?C<EEF9EEEBC4E?**  **Mate B (in FASTQ format):**  **@SRR075006.13530729 20A2FABXX101115:4:7:7368:200372 length=101**  **TAGTTGATCAGCTGGTTAGTAACGAAGCCTTCTCCGTAGCTAAACTCGACGCTCTCGAACCGAGATATGATCTGTGGAGAAAGTAGAATCACCGACAACAA**  **+SRR075006.13530729 20A2FABXX101115:4:7:7368:200372 length=101**  **HHFHHHHHHHGHHHHHHHHFHHFHHHHHHHHHHHHHHHHHHFHAHHHDHFHHGBEHHHDHHHFFD9EDGDEH;EFHEEFDFD=6B5@C5?BCFFCEAA?FB**  ****************************************************************************  ****** qblast alignment results for example read pair shown above ******  ****************************************************************************  **25378 -> 25478 "NCBI wgs database>gi|540134119|gb|AMFB01000060.1| Bradyrhizobium sp. DFCI-1"**  **Mate A: ACGGAGATTTCTTCAGGAGAGCCAAGCGCTATATTACAGATCCAATAGAAGGATCTATCGTATTCTCACTTCAATTCTATTCGTATTTGGAAAGACAAGAA**  **100% |||||||||||||||||||||||||||||||||||||||||||||||||||||||||||||||||||||||||||||||||||||||||||||||||||||**  **NCBI wgs:ACGGAGATTTCTTCAGGAGAGCCAAGCGCTATATTACAGATCCAATAGAAGGATCTATCGTATTCTCACTTCAATTCTATTCGTATTTGGAAAGACAAGAA**  **Best homology:**  **100%: Bradyrhizobium sp. DFCI-1(taxid:1230476)**  **53%: Biomphalaria glabrata(taxid:6526)**  **53%: Bacillus cereus group(taxid:86661)**  **52%: Myotis lucifugus(taxid:59463)**  **Gap between mates: 129 bases**  **25608 -> 25708 "NCBI wgs database>gi|540134119|gb|AMFB01000060.1| Bradyrhizobium sp. DFCI-1"**  **Mate Brc:TTGTTGTCGGTGATTCTACTTTCTCCACAGATCATATCTCGGTTCGAGAGCGTCGAGTTTAGCTACGGAGAAGGCTTCGTTACTAACCAGCTGATCAACTA**  **100% |||||||||||||||||||||||||||||||||||||||||||||||||||||||||||||||||||||||||||||||||||||||||||||||||||||**  **NCBI wgs:TTGTTGTCGGTGATTCTACTTTCTCCACAGATCATATCTCGGTTCGAGAGCGTCGAGTTTAGCTACGGAGAAGGCTTCGTTACTAACCAGCTGATCAACTA**  **Best homology:**  **100%: Bradyrhizobium sp. DFCI-1(taxid:1230476)**  **53%: Bos grunniens(taxid:30521)**  **52%: Bubalus bubalis(taxid:89462)**  **52%: Dicentrarchus labrax(taxid:13489)** |
| --- |
