# Supplementary material for: Common Contaminants in Next-Generation Sequencing That Hinder Discovery of Low-Abundance Microbes
Source: PLoS One. 2014 May 16;9(5):e97876. doi: 10.1371/journal.pone.0097876 (PMC4023998; doi:10.1371/journal.pone.0097876)
Supplement: Text S7 — Example of a less specific alignment to Bradyrhizobium sp. DFCI-1 from an Illumina HiSeq 2000 run at the Max Planck Institute for Molecular Genetics. (DOC) [file pone.0097876.s010.doc]

Text S7: Example of a less specific alignment to *Bradyrhizobium sp. DFCI-1* from an Illumina HiSeq 2000 run at the Max Planck Institute for Molecular Genetics. A read pair was extracted from the 1000 Genomes Project run labeled “ERR239334” and the qblast tool (Leif Microbiome Analyzer) was used to align to all sequences in the NCBI “nt”, “human_genomic”, “other_genomic” and “wgs” databases downloaded on October 12^th^ 2013. Note that the reverse complement of Mate B is shown in the alignment results.

| ****************************************************************************  ****** Example read pair from 1000 Genome Project run “ERR239334” ******  ****************************************************************************  **Mate A (in FASTQ format):**  **@ERR239334.4529122 HWI-ST935:142:D1HB8ACXX:1:1105:17273:79035 length=101**  **GCGAATCGAATACTTTCAGCTTCACCGGCACGTCCTTGCCGTCGAGCGTGACGGCGTCGATATCGGCCTCGAGCTCGCGGAGCAGCGCGTTGTGCTTGTCG**  **+ERR239334.4529122 HWI-ST935:142:D1HB8ACXX:1:1105:17273:79035 length=101**  **BCCFFFFFHFHHGIJIHII>HIHEGIJI8CGHGIHIIJJJJIGGGIIE5=?DECBD;@@<BDDCDDDDDD@>BDB:>B@>>B<<A85>B0<B??@@ACBC@**  **Mate B (in FASTQ format):**  **@ERR239334.4529122 HWI-ST935:142:D1HB8ACXX:1:1105:17273:79035 length=101**  **CTCTCCATCATGAACATGCCGCCGCTGCCTTATCTCAAGCGCATCCCGGCGCTTGCGGAGATGGATCTCGAGGAGGCCTACACCAACGCCCACATCTGGGA**  **+ERR239334.4529122 HWI-ST935:142:D1HB8ACXX:1:1105:17273:79035 length=101**  **CCCFFFFFHHHHHJIJJJJJIIJGJIJJIIIJJIIJJJJIJIJIJIIIIHBB>?BDDDDD<>CCCCDDDDCBDDDD@BCCCDDDDDD?BBB<B@@AA:CD8**  ****************************************************************************  ****** qblast alignment results for example read pair shown above ******  ****************************************************************************  **68144 -> 68244 "NCBI wgs database>gi|540136381|gb|AMFB01000030.1| Bradyrhizobium sp. DFCI-1"**  **Mate A: GCGAATCGAATACTTTCAGCTTCACCGGCACGTCCTTGCCGTCGAGCGTGACGGCGTCGATATCGGCCTCGAGCTCGCGGAGCAGCGCGTTGTGCTTGTCG**  **90% |||| ||||| ||||||||||||||||||||||||| |||||||||||| || |||||||| ||||||||||||||||| |||| | |||||||||||||**  **NCBI wgs:GCGAGTCGAACACTTTCAGCTTCACCGGCACGTCCTGGCCGTCGAGCGTCACCGCGTCGATGTCGGCCTCGAGCTCGCGCAGCACCTTGTTGTGCTTGTCG**  **Best homology:**  **90%: Bradyrhizobium sp. DFCI-1(taxid:1230476)**  **89%: Bradyrhizobium liaoningense(taxid:43992)**  **89%: Bradyrhizobium sp. CCBAU 15635(taxid:1128180)**  **88%: Bradyrhizobium sp. CCGE-LA001(taxid:1223566)**  **Gap between mates: 126 bases**  **68371 -> 68471 "NCBI wgs database>gi|540136381|gb|AMFB01000030.1| Bradyrhizobium sp. DFCI-1"**  **Mate Brc:TCCCAGATGTGGGCGTTGGTGTAGGCCTCCTCGAGATCCATCTCCGCAAGCGCCGGGATGCGCTTGAGATAAGGCAGCGGCGGCATGTTCATGATGGAGAG**  **87% ||||| | |||||||||||||||||||||||||| ||||| | || ||||| ||||| ||||| ||||| ||||||||||||||||||||||| |||||**  **NCBI wgs:TCCCACACCTGGGCGTTGGTGTAGGCCTCCTCGAGGTCCATGCCGGCGAGCGCGGGGATCCGCTTCAGATAGGGCAGCGGCGGCATGTTCATGATCGAGAG**  **Best homology:**  **90%: Bradyrhizobium genosp. SA-4(taxid:508869)"**  **89%: Bradyrhizobiaceae bacterium SG-6C(taxid:709797)**  **89%: Afipia clevelandensis(taxid:1034)**  **88%: Methylobacterium sp. GXF4(taxid:1096546)**  **87%: Bradyrhizobium sp. DFCI-1(taxid:1230476)** |
| --- |
